# Bioactive Component Screening and Mechanistic Study of the Anti-Diabetic Activity of *Lophatherum gracile* Brongn Extract

**DOI:** 10.3390/cimb47090779

**Published:** 2025-09-19

**Authors:** Rong Wang, Xuefeng Liu, Kuan Yang, Shaojing Liu, Lili Yu, Yunmei Chen, Nana Wang, Yaqi Hu, Bei Qin

**Affiliations:** 1 Xi’an Key Laboratory for Research and Development of Innovative Multi-Target Anti-Hypertensive Drugs, Xi’an Medical University, Xi’an 710021, Chinayangkuan@xiyi.edu.cn (K.Y.); liushaojingbmgw@163.com (S.L.);; 2 Xi’an Innovative Anti-Hypertensive Drugs International Science and Technology Cooperation Base, Xi’an Medical University, Xi’an 710021, China; 3 Institute of Drug Research, Xi’an Medical University, Xi’an 710021, China; 4 College of Pharmacy, Xi’an Medical University, Xi’an 710021, China; 5 Shaanxi Institute of Food and Drug Control, Xi’an 710021, China; liu3alxf@163.com

**Keywords:** *lophatherum gracile* brongn, bioactive components, type 2 diabetes mellitus, gut microbiota

## Abstract

Type 2 diabetes mellitus (T2DM), a metabolic disorder defined by glucose and lipid metabolism dysregulation, has become a major global health issue. Hence, effective measures to prevent T2DM are urgently required. *Lophatherum gracile* Brongn (LGB) has been used in managing diabetes-related systemic diseases. However, the hypoglycemic bioactive components in LGB and the mechanisms underlying their hypoglycemic activity remain elusive. The current study sought to characterize the bioactive components of LGB and elucidate its mechanism of action against T2DM. Six common characteristic peaks were identified from six batches of LGB, with 39 characteristic chemical components preliminarily identified. Through component–activity correlation analysis, three functional components—namely isoorientin, orientin, and isovitexin—were selected as key candidates. In T2DM mice, LGB effectively improved glucose and lipid metabolic dysfunction. Untargeted metabolomics analysis revealed that LGB modulated pathways related to lipid and carbon metabolism. 16S rRNA gene sequencing and targeted metabolomics analysis revealed that LGB decreased the ratio of Firmicutes to Bacteroidetes and increased the abundance of bacterial groups such as Lactobacillales and Bacteroides. Additionally, LGB elevated the levels of SCFAs, specifically acetic and butyric acid. Moreover, LGB alleviated intestinal inflammation and upregulated the expression of tight junction proteins by inhibiting the LPS/TLR4/NF-κB signaling pathway. This study demonstrated that LGB treated T2DM, with isoorientin, orientin, and isovitexin identified as the main contributing components. The hypoglycemic mechanism is linked to the “gut microbiota−SCFAs−inflammatory response” signaling axis.

## 1. Introduction

Type 2 diabetes mellitus (T2DM) refers to a metabolic and endocrine disorder that has emerged as a global health issue [1]. A hallmark of T2DM is elevated blood glucose levels, which arise from either relative inadequate insulin or resistance to insulin. Diabetes can lead to several severe complications, particularly cardiovascular issues, diabetic nephropathy, and diabetic retinopathy. The increasing rate of diabetes is concerning, posing a substantial challenge to social and economic development worldwide. By 2019, the global number of diabetes cases had reached 463 million, with forecasts suggesting that this figure could rise to 578 and 700 million by 2030 and 2045, respectively [2].

The human gastrointestinal tract hosts a wide variety of commensal microorganisms known as gut microbiota (GM), which includes around 500 to 1000 distinct bacterial species. It is estimated that the combined genome of these microorganisms is 150 times more extensive than the human genome [3]. Recent studies have underscored the link between the composition of GM and metabolic disorders, highlighting the essential function of GM in preserving host homeostasis [4]. T2DM is a metabolic disorder strongly associated with GM makeup and variability. Studies have shown that the variety of GM in individuals with T2DM is markedly lower than in the non-diabetic population, which is closely related to insulin resistance (IR) and abnormalities in glucose metabolism [5]. In patients with T2DM, the abundance and diversity of certain bacterial populations are distinct compared to normal populations. These results indicated that altering GM could be a promising approach for enhancing diabetes management.

Medications comprise the main approach for T2DM treatment. In modern healthcare, the available pharmacological treatments for managing diabetes include insulin, insulin sensitizers, α-glucosidase inhibitors, sulfonylureas, and agents that stimulate insulin secretion. Antidiabetic medications available in clinics are linked to several side effects, such as gastrointestinal discomfort [6], metabolic strain on the liver [7], and an increased risk of cardiovascular diseases. Diabetes is a multifaceted metabolic condition, and relying solely on one medication that targets a specific pathway makes it difficult to attain an effective therapeutic outcome. Consequently, traditional Chinese medicine and its active ingredients offer accessible, non-toxic, and safe medications for managing blood sugar and mitigating side effects.

*Lophatherum gracile* Brongn. (LGB for short), which is called “Danzhuye” in Chinese, refers to the leaves of *Lophatherum gracile*. LGB is both a traditional medicine and an edible plant [8]. Notably, LGB has been a crucial component in traditional medicinal practices throughout Asia and has been listed in the Chinese Pharmacopoeia. LGB is well recognized for its effects of “clearing heat and reducing fire, relieving restlessness and thirst, as well as enhancing diuresis and catharsis.” [9]. LGB is abundant in flavonoids, polyphenols, and coumarin lactones, making it a significant source of natural antioxidants. Modern pharmacological research indicates that flavonoids are the primary active constituents found in LGB, demonstrating properties such as antioxidative, anti-inflammatory, and hypoglycemic effects [10]. However, the bioactive components and mechanisms underlying the anti-diabetic effects of LGB remain unclear and warrant further investigation.

We utilized integrated methods, such as chemometrics, correlation analysis, in vitro and in vivo pharmacodynamic assays, 16S rRNA gene sequencing, and metabolomics, to explore the anti-diabetic activities and molecular mechanisms of LGB, in addition to screening its bioactive components. These findings provide valuable insights into the potential of flavonoid-rich LGB as a natural strategy for managing diabetes.

## 2. Materials and Methods

### 2.1. Materials and Reagents

Six batches of *Lophatherum gracile* Brongn were collected from major bamboo-producing regions between June and July 2023. Among the samples, those numbered S1–S3 and S4–S6 were collected from Sichuan and Hunan provinces, respectively. Streptozotocin (STZ, No. 18883-66-4) was obtained from med chem express, Inc. (Monmouth Junction, NJ, USA). The reference substances of caffeic acid (No. 110885-201703, 99.7%), orientin (No. 111777-202003, 98.0%), isoorientin (No. 111974-201401, 94.0%), isovitexin (No. 112098-202401, 94.6%), and vitexin (No. 111687-202306, 99.5%) were provided by the national institutes for food and drug control. Tricin reference substances (No. 0527-RG-0052, 96.7%) were obtained from CATO research chemicals Inc (Guangzhou, China). The antibodies, blood lipid assay kits, and enzyme-linked immunosorbent assay (ELISA) kits were obtained from proteintech group, Inc. (Wuhan, China), Nanjing jiancheng bioengineering institute (Nanjing, China), and Shanghai yuanju biotechnology Co., Ltd. (Shanghai, China), respectively. Metformin (H20023370) was provided by Merck (Nantong, China).

### 2.2. Method of LGB Preparation

Dried leaves of bamboo harvested in various regions were crushed to pass through a 20-mesh sieve and then extracted twice with a 60% ethanol aqueous solvent at a 1:20 (g/mL) solid–liquid ratio for 30 min each time. The extraction solutions were mixed and then concentrated under reduced pressure to obtain the crude LGB. For further enrichment and purification, the crude extract was enriched through AB-8 macroporous adsorption resin. After dry sample loading, pure water was first used as the washing solvent to remove water-soluble impurities, followed by elution with a 65% ethanol aqueous solvent. The eluate fractions were collected, concentrated under reduced pressure, and freeze-dried to acquire LGB powder, with an extraction yield of 12.32–15.63%.

### 2.3. Chromatographic Conditions

HPLC fingerprint analysis was performed using a Waters Arc HPLC system (Waters, Milford, MA, USA) attached to a C_18_ column (4.6 × 250 mm, 5 μm). The mobile phases comprised 0.5% acetic acid in water (A) and acetonitrile (B). The gradient elution conditions were configured as follows: 0–5 min, 85% A; 5–25 min, from 85% to 82% A; 25–35 min, from 82% to 70% A; 35–45 min, from 70% to 50% A; 45–50 min, from 50% to 40% A; 50–55 min, from 40% to 85% A. Detection was performed at a wavelength of 330 nm, with a flow rate of 1.0 mL/min and an injection volume of 10 μL.

Chromatographic peaks were characterized and authenticated via an ultra-performance liquid chromatography–electrospray/quadrupole–time-of-flight tandem mass spectrometry (UPLC-ESI-Q/TOF-MS/MS) system (Waters, Milford, MA, USA) fitted with a C_18_ column (2.1 × 100 mm, 1.8 μm). The mobile phase comprised 0.1% aqueous formic acid (A) and acetonitrile (B) and a flow rate of 0.2 mL/min. The elution gradient was structured as follows: 0–1 min, 5% B; 1–4 min, from 5% to 20% B; 4–20 min, from 20% to 50% B; 20–25 min, from 50% to 80% B; 25–28 min, from 80% to 95% B; 28–30 min, from 95% to 5% B. Mass spectra were recorded within the m/z range of 50–2000 using electrospray ionization operated in alternating positive/negative ion mode. The mass spectrometer was set with the following parameters: a capillary voltage of 2.0 kV, a desolvation temperature of 450 °C, a desolvation gas flow rate of 600 L/h, a cone gas flow rate of 50 L/h, and an ion source temperature of 100 °C.

### 2.4. Determination of Antioxidant Activity In Vitro Using DPPH and ABTS Methods

The 1,1-diphenyl-2-picrylhydrazyl (DPPH) and 2,2′-azino-bis(3-ethylbenzothiazoline-6-sulfonic acid) (ABTS) free radical scavenging assays are commonly used in vitro methods for determining antioxidant activity. In this study, the DPPH and ABTS methods were employed to evaluate the antioxidant activity of the samples.

DPPH assay: 100 μL of sample solution (0.44 to 55 μg/mL) was combined with 100 μL of DPPH solution (0.04 mg/mL). The mixture was incubated at room temperature for 30 min, followed by absorbance measurement at 517 nm to determine the DPPH free radical scavenging activity and inhibition concentration at 50% (IC_50_).

ABTS assay: The mixture was diluted with PBS prior to use to calibrate the absorbance at 734 nm to 0.70 ± 0.02. Then, 100 µL of the sample solution (2 to 50 μg/mL) was mixed with 100 µL of the diluted ABTS^·+^ solution, and the reaction was allowed to proceed for 30 min in the dark. Finally, the optical density was measured at 734 nm to calculate the clearance rate and IC_50_ [11].

### 2.5. Inhibition Activity of α-Glucosidase

Following the methods cited in previous studies [12,13,14], 20 μL of PBS, 30 μL of the sample solution (ranging in concentration from 0.05 to 500 μg/mL), and 30 μL of p-nitrophenyl-α-D-glucopyranoside (5 mmol/L) solution were added to a 96-well plate, which was incubated for 15 min at 37 °C. Then, 30 μL of α-glucosidase solution (700 U/mL) was added, and the mixture was incubated again at 37 °C for 30 min. Afterwards, 50 μL of Na_2_CO_3_ solution was added to terminate the reaction, and the absorbance was measured at 405 nm. The sample (system reacting with the enzyme), sample control (system without the enzyme), blank (system lacking the inhibitor), and blank control (system containing neither the enzyme nor the inhibitor) groups were set up to calculate the clearance rate and IC_50_.

### 2.6. Animal Experimental Design

Thirty-six healthy male C57BL/6 mice, aged four weeks with an average weight of 20 ± 2 g, were sourced from Chengdu dashuo experimental animal Co., Ltd. (Chengdu, China, SCXK (Chuan) 2020-0030). All mice were housed in a barrier facility with a controlled temperature (23 ± 3 °C), humidity (50–60%), and light (12 h daylight cycle). Unlimited access to food and water was provided to support normal physiological functions. Following three days of acclimation, six mice were randomly split and continued the standard diet as the control group (Con). The remaining thirty mice were fed an HFD for four weeks (from weeks 1 to 5). The composition of the HFD is shown in Table S1. After a fasting period, mice received intraperitoneal injections of 120 mg/kg STZ, which was solvated in 0.1 M cold citrate buffer (pH 4.5), while the Con group received an equal volume of 0.1 M citrate buffer. After induction, the level of fasting blood glucose (FBG) was assessed using a glucometer (Sinocare Inc., Changsha, China), with levels exceeding 11.1 mmol/L classified as T2DM models. In the 6th week, thirty T2DM mice were randomly divided into five groups (*n* = 6 per group), which included a T2DM group, a Metformin group as a positive control (Met, 150 mg/kg), and groups receiving high-, medium-, and low-LGB doses (100, 200, and 400 mg/kg). The effective dose of LGB was chosen based on previous research [15]. Starting from the 6th week, mice were treated with the respective drugs through oral gavage for a duration of four weeks. Both Con and T2DM groups were given saline daily. During the intervention, the health status of the mice was identified, with body weight and FBG data recorded once weekly.

### 2.7. Evaluation of the Pharmacodynamic Effects of LGB on T2DM Mice

During the 9th week, an oral glucose tolerance test (OGTT) was conducted. Following a fasting period, all mice received an intragastric dose of glucose (2.0 g/kg), and blood glucose levels were recorded at 0, 30, 60, 120, and 180 min. The area under the curve of OGTT (AUC of OGTT) was calculated according to relevant references to quantify the glucose metabolism trends [16].

Blood samples were acquired through orbital extraction for biochemical assessments, which included measuring lipid metabolism indicators, insulin (INS), glycosylated hemoglobin (GHb), glucagon-like peptide-1 (GLP-1), lipopolysaccharide (LPS), and the contents of diamine oxidase (DAO) and d-lactic acid (D-LA). In addition, the homeostasis model assessment of insulin resistance (HOMA-IR) was used to evaluate insulin resistance. To detect the levels of tumor necrosis factor-α (TNF-α) and interleukin-6 (IL-6) in colonic homogenate, colonic tissue was combined with 0.9% normal saline (1:9, *w*/*v*) to create a tissue homogenate. Indicator detection was carried out on an automated microplate reader (Thermo Fisher Scientific, Waltham, MA, USA). The correlation coefficients of the standard curves for blood lipid detection and the ELISA method were greater than 0.995 and 0.99, respectively, while the coefficients of variation for sample repeatability were less than 5% and 15%, respectively.

Histological evaluation was carried out after the mice were euthanized using cervical dislocation. Liver samples received hematoxylin and eosin (HE), along with periodic acid–Schiff (PAS) and Oil red O staining. For intestinal tissues, HE staining was performed, and the expression level of tight junction proteins (zonula occludens-1, ZO-1) was analyzed through immunohistochemistry.

### 2.8. Non-Targeted Metabolomics Analysis of Fecal Samples

Pre-cooled methanol (300 µL) containing 5 ppm of 2-chloro-L-phenylalanine was introduced to 20 mg fecal samples, which were subsequently homogenized twice with a high-throughput tissue grinder set at 55 Hz for 60 s. After 10 min of ultrasonication, the samples were stored in a refrigerator at −20 °C for 30 min and then underwent centrifugation at 12,000 rpm for 10 min at 4 °C. The resulting supernatant was filtered and applied for ultra-high-performance liquid chromatography–tandem mass spectrometry (UPLC-MS/MS) (Thermo Fisher Scientific, Waltham, MA, USA) analysis. Principal component analysis (PCA), partial least squares discriminant analysis (PLS-DA), and orthogonal partial least squares discriminant analysis (OPLS-DA) were employed to reveal the differences in metabolic patterns among different groups. Functional analysis of metabolic pathways was utilized to elucidate the biological significance of the metabolites.

### 2.9. 16S rRNA Sequencing Analysis

Fecal samples were collected for GM analysis before the animals were euthanized. Fecal samples were submitted to Shanghai personalbio technology Co., Ltd. (Shanghai, China) to reveal GM alterations. A brief description is provided: total DNA extraction was conducted, followed by amplification of the V3–V4 region of 16S rRNA genes. The resulting amplification products were analyzed via 2% agarose gel electrophoresis. Equal quantities of amplicons were combined, and paired-end sequencing (2 × 250 bp) was carried out utilizing the Illumina MiSeq platform with the MiSeq Reagent Kit v3 (Illumina, San Diego, CA, USA) [17].

### 2.10. Determination of Short-Chain Fatty Acid Content

500 μL of water was added to the samples, followed by homogenization using glass beads. The mixture was then centrifuged at 4 °C, and the supernatant was collected. Take 200 μL of supernatant, add 20 μL of 375 μg/mL 4-methylvaleric acid solution (as the internal standard), 100 μL of 15% phosphoric acid and 280 μL of diethyl ether to the mixture, and then collect the supernatant. The content of short-chain fatty acids (SCFAs) in feces was detected using gas chromatography–mass spectrometry (GC-MS) [18]. A Thermo Trace 1300 gas chromatograph (Thermo Fisher Scientific, Waltham, MA, USA) was equipped with an HP-INNOWAX capillary column (30 m × 0.25 mm, 0.25 μm) (Agilent, Santa Clara, CA, USA), using helium as the carrier gas at a flow rate of 1 mL/min. The injection volume was 1 μL, with a split mode (10:1) and an injector temperature of 250 °C. MS detection was performed using an ISQ 7000 system (Thermo Fisher Scientific, Waltham, MA, USA). Temperatures of the ion source and MS transfer line were maintained at 300 °C and 250 °C, respectively. Electron impact ionization mode was used, with an ionization energy of 70 eV, and mass spectrometric data were collected in single-ion monitoring mode. The initial temperature was held at 90°C, then ramped to 120 °C at 10 °C/min, followed by an increase to 150 °C at 5 °C/min, and finally elevated to 250 °C at 25 °C/min with a 2 min hold. The linear ranges of all seven SCFAs were 0.02–500.0 μg/mL, and their limit of quantification was 0.02 μg/mL.

### 2.11. Western Blotting Analysis

Colonic tissue was processed in a lysis buffer that contained protease inhibitors to create a homogenate. Following this step, the supernatant was collected, and protein concentration was measured. The next stage involved separating the proteins via sodium dodecyl sulfate-polyacrylamide gel electrophoresis, after which they were transferred to polyvinylidene fluoride membranes and allowed to incubate with primary antibodies: toll-like receptor 4 (TLR4) (1:2000), nuclear factor kappa-B (NF-κB) p65 (1:2000), and GAPDH (1:10,000) overnight at 4 °C. After a 2 h incubation with secondary antibodies, enhanced chemiluminescence was employed for visualization. Target proteins were quantified using Image J (version 1.37V) software.

### 2.12. Statistical Analysis

The results were expressed as mean ± SEM (standard error of the mean). Statistical evaluations and IC_50_ values were performed using GraphPad Prism (version 10.1.2). All data were tested for normality (Shapiro–Wilk test) and equal variance (Brown–Forsythe test). For the comparisons among experimental groups, one-way ANOVA and Tukey’s multiple-comparison test were utilized, with a *p*-value less than 0.05 deemed statistically significant. Pearson’s and Spearman’s correlation analyses were used to determine the correlations between components and biological activities and between GM and metabolites, respectively.

## 3. Results

### 3.1. HPLC Fingerprint of the LGB Samples

**Figure 1:**
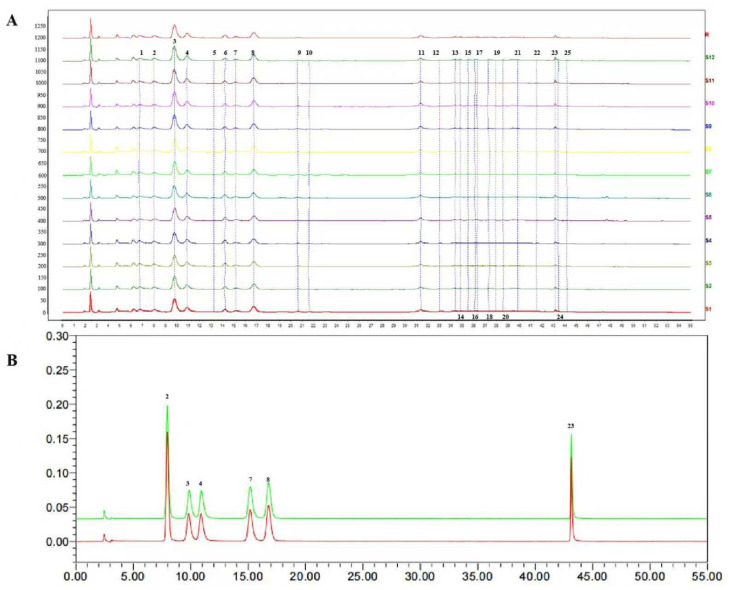
The chromatographic fingerprint and characteristic peaks of six batches of LGB (**A**). The chromatogram of substance benchmarks (**B**). The numbers represented the characteristic peaks in six batches of LGB.

**Figure 2:**
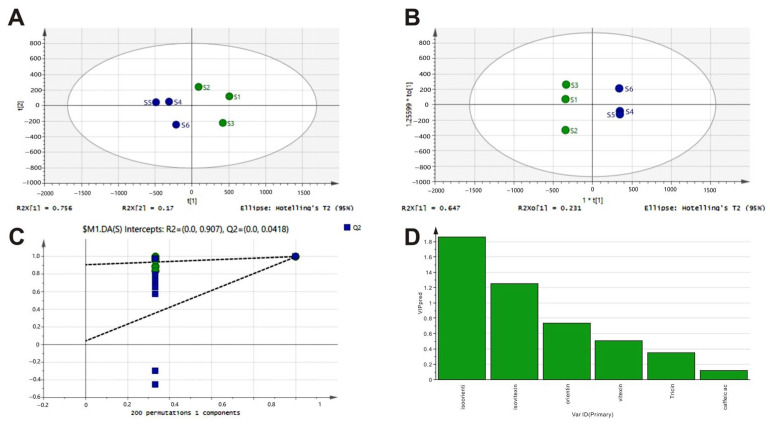
Chemometric analysis of LGB from different geographical origins. (**A**) PCA scores of six batch samples: R2X and Q2 were 0.876 and 0.322, respectively. (**B**) OPLS-DA scores of six common peak areas in samples: R2X, R2Y, and Q2 were 0.996, 1, and 0.997, respectively. (**C**) Plot of 200 permutation test—R2 and Q2 were 0.907 and 0.0418, respectively. (**D**) Plot of variable importance for the projection. Green represented samples collected from Sichuan province, and blue represented those collected from Hunan Province.

HPLC characteristic fingerprints of LGB are shown in Figure 1A. Each sample batch was injected in duplicate, and a total of 25 common chromatographic peaks were identified. Six compounds were identified by comparing their retention times and UV spectra with those of the corresponding reference substances (Figure 1B). The peak area corresponding to 6 common characteristic peaks from six batches of LGB was subjected to chemometric analysis using SIMCA 14.1 software (Umetrics, Umea, Sweden). PCA score plots revealed the overall chemical profile of the samples, showing that samples from different origins were aggregated (Figure 2A). The OPLS-DA and 200 permutation tests were conducted to further investigate the differences between samples and identify the characteristic chemical components that most significantly influenced the geographical origin of LGB. The R2 and Q2 of this model were 0.907 (R2 > 0.9) and 0.0418 (Q2 < 0.05), respectively. The OPLS-DA results indicated that six batches of LGB were clearly divided into two groups, which was consistent with the findings of the PCA. A variable importance for the projection (VIP) value > 1 was used as the screening criterion to determine the key marker components responsible for the differences in chemical composition. As shown in Figure 2D, the order of contribution of differential components was isoorientin > isovitexin > orientin > vitexin > tricin > caffeic acid. Among these, isoorientin (VIP = 1.85) and isovitexin (VIP = 1.26) can serve as chemical markers for quality control.

### 3.2. Qualitative and Quantitative Analysis of Common Peaks in LGB

**Figure 3:**
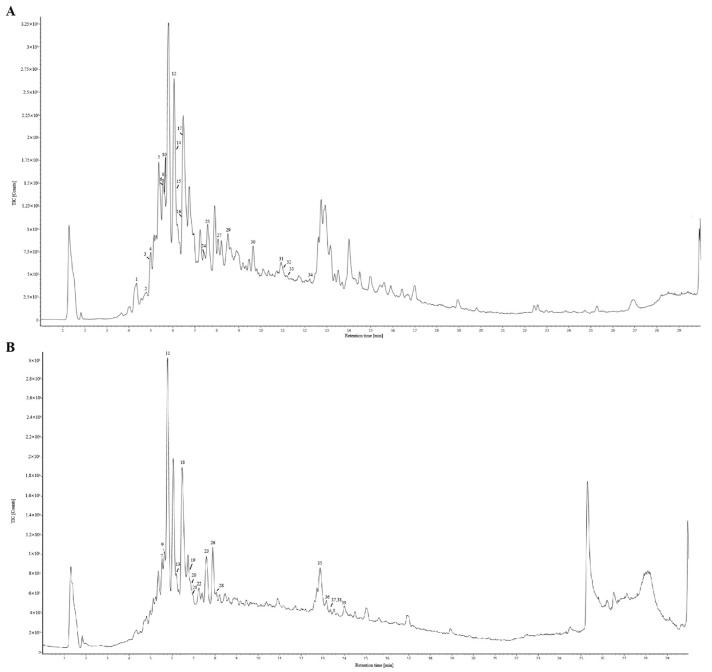
Total ion chromatograms of LGB in negative (**A**) and positive (**B**) ion modes.

Building on Section 3.1, the contents of marker components were quantitatively determined via HPLC. The results are indicated in Table 1. UPLC-ESI-Q/TOF-MS/MS analysis was performed to further identify the ingredients contained in LGB. By analyzing the fragmentation patterns of sample S6 under positive and negative ion modes, a total of 39 components were identified. Through matching with a compound database, 26 and 5 of these were identified as flavonoids and phenolic acids, respectively. The total ion chromatograms are presented in Figure 3. Detailed chemical constituents are listed in Table S2, and the full MS/MS spectra are presented in Figure S1.

### 3.3. Analysis of Pearson Correlations Between Chemical Fingerprints of LGB and Their Biological Activity Profiles In Vitro

It has been widely reported that LGB and its active constituents possess potent antioxidant properties and are proven to confer therapeutic benefits in the treatment of metabolic diseases [19]. The antioxidant capacity was evaluated using DPPH and ABTS free radical scavenging assays. As seen in Table 2, the IC_50_ values of LGB for DPPH and ABTS free radical scavenging activities ranged from 12.02 to 24.18 mg/L and from 3.52 to 12.40 mg/L, respectively. Additionally, the α-glucosidase inhibitory activity of LGB samples was analyzed. Among the LGB samples tested, S6 (IC_50_: 0.11 mg/L) and S2 (IC_50_: 0.34 mg/L) showed the strongest and weakest activity in inhibiting α-Glucosidase. Therefore, we selected the S6 sample for further in vivo activity studies.

Pearson’s correlation analysis was utilized to investigate associations between the main component contents and their biological activities in vitro (Table 3). A strong positive correlation was observed between caffeine acid and antioxidant capacity, as measured with the ABTS assay (r = 0.96, *p* < 0.01). In addition, α-glucosidase inhibitory activity was negatively correlated with isoorientin, orientin, and isovitexin contents (r = −0.96, −0.96, and −0.91, respectively; *p* < 0.05 or *p* < 0.01). These findings suggested that LGB samples containing higher amounts of isoorientin, orientin, and isovitexin possess greater α-glucosidase inhibitory activity.

### 3.4. LGB Enhanced the Pathological State and Blood Glucose Homeostasis in Mice with Diabetes

**Figure 4:**
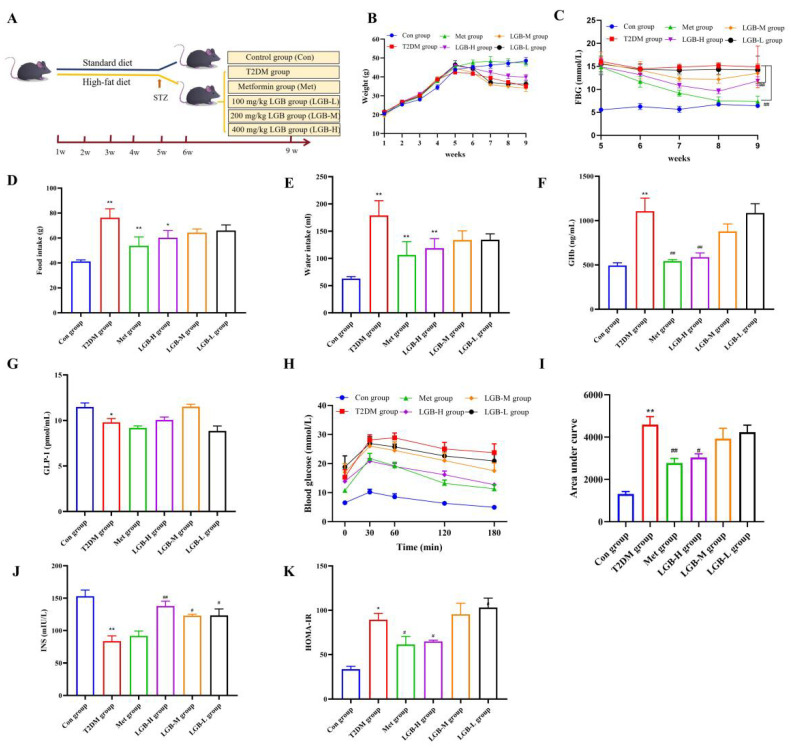
Hyperglycemia in T2DM mice was ameliorated following LGB treatment. (**A**) Experimental design. (**B**) The weight change in mice during the experiment. (**C**) The FBG level. (**D**,**E**) Food and water intake. (**F**) The plasma GHb level. (**G**) The plasma GLP-1 level. (**H**) The curves of OGTT. (**I**) The AUC of OGTT calculated by the trapezoidal rule. (**J**) The plasma insulin level. (**K**) Homeostasis model assessment of insulin resistance (HOMA-IR). *n* = 6, * *p* < 0.05, ** *p* < 0.01 versus the Con group, ^#^ *p* < 0.05, ^##^ *p* < 0.01 versus the T2DM group.

By the fifth week, a diabetic mouse model had been successfully created. Compared to the Con group, the FBG levels in the diabetic mouse model showed a notable rise. LGB administration progressively reduced the symptoms of polydipsia, polyphagia, polyuria, and weight loss in diabetic mice. At the end of 9 weeks, the weights in the Met group were similar to those in the Con group. The LGB-H group showed a significantly higher weight than the T2DM group, with an average increase of 13.4%, which was statistically significant (*p* < 0.05) (Figure 4B). The results of food and water intake are shown in Figure 4D,E.

FBG and GHb are widely recognized as key indicators for measuring long-term glycemia in patients with T2DM [20]. In addition, GLP-1, as a gut hormone, can exert glucose-dependent stimulation of insulin secretion, inhibit food intake, and reduce gastric emptying [21]. FBG and GHb results are presented in Figure 4C,F. These findings revealed that the impact of LGB on reducing FBG and GHb is dose-dependent. Compared with the T2DM group, both the LGB-H and Met groups showed significantly reduced FBG and GHb levels during 6–9 weeks. However, LGB and Met had no effect on the GLP-1 levels (Figure 4G). The OGTT is currently widely utilized for evaluating impaired glucose tolerance and diagnosing T2DM [22]. In the T2DM group, the highest blood glucose level was seen at 60 min, while in the other administration groups, the peak appeared at 30 min. Additionally, compared to the T2DM group, both the Met and LGB-H groups showed a notable decrease in the AUC in the OGTT (Figure 4H,I, *p* < 0.05). Figure 4J illustrates the impact of LGB on INS, and the LGB intervention led to a 48.19–65.06% increase compared with the Con group. As shown in Figure 4K, the LGB-H group showed significantly reduced HOMA-IR values compared with the T2DM group.

### 3.5. LGB Alleviated Lipid Profile Dysregulation and Mitigated Hepatic Injury in Diabetic Mice

**Figure 5:**
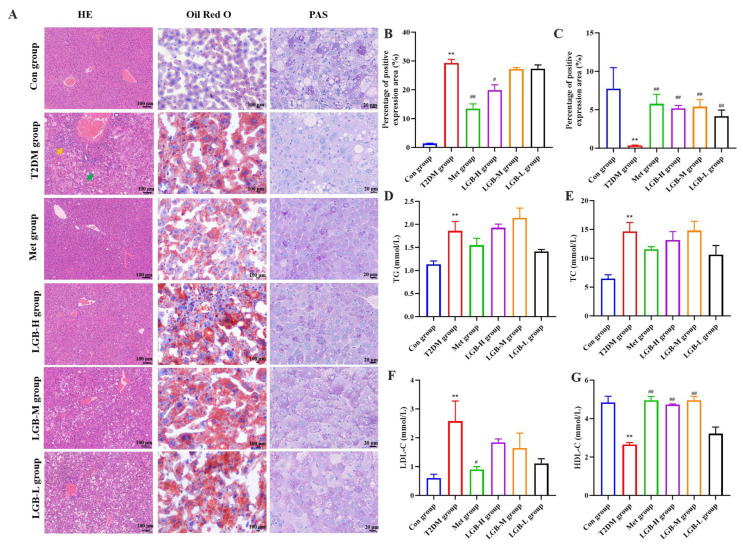
Effects of LGB administration on pathological features and lipid metabolism in T2DM mice. (**A**) Typical images from liver sections stained with HE, OilRedO, and PAS. (**B**) Lipid accumulation score. (**C**) PAS staining corresponding score. (**D**–**G**) The plasma lipid levels. *n* = 6, ** *p* < 0.01 versus the Con group, ^#^ *p* < 0.05, ^##^ *p* < 0.01 versus the T2DM group. The green arrow indicated hepatocellular steatosis, while the yellow arrow denoted fibrous tissue proliferation.

As a long-term metabolic condition, diabetes may disrupt fat metabolism, leading to symptoms of hyperlipidemia. Therefore, we assessed lipid metabolism, and the data indicated that LGB could markedly enhance the HDL-C level in T2DM mice while having no significant effect on the levels of TG, TC, and LDL-C (Figure 5D–G). We further investigated the effect of LGB on the liver’s pathological structure. HE and Oil Red O staining demonstrated that compared with the Con group, the pathological liver structure in the T2DM group exhibited lipid accumulation, hepatocellular steatosis, fibrous tissue proliferation, and inflammatory cell infiltration. However, treatment with Met and high-dose LGB alleviated these pathological symptoms. In addition, the liver is the primary organ for glucose production and storage and plays a crucial role in preserving glucose homeostasis by regulating glycogen synthesis, glycogenolysis, and gluconeogenesis pathways [23]. PAS staining results demonstrated that the area of hepatic glycogen was significantly diminished in the T2DM group. In contrast, hepatic glycogen area was notably elevated after LGB intervention in a dose-dependent manner. In conclusion, these findings suggested that LGB could improve lipid metabolism disorders in diabetic mice.

### 3.6. LGB Enhanced Fecal Metabolic Profile in Mice Suffering from T2DM

**Figure 6:**
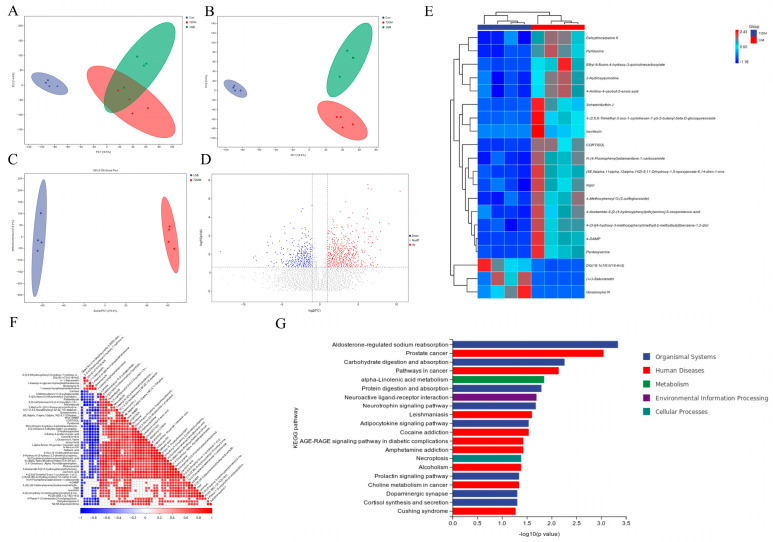
Non-targeted metabolomics and associated pathway analysis in positive ion patterns. (**A**) PCA score scatter plots. (**B**) PLS-DA score scatter plots. (**C**) OPLS-DA score scatter plots. (**D**) Volcano plots for screening differentially abundant metabolites. (**E**) Hierarchical clustering heat map. (**F**) Correlation analysis of differential substances. (**G**) KEGG enrichment analysis for differentially abundant metabolites.

A non-targeted metabolomics analysis between the Con, T2DM, and LGB-H (hereafter referred to as the LGB) groups was conducted to investigate the impact of LGB treatment on the fecal metabolic profile. The total ion chromatograms for different quality control samples showed significant overlap, as demonstrated in Figure S2. PCA enables a more efficient differentiation of metabolites across samples. When the sample points are closely grouped, it signifies a higher degree of similarity in the metabolite types and their concentrations; on the other hand, a broader dispersion implies a more pronounced variation in their overall metabolic levels [24]. In Figure 6A and Figure S3A, the samples from the Con group were closely clustered, distinctly separating them from the T2DM and LGB groups. Similarly, notable separation was observed in the PLS-DAs between the Con and T2DM groups, as well as between the LGB and T2DM groups (Figure 6B and Figure S3B). To further investigate the alterations in metabolites following LGB treatment, OPLS-DA was conducted to highlight the differences between the LGB and T2DM groups. There was a complete separation between the LGB and T2DM groups (Figure 6C and Figure S3C). The results indicated that LGB treatment resulted in a distinct variation in the metabolic profile compared to the T2DM group.

The volcano plots visually illustrate the different metabolites with VIP ≥ 1 and *p* values < 0.05 between the LGB and T2DM groups. In total, 151 metabolites showed considerable upregulation, while 42 metabolites displayed notable downregulation in positive ion modes, and 155 and 37 metabolites were upregulated and downregulated in negative ion modes, respectively (Figure 6D and Figure S3D). The results of hierarchical cluster analysis showed that samples in the same group were clustered together, indicating a degree of consistency within the group (Figure 6E and Figure S3E). Figure 6F and Figure S3F show Spearman’s correlation between different metabolites. KEGG pathway enrichment analysis was used to analyze the metabolic pathways involved with different metabolites. The findings demonstrated that treatment with LGB led to notable changes in metabolic pathways linked to the AGE-RAGE signaling pathway in diabetic complications, carbohydrate and protein digestion and absorption, lipid metabolism, the citrate cycle, carbon metabolism, and the glucagon signaling pathway. Non-targeted metabolomics and associated pathway analysis in negative ion patterns are presented in Figure S3.

### 3.7. LGB Improved Gut Microbiota Dysbiosis in T2DM Mice

**Figure 7:**
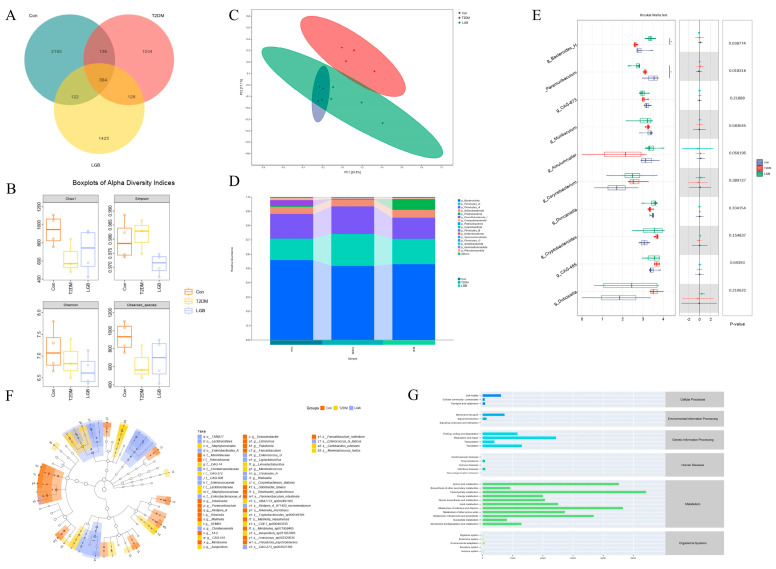
Impact of LGB on gut microbiota in T2DM mice. (**A**) Venn diagram of OTUs. (**B**) The Chao1, Shannon, Simpson, and Observed species indices of gut microbiota. (**C**) PCoA based on Bray–Curtis. (**D**) Relative abundance of gut microbiota at the phylum level. (**E**) Inter-group variations in gut microbiota based on Kruskal–Wallis test. (**F**) Taxonomic cladogram generated from LEfSe (LDA score > 2). (**G**) Enrichment analysis of differential metabolites based on KEGG pathway.

The effect of LGB on GM was examined using 16S rRNA gene sequencing techniques. A Venn diagram of the GM is shown in Figure 7A. In total, 2815, 1662, and 2039 operational taxonomic units (OTUs) were acquired from the Con, T2DM, and LGB groups, respectively. Alpha diversity, which is assessed by indices such as Chao1, Observed species, Shannon, and Simpson, refers to the variety within specific areas or ecosystems. The Chao 1 and Observed species indexes in the T2DM group were lower than those in the Con and LGB groups, indicating reduced GM diversity in T2DM mice. Following LGB treatment, GM diversity increased (Figure 7B). β-diversity was determined via principal coordinate analysis (PCoA) based on Bray–Curtis dissimilarity. There was a significant difference between the Con and T2DM groups, while the LGB group overlapped with the Con group, indicating that LGB could restore the GM structure.

Firmicutes, Bacteroidota, and Actinobacteriota were determined to be the main GM groups at the phylum level. The Firmicutes-to-Bacteroidetes (F/B) ratios of the Con and LGB groups were 0.60 ± 0.083 and 0.62 ± 0.095, respectively, which showed significant differences compared with the T2DM group (0.83 ± 0.11) (*p* < 0.05). The Kruskal–Wallis test was employed to assess the intergroup variations in GM at the genus level among three experimental groups. The abundance of Bacteroides_H significantly decreased, and Paramuribaculum in the T2DM group significantly increased. After the LGB intervention, the abundances of the above two bacterial groups were significantly increased, and they became the dominant phyla (*p* < 0.05).

Linear discriminant analysis (LDA) effect size (LEfSe) was applied to identify statistically significant biomarkers between groups (LDA  > 2 and *p* < 0.05 as a threshold). In total, 46 bacterial biomarkers were identified (Figure 7F). In the Con group, 18 differentially abundant species were identified, including those classified under Marinifilaceae and Rikenellaceae at the family level, as well as Odoribacter, Paramuribaculum, and Mailhella at the genus level. In the T2DM group, the dominant GM at the family level was Staphylococcaceae, while Avispirillum and Mammalicoccus were predominant at the genus level. In the LGB group, Lactobacillales and Ligilactobacillus at the order and genus levels, respectively, were identified as the major differentially abundant taxa. KEGG pathway analysis was performed on the obtained differential metabolites, and the metabolic pathways affected by LGB intervention in T2DM mice were screened with *p* < 0.05 as the standard. The results showed that the most significantly affected KEGG secondary metabolic pathway categories after LGB intervention were amino acid metabolism, carbohydrate metabolism, lipid metabolism, etc. (Figure 7G).

### 3.8. LGB Altered the Microbial Metabolites in the Feces of T2DM Mice

**Figure 8:**
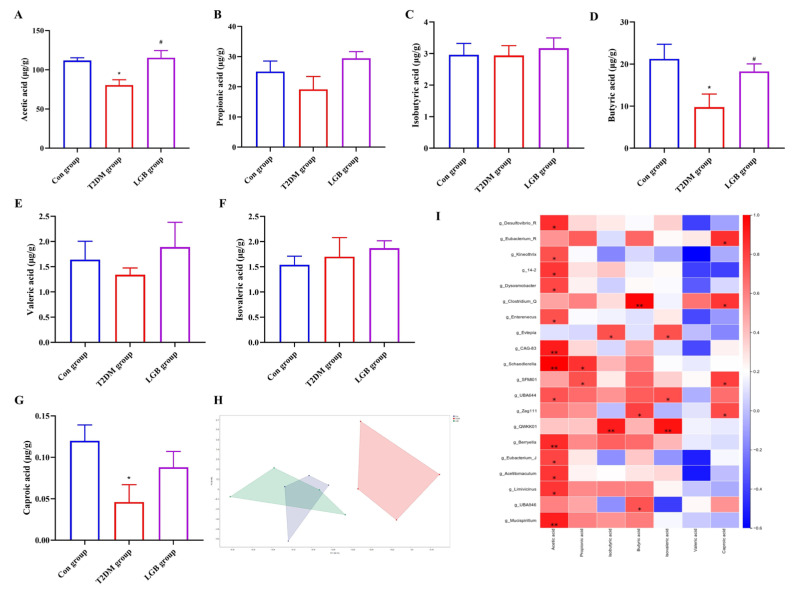
Effect of LGB on SCFAs in the feces of T2DM mice. (**A**–**G**) The contents of the seven SCFAs. (**H**) PCA plot of SCFAs. (**I**) Correlation study of gut microbiota and SCFAs. *n* = 4, * *p* < 0.05, ** *p* < 0.05 versus the Con group, ^#^ *p* < 0.05 versus the T2DM group.

SCFAs are microbial metabolites generated through the metabolic processing of exogenous dietary constituents by GM and act as mediators in regulating host metabolic phenotypes via GM. We first employed a PCA to evaluate the SCFA levels in the fecal samples from mice in each group. The results showed an overlap between the Con and LGB groups, while there was a significant difference between the T2DM and LGB groups (Figure 8H). Compared with the Con group, the acetic, butyric, and caproic acid levels in the T2DM group significantly decreased from 111.80 ± 7.06, 21.22 ± 6.98, and 0.12 ± 0.038 μg/g to 80.4 ± 13.72, 9.76 ± 6.23, and 0.046 ± 0.042 μg/g, respectively. After LGB treatment, the contents of acetic and butyric acid increased to 115.39 ± 18.38 μg/g and 18.26 ± 3.57 μg/g, respectively, which showed significant differences compared with the T2DM group (*p* < 0.05). We performed a Spearman’s correlation analysis to explore the potential intrinsic relationships between GM and SCFAs. The results showed that Desulfovibrio, Kineothrix, Dysosmobacter, Enterenecus, Schaedlerella, Berryella, Eubacterium_J, Acetitomaculum, Limivicinus, and Mucispirillum at the genus level exhibited a positive association with acetic acid levels. The abundance of Clostridium_Q was positively correlated with butyric acid. These results indicated that LGB has a strong association with regulating GM and SCFA profiles.

### 3.9. LGB Alleviated Inflammation and Improved Intestinal Barrier Damage in T2DM Mice by Inhibiting the LPS/TLR4/NF-κB Signaling Pathway

**Figure 9:**
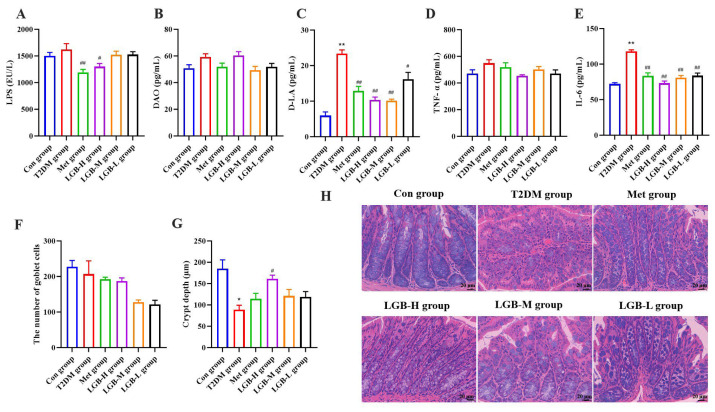
The effect of LGB on inflammation and intestinal pathology. (**A**–**C**) The contents of LPS, DAO, and D-LA. (**D**,**E**) The contents of TNF-α and IL-6. *n* = 6. (**F**) The number of goblet cells. (**G**) Quantitative detection of the crypt depth. (**H**) HE staining of colonic tissue. *n* = 3. * *p* < 0.05, ** *p* < 0.01 versus the Con group, ^#^ *p* < 0.05, ^##^ *p* < 0.01 versus the T2DM group.

In patients with T2DM, elevated blood glucose could disrupt intestinal tight junctions and hasten intestinal barrier degradation [25]. To investigate the protective role of LGB in the intestines of diabetic mice, we initially assessed serum markers of intestinal mucosal injury and measured inflammatory cytokines in homogenates of intestinal tissue, concurrently observing the pathological alterations within the intestinal structures. The results indicated that compared to the Con group, the D-LA level was significantly elevated in the T2DM group, and LGB could significantly reduce D-LA levels in a dose-dependent manner (*p* < 0.05 or *p* < 0.01). However, LGB had no effect on DAO levels. In addition, LGB significantly reduced the LPS level in serum and IL-6 level in tissue homogenates, while it had no significant effect on TNF-α. Colonic pathology analysis showed that LGB significantly increased crypt depth without affecting the number of goblet cells (Figure 9F–H). Research indicated that enhancing the expression of tight junction proteins can safeguard the intestinal mucosal barrier, thereby reducing LPS-induced increases in intestinal permeability [26].

**Figure 10:**
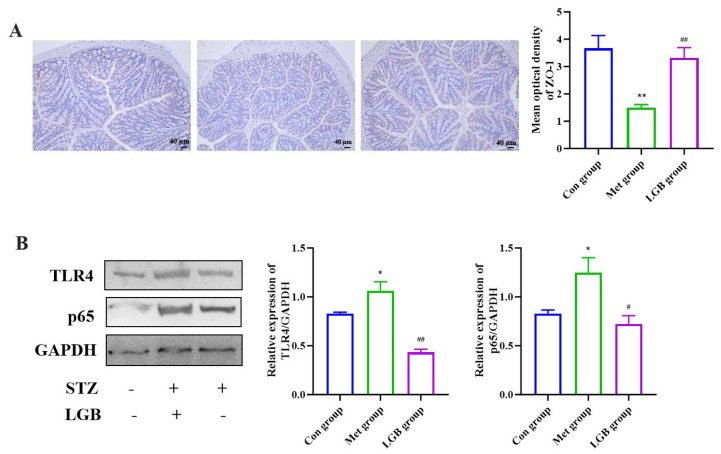
LGB alleviated colonic barrier dysfunction through TLR4/NF-κB pathway. (**A**) Immunohistochemical staining and mean optical density for ZO-1 proteins. (**B**) Protein expression and quantitative analysis of TLR4/NF-κB pathway. *n* = 3, * *p* < 0.05, ** *p* < 0.01 versus the Con group, ^#^ *p* < 0.05, ^##^ *p* < 0.01 versus the T2DM group.

To explore this, we examined ZO-1 levels via immunohistochemistry. Our findings revealed that the T2DM group exhibited lower levels of ZO-1; following treatment with LGB, a significant increase in its expression was observed (*p* < 0.01) (Figure 10A). It is widely accepted that LPS can specifically trigger the TLR4/NF-κB signaling axis, which mediates immune responses and enhances the synthesis of pro-inflammatory cytokines, leading to the development of chronic inflammation [27,28]. To explore the impact of diminished LPS levels caused by LGB on the TLR4/NF-κB signaling pathway and the resulting inflammatory response, we further measured the expression levels of proteins linked to this signaling pathway. The protein levels of TLR4 and NF-κB p65 in the T2DM group were significantly upregulated compared with the Con group. After treatment with LGB, the expression of these proteins was significantly downregulated (*p* < 0.05, Figure 10B). The findings indicated that LGB diminishes inflammatory responses, which, in turn, enhances gut barrier health by blocking the LPS/TLR4/NF-κB signaling pathway.

## 4. Discussion

Flavonoids are among the most important low-molecular-weight phenolic compounds and have been reported to exhibit various biological activities, including antioxidant, hypoglycemic, lipid-lowering, and anti-inflammatory effects [29]. Compared with standard antidiabetic drugs, medicinal plants rich in flavonoids tend to exhibit a lower risk of adverse reactions. Consequently, the present study sought to explore the role of bamboo leaves and their primary phytochemical constituents—flavonoids—in diabetes treatment.

In this study, the quality of six bamboo leaf varieties collected from two regions was evaluated. An HPLC fingerprint was established using RP-HPLC, and chemometric analyses were performed to screen for candidate marker compounds in bamboo leaves. Six common characteristic peaks were selected, including five flavonoids and one phenolic acid. PCA is a multivariate analytical method whose primary objective is to reduce dataset dimensionality while minimizing information loss [30]. The six LGB samples were clustered into two distinct groups via PCA, revealing variations in the quality of bamboo leaves from the two origins. OPLS-DA is recognized for enhancing relevant information while reducing irrelevant noise in datasets [31]. By integrating VIP screening, the OPLS-DA model identified isoorientin and isovitexin as potential marker compounds. Isoorientin and isovitexin, categorized as C-glycosyl flavonoids, are recognized as key pharmacologically active components of LGB, which have demonstrated beneficial effects on metabolic disturbances.

The antioxidant activity in vitro and anti-α-glucosidase activity of LGB samples were subsequently investigated. Additionally, Pearson’s correlation analysis was performed to explore the associations between the compound profiles and their activities based on correlation coefficients, which reflect the degree of association between components and activities, and statistical significance tests, which determine whether such associations are statistically meaningful. The results showed that the IC_50_ of LGB for ABTS radical scavenging exhibited a significant positive correlation with caffeic acid. In contrast, the other five flavonoids showed a certain negative correlation with the IC_50_ for α-glucosidase inhibition. Among them, the contents of isoorientin, orientin, and isovitexin were significantly negatively correlated with the IC_50_ of α-glucosidase inhibition, suggesting that the contents of isoorientin, orientin, and isovitexin could serve as indicators for evaluating the hypoglycemic activity of LGB.

The two primary hallmarks of T2DM are hyperglycemia and insulin resistance. We evaluated the hypoglycemic activity of LGB using FBG and GHb. Compared with the model mice at week 9, the FBG and GHb levels decreased by 20.73% and 46.92%, respectively. GLP-1, an incretin hormone secreted by intestinal L-cells, serves a crucial role in regulating blood glucose homeostasis [32], including inhibiting food intake, reducing gastric emptying, and stimulating insulin secretion [21]. We found that LGB promoted insulin secretion but did not affect GLP-1 levels. The OGTT is the preferred initial screening method for T2DM. Compared with model mice, the AUC in the OGTT in the LGB-H group was reduced by 33.83%. HOMA-IR can reflect insulin resistance status. The HOMA-IR increased in model mice, while the high-dose LGB group showed a significantly reduced HOMA-IR, by 27.35%. These results suggested that LGB can regulate blood glucose and improve insulin resistance without affecting GLP-1 levels.

Previous studies confirmed that disturbances in lipid metabolism are closely associated with insulin resistance and hyperglycemia [33]. Meanwhile, as a critical organ involved in lipid synthesis and metabolism, the liver is deeply involved in preserving lipid homeostasis. In T2DM mice, liver function and lipid metabolism are impaired, resulting in abnormalities in the morphology and structure of the hepatocytes, as well as hepatic inflammatory infiltration and steatosis. In contrast, LGB significantly increased HDL-C levels, alleviating lipid accumulation, steatosis, fibrous tissue proliferation, and inflammatory cell infiltration. The liver fulfills a vital role in regulating the balance between glycogenolysis and gluconeogenesis. PAS staining results demonstrated that the high-dose LGB group markedly augmented the hepatic glycogen area in T2DM mice. These results suggested that LGB can regulate lipid metabolism disorders, thus offering further therapeutic benefits in diabetes management.

In this study, a combined approach of gut microbiomics and metabolomics was employed to reveal the effects of LGB on GM and endogenous metabolites in T2DM mice. First, untargeted metabolomics analysis showed that LGB significantly affected lipid and carbon metabolism pathways. These two pathways are strongly linked to SCFA synthesis, with SCFA synthesis and metabolism being tightly associated with T2DM [34]. Hence, it is hypothesized that LGB may alleviate abnormal glucose and lipid metabolism in T2DM mice by regulating SCFA biosynthesis. Furthermore, SCFAs are metabolic products of GM and can also participate in GM regulation. We performed gut microbiomics and targeted metabolomics analyses on GM and metabolic products.

Studies have shown that the occurrence and progression of T2DM are closely related to the composition of Bacteroidetes and Firmicutes. A high F/B ratio is positively correlated with FBG and insulin resistance and also serves as a marker of GM dysbiosis [35]. The results of this study showed that after LGB treatment, the abundance of Bacteroidetes increased while the abundance of Firmicutes decreased, leading to a significant reduction in the F/B ratio. This beneficial effect has been validated across a range of flavonoid compounds [36]. The relative abundance of Bacteroides has been confirmed to be negatively correlated with high blood glucose levels [37]. Paramuribaculum is a microbial group closely associated with T2DM progression. It can significantly reduce hyperglycemia in T2DM mice and promote an increase in the abundance of beneficial bacteria [38]. In our study, the LGB intervention increased the abundance of Bacteroides and Paramuribaculum. Previous studies revealed that a reduced F/B ratio can increase SCFA levels [39], among which Bacteroidetes are particularly important for fermenting complex carbohydrates into SCFAs [40]. Consistent with these findings, after LGB treatment, SCFA levels, especially levels of acetic and butyric acid, were significantly increased. Correlation analysis showed that the levels of acetic and butyric acid were significantly positively correlated with the abundance of certain genera. For example, Eubacterium and Clostridium are known butyric-acid-producing bacteria, while Acetitomaculum is a known acetic-acid-producing bacterium. These results suggest that LGB can regulate the GM of model mice, promote the production of SCFAs, and improve metabolic homeostasis in T2DM.

Several studies have suggested that inflammation is an important characteristic of diabetes. Changes in the composition of GM and their metabolites induce GM imbalance. Such alterations in the microbiota not only compromise the intestinal barrier but also elevate the abundance of Gram-negative bacteria, which in turn facilitates the biosynthesis of LPS—a key structural constituent of the outer membrane of Gram-negative bacteria [41]. The impairment of intestinal barrier function increases intestinal permeability, allowing LPS to translocate across the gut, and affects metabolic organs such as the pancreas and adipose tissue, thereby triggering chronic inflammation [42]. It has been reported that high levels of LPS are capable of being specifically recognized by TLR4, promoting the nuclear translocation of NF-κB and regulating the expression of inflammatory factors, and ultimately result in abnormal glucose metabolism and IR [43]. Inhibiting the LPS/TLR4/NF-κB pathway is a potential therapeutic approach for reducing inflammation in diabetic patients. This study demonstrated that LGB can reverse the dysregulation of GM composition in diabetic mice. On this basis, we evaluated the effects of LGB on intestinal mucosal injury markers, intestinal pathological changes, inflammatory factor levels, and the activation status of the TLR4/NF-κB pathway. We first evaluated serum markers of intestinal mucosal injury, including LPS, DAO, and D-LA. The results showed that compared with the T2DM group, the high-dose LGB group reduced LPS and D-LA levels by 19.77% and 55.69%, respectively. The evaluation of inflammatory indicators showed that different dose groups of LGB reduced the IL-6 levels by 28.88–37.81%. ZO-1 is an epithelial tight junction protein whose primary function is to maintain intestinal barrier permeability. The ZO-1 protein expression showed that the protein expression in the T2DM group was significantly reduced, while LGB administration significantly increased the expression. We detected the effect of LGB on the protein levels of TLR4 and NF-κB p65 using Western blotting. The results showed that LGB significantly downregulated the protein expression of TLR4 and NF-κB p65 in the colonic tissue. The above results demonstrate that LGB ameliorates intestinal barrier function by suppressing the TLR4/NF-κB pathway, decreasing intestinal inflammation levels, and enhancing the expression of the tight junction protein ZO-1.

Although this study has revealed the active ingredients and mechanisms underlying the hypoglycemic effect of LGB, several limitations remain. First, the focus of this study was on the hypoglycemic effect of LGB, with a lack of safety data for LGB. The toxicological properties of LGB, particularly its long-term toxicity, represent a direction for future research. Second, the composition of LGB is complex, with flavonoids being the key components responsible for its biological effects. Accordingly, we performed a quantitative analysis of six marker flavonoids. However, research on the anti-diabetic effects of its specific bioactive components remains insufficient. Therefore, further evaluation of the anti-diabetic efficacy of individual components is warranted to identify the phytochemicals that regulate blood glucose. Third, the Western blot analysis preliminarily revealed that the LPS/TLR4/NF-κB pathway is a potential mechanism underlying LGB’s therapeutic effect on T2DM. However, direct evidence supporting the relationship between this signaling pathway and LGB’s anti-diabetic effects is lacking. In subsequent studies, experimental approaches such as fecal microbiota transplantation or antibiotic intervention should be carried out to clarify the underlying mechanism. Fourth, insulin resistance and relative insulin deficiency are typical characteristics of T2DM. Metformin can improve metabolic insulin resistance, while glucagon-like peptide-1 receptor agonists (GLP-1RAs) have the ability to stimulate insulin release. In the present study, metformin serves as the sole control. Adding GLP-1RAs to the control could enhance the thoroughness of evaluating LGB’s hypoglycemic ability. In addition, the evaluation of the hypoglycemic effect of LGB relied solely on weekly blood glucose measurements in this study, failing to capture the dynamic characteristics of blood glucose changes. Therefore, we will perform continuous glucose monitoring in mice to verify the hypoglycemic effect of LGB in further research.

## 5. Conclusions

Overall, we found that LGB contains 39 common compounds such as flavonoids and phenolic acids and identified isoorientin, orientin, and isovitexin as the effective ingredients responsible for its hypoglycemic effect. The hypoglycemic effect of LGB was confirmed in T2DM mice. LGB can improve glucose tolerance and insulin resistance by alleviating hepatic steatosis and reducing inflammatory cell infiltration, thereby regulating glucose and lipid metabolic dysfunction. Mechanistically, LGB improved the composition and abundance of GM in T2DM mice, elevated SCFA levels, inhibited the LPS/TLR4/NF-κB signaling pathway, alleviated intestinal inflammation, and enhanced the intestinal barrier. Collectively, these findings provide a reference for elucidating the hypoglycemic components and mechanisms of LGB.

## Figures and Tables

**Table 1:** Concentration of marker components in LGB.

Sample	Content of Components (mg/g)					
	Isoorientin	Orientin	Vitexin	Isovitexin	Tricin	Caffeic Acid
S1	117.86 ± 0.79	40.23 ± 0.25	9.40 ± 0.01	36.36 ± 0.21	5.43 ± 0.02	8.61 ± 0.17
S2	97.97 ± 0.49	37.89 ± 0.02	8.94 ± 0.02	32.64 ± 0.05	4.41 ± 0.02	8.62 ± 0.13
S3	112.94 ± 0.38	39.74 ± 0.38	8.76 ± 0.03	33.04 ± 0.17	6.22 ± 0.07	7.01 ± 0.22
S4	117.66 ± 0.47	42.44 ± 0.07	10.09 ± 0.25	38.05 ± 0.19	4.49 ± 0.02	9.11 ± 0.06
S5	128.60 ± 0.34	41.93 ± 0.10	9.59 ± 0.17	37.49 ± 0.14	6.71 ± 0.03	7.10 ± 0.08
S6	128.92 ± 0.72	41.73 ± 0.48	10.23 ± 0.07	39.31 ± 0.18	7.79 ± 0.03	9.16 ± 0.08

**Table 2:** IC_50_ of the free radical scavenging activity and anti-α-glucosidase activity of LGB samples.

Sample	IC_50_ (mg/L)		
	DPPH	ABTS	α-Glucosidase
S1	22.97 ± 1.54	9.50 ± 0.42	0.18 ± 0.03
S2	12.02 ± 1.15	7.42 ± 0.24	0.34 ± 0.04
S3	20.03 ± 1.76	3.52 ± 0.16	0.24 ± 0.02
S4	24.18 ± 2.62	12.40 ± 1.01	0.14 ± 0.07
S5	15.85 ± 1.34	11.63 ± 0.93	0.13 ± 0.03
S6	12.02 ± 1.92	3.67 ± 0.13	0.11 ± 0.01

**Table 3:** Pearson correlation analysis between the contents of components and the IC_50_ of free radical scavenging activity and anti-α-glucosidase activity.

Indicator	IC_50_ (mg/L)		
	DPPH	ABTS	α-Glucosidase
caffeic acid	0.28	0.96 **	−0.037
isoorientin	0.063	0.12	−0.96 **
orientin	0.30	0.34	−0.96 **
vitexin	0.19	0.73	−0.78
isovitexin	0.17	0.58	−0.91 *
tricin	−0.29	−0.16	−0.54

* *p* < 0.05, ** *p* < 0.01.

## Data Availability

Gut microbiota sequencing data have been deposited in the National Center for Biotechnology Information Sequence Read Archive under accession number PRJNA1326909. Metabolomics data have been made available in the National Center for Bioinformation with accession number OMIX011871. Additional supporting data are contained within the article and its Supplementary Materials.

## Supplementary Materials

The following supporting information can be downloaded at https://www.mdpi.com/article/10.3390/cimb47090779/s1.

## Abbreviations

The following abbreviations are used in this manuscript:

T2DM	Type 2 diabetes mellitus
LGB	*Lophatherum gracile* Brongn
GM	Gut microbiota
HE	Hematoxylin and eosin
PAS	Periodic acid–Schiff
STZ	Streptozotocin
INS	Insulin
FBG	Fasting blood glucose
OGTT	Oral glucose tolerance test
HOMA-IR	Homeostasis model assessment of insulin resistance
GHb	Glycosylated hemoglobin
DAO	Diamine oxidase
D-LA	D-lactic acid
TC	Total cholesterol
TG	Total triglyceride
LDL-C	Low-density lipoprotein cholesterol
HDL-C	High-density lipoprotein cholesterol
TNF-α	Tumor necrosis factor-α
IL-6	Interleukin-6
LPS	Lipopolysaccharide
TLR4	Toll-like receptor 4
NF-κB	Nuclear factor kappa-B
PCoA	Principal coordinate analysis
PLS-DA	Partial least squares discriminant analysis
OPLS-DA	Orthogonal partial least squares discriminant analysis
PCA	Principal component analysis
ZO-1	Zonula occludens-1
SCFAs	Short-chain fatty acids
