# Supplementary material for: Bioactive Component Screening and Mechanistic Study of the Anti-Diabetic Activity of Lophatherum gracile Brongn Extract
Source: Curr Issues Mol Biol. 2025 Sep 19;47(9):779. doi: 10.3390/cimb47090779 (PMC12468491; doi:10.3390/cimb47090779)

Component name: caffeic acid

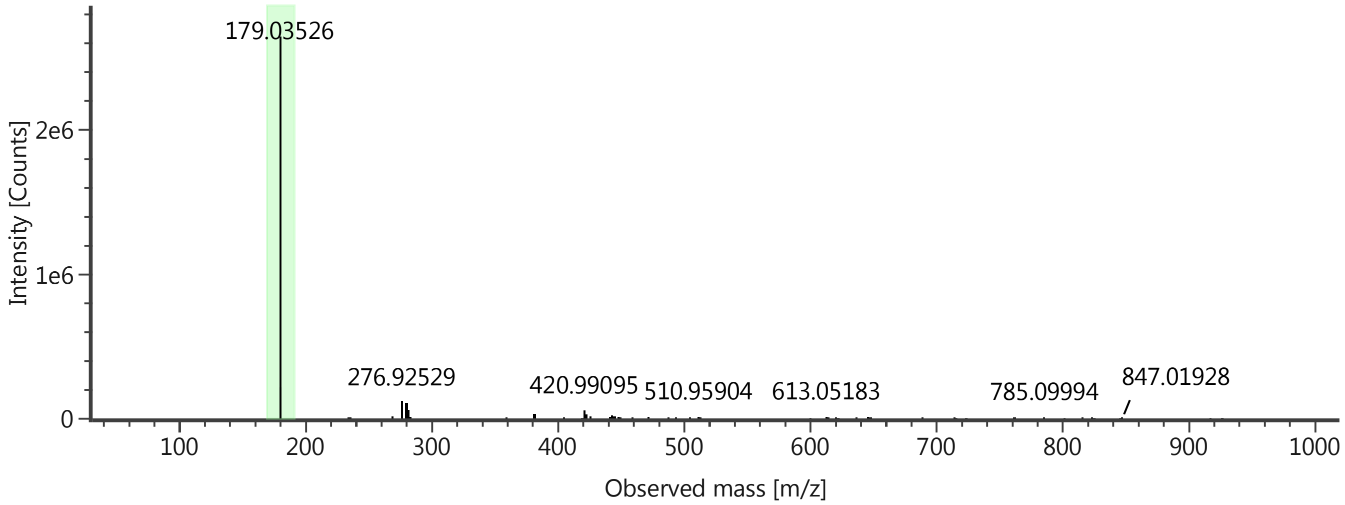

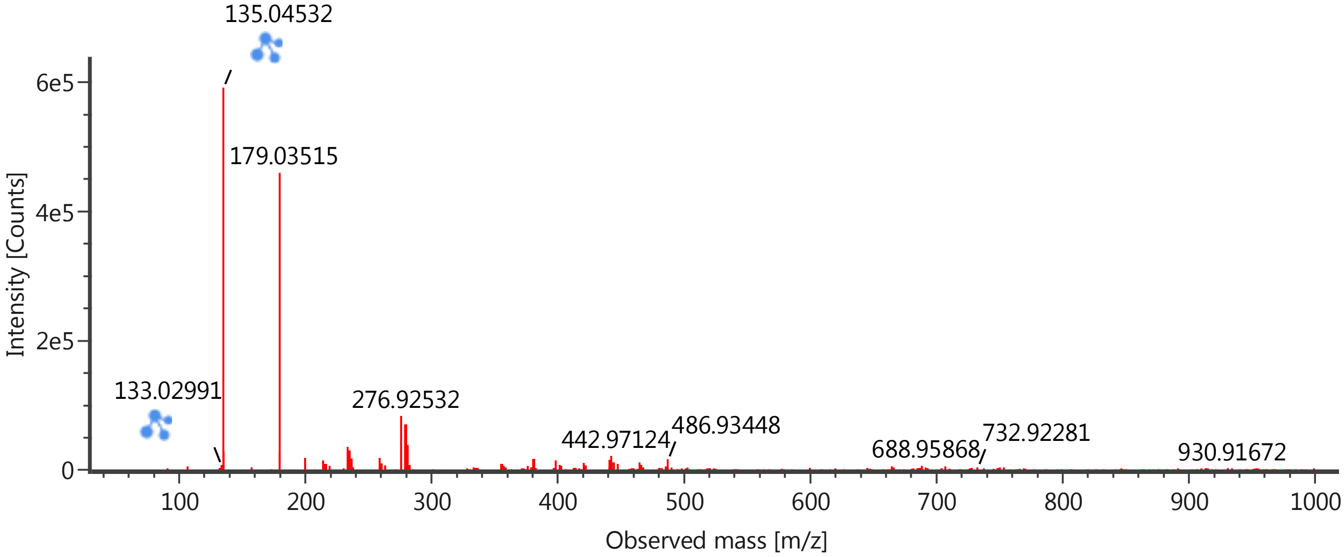

Component name: rutin

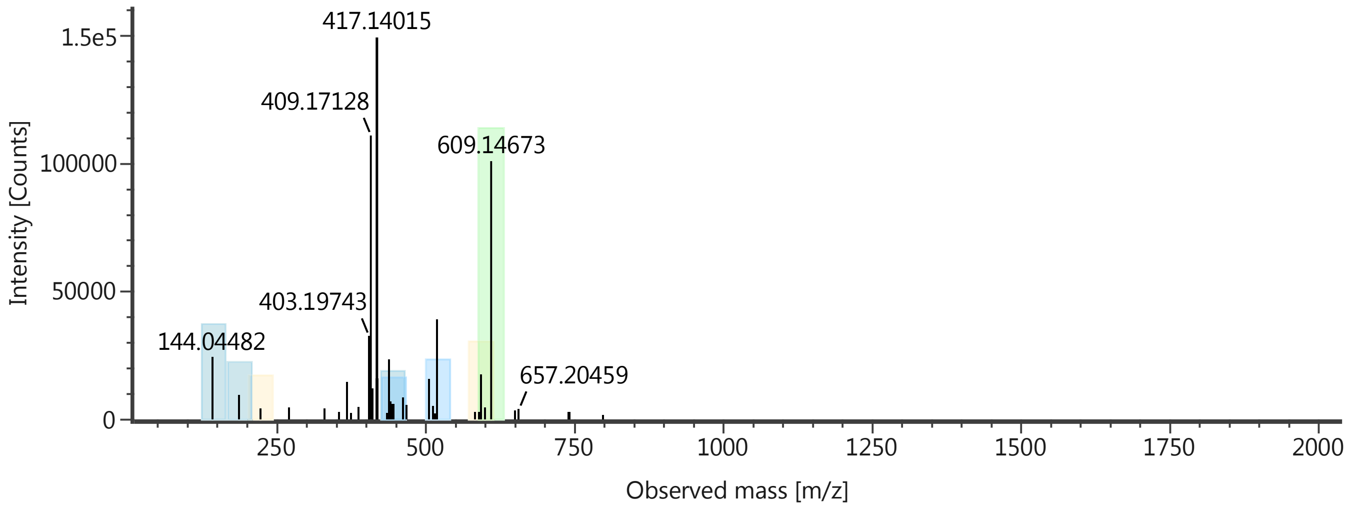

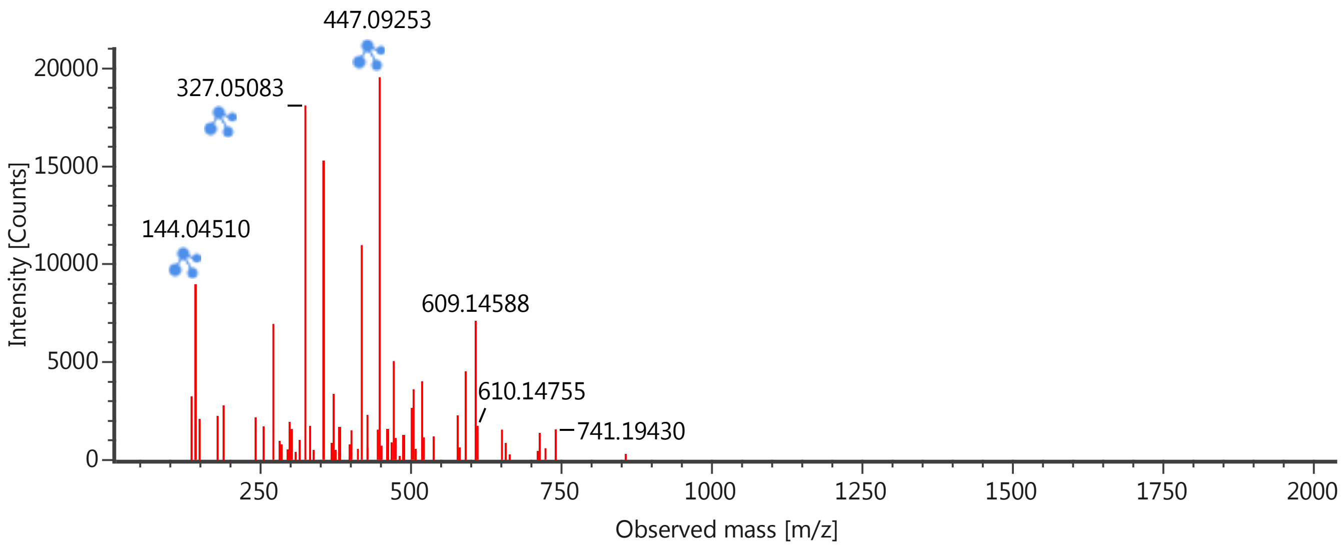

Component name: neochlorogenic acid

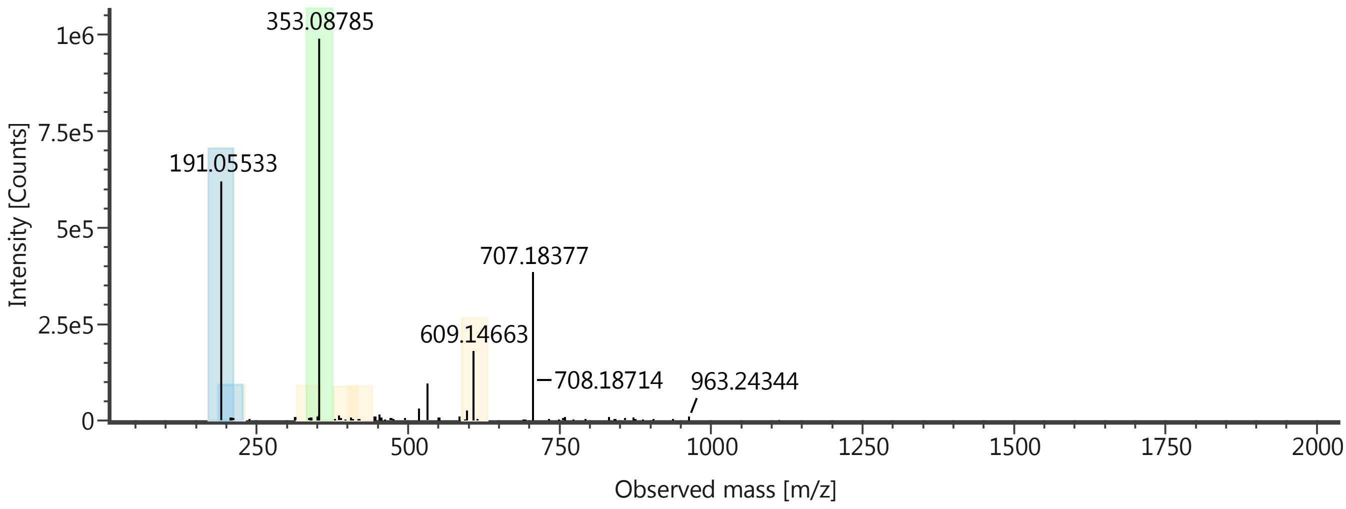

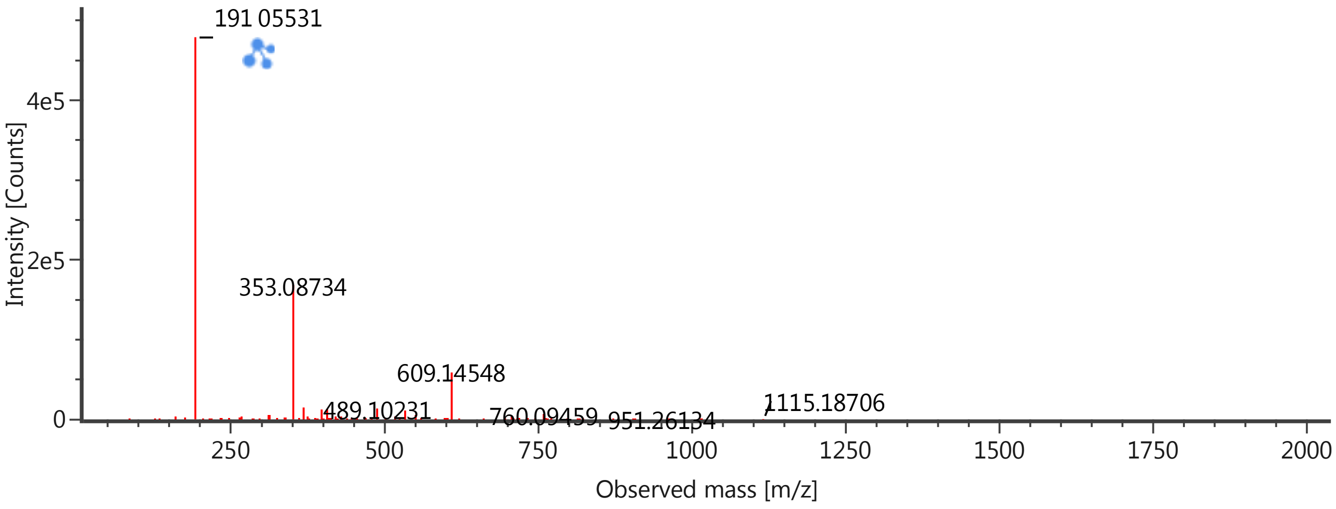

Component name: isoorientin

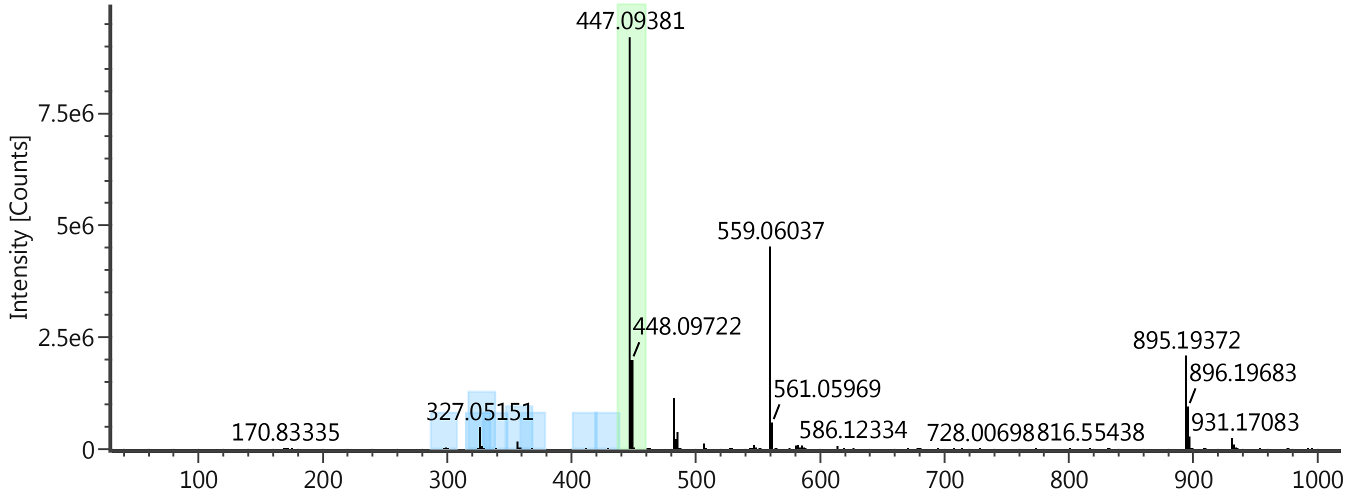

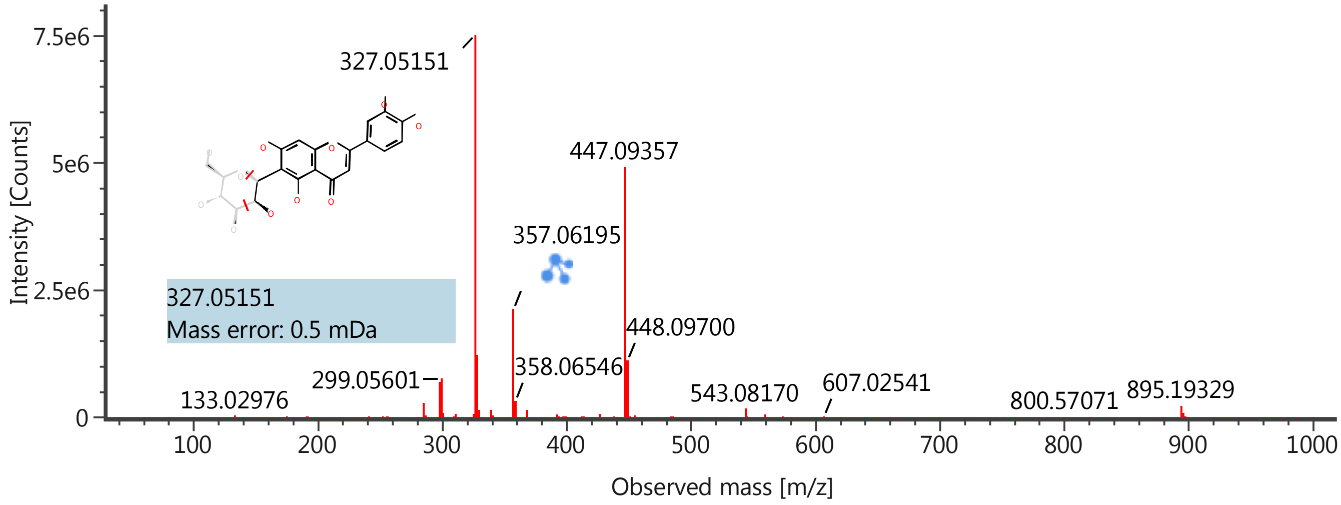

Component name: kaempferol-3-O-β-D-rutinoside

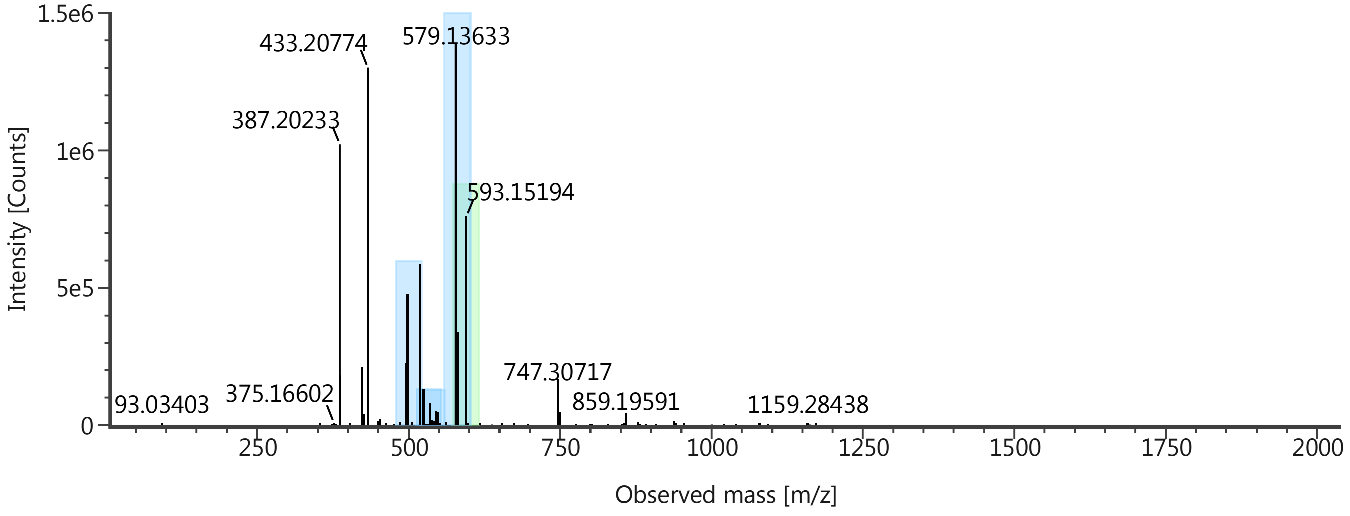

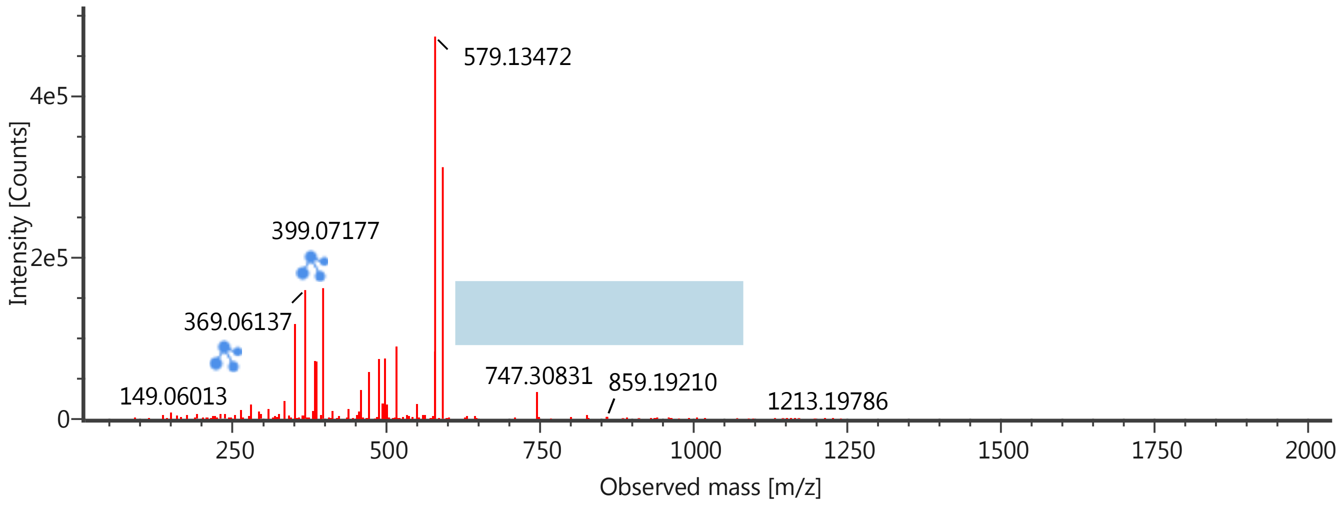

Component name: quercetin-3-O-β-D-glucoside

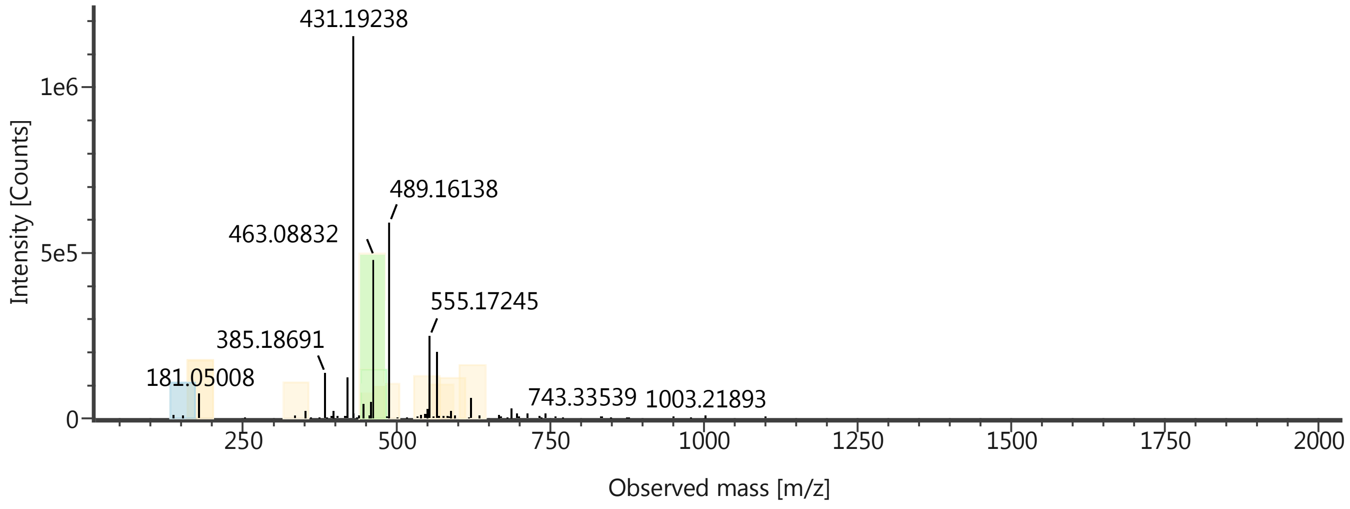

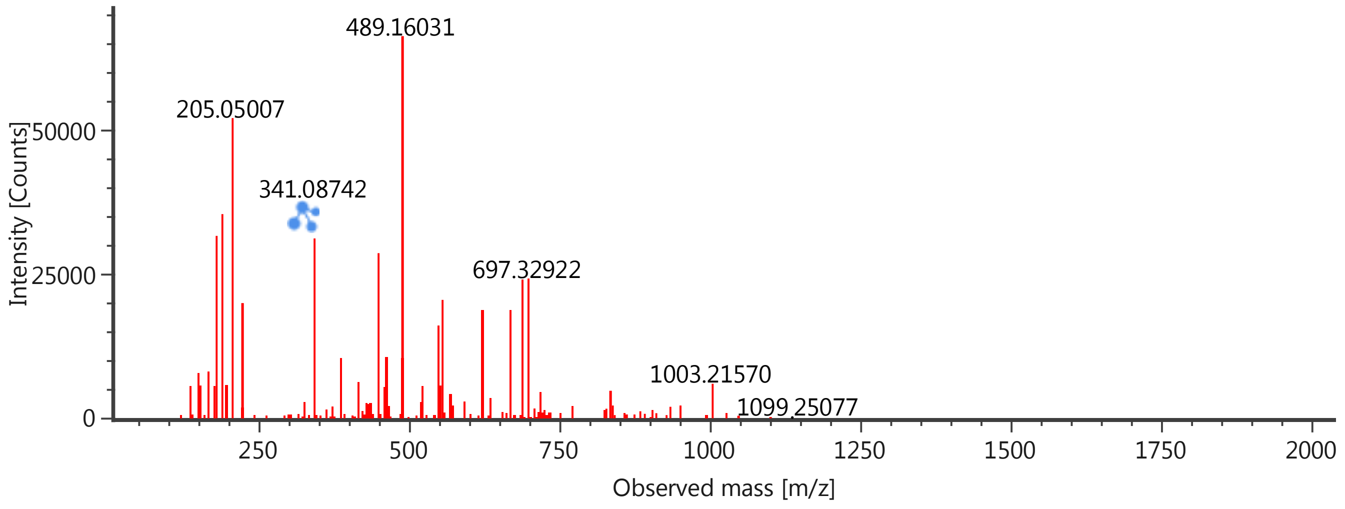

Component name: p-Coumaric acid

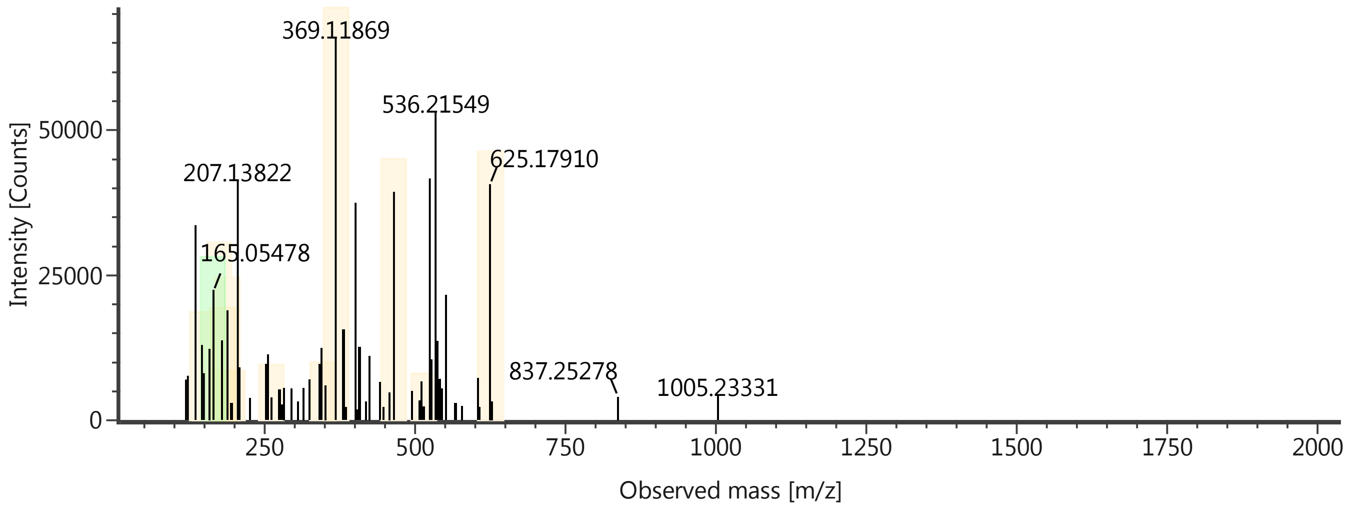

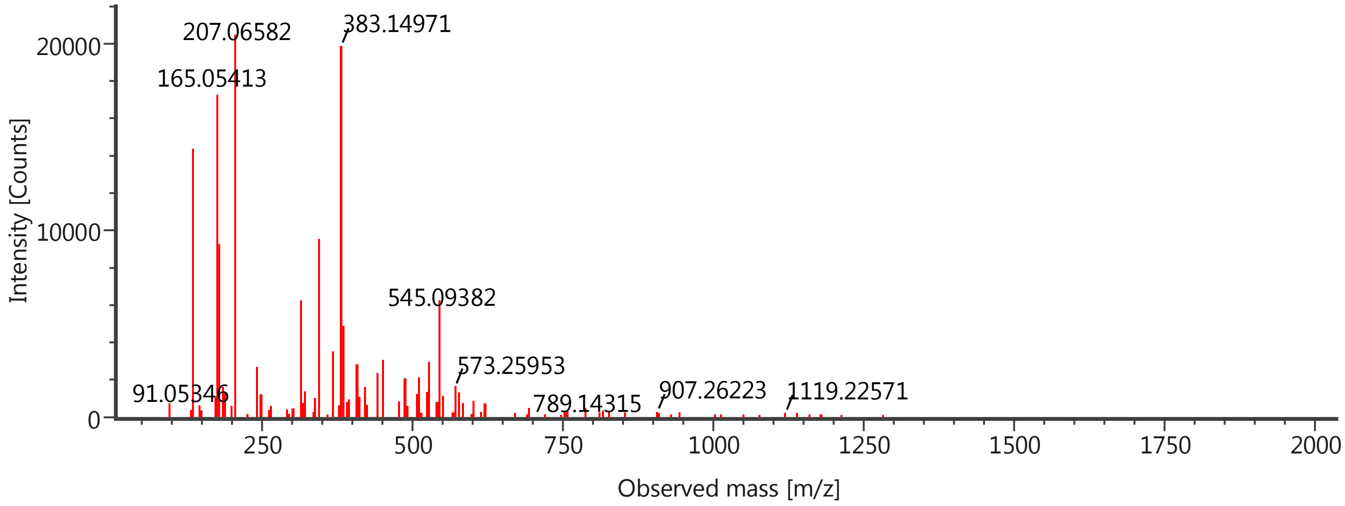

Component name: (1R,3S,4R,5S)-1,3,4-trihydroxy-5-[(E)-3-(4-hydroxypheny

l)prop-2-enoyl]oxy-cyclohexane-1-carboxylicacid

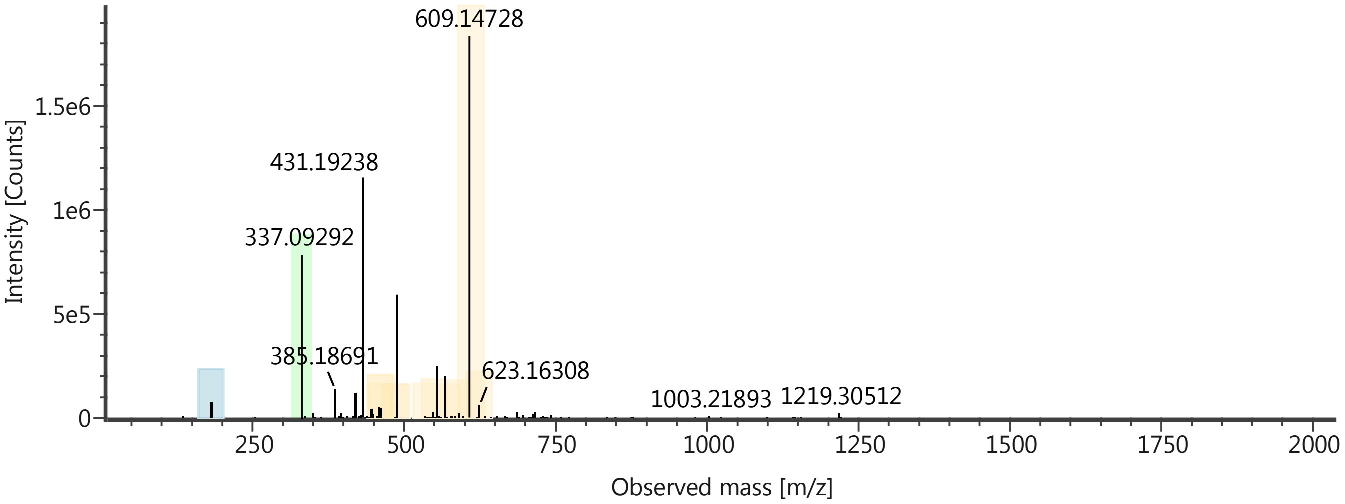

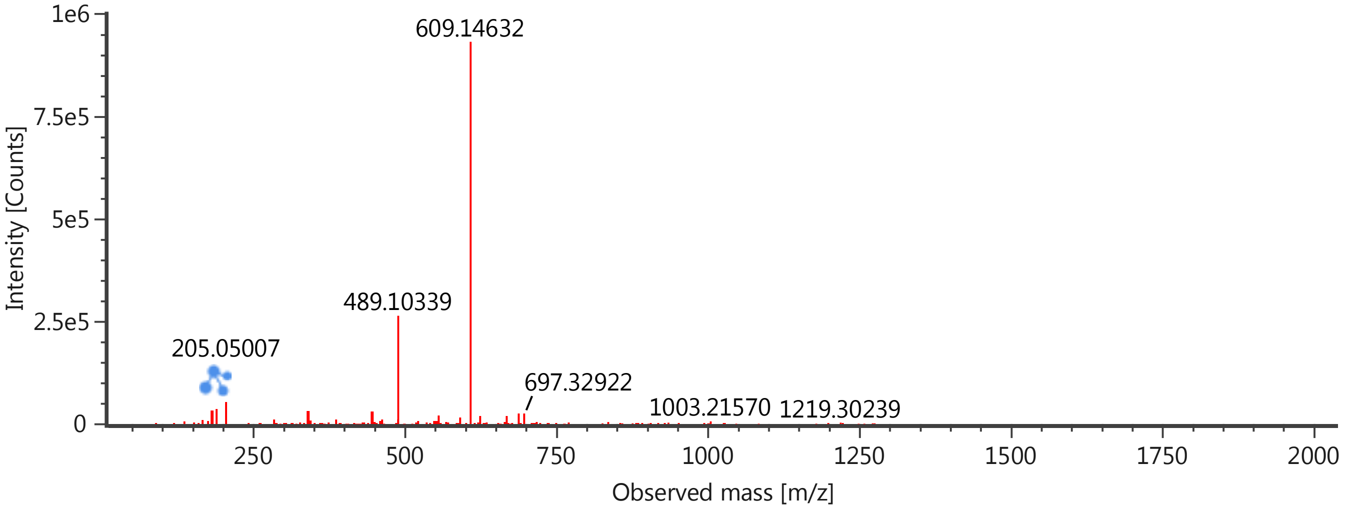

Component name: 2-naphthylamine

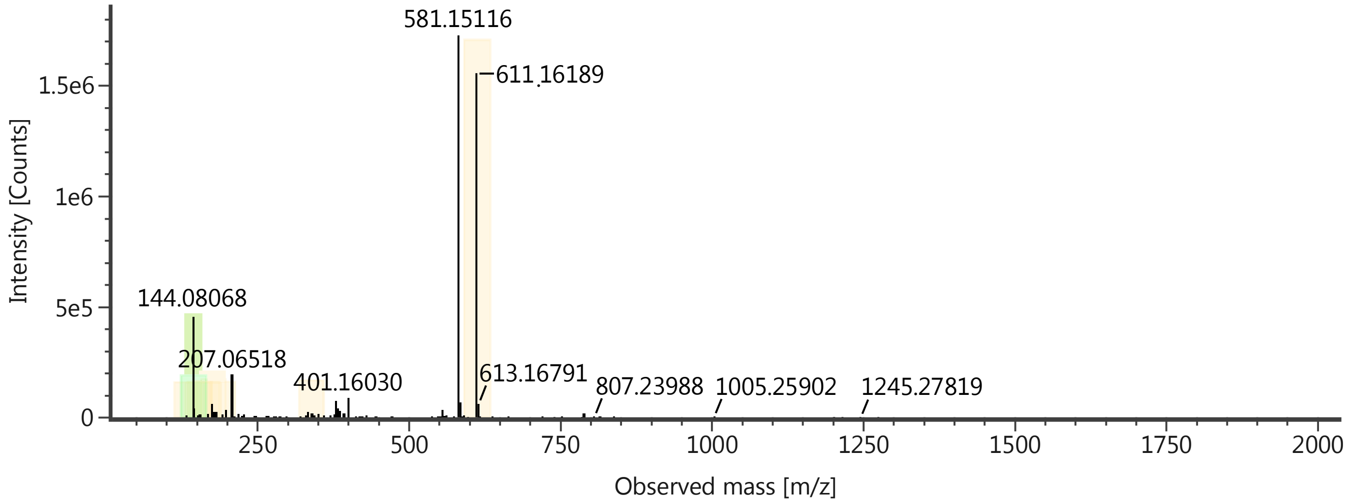

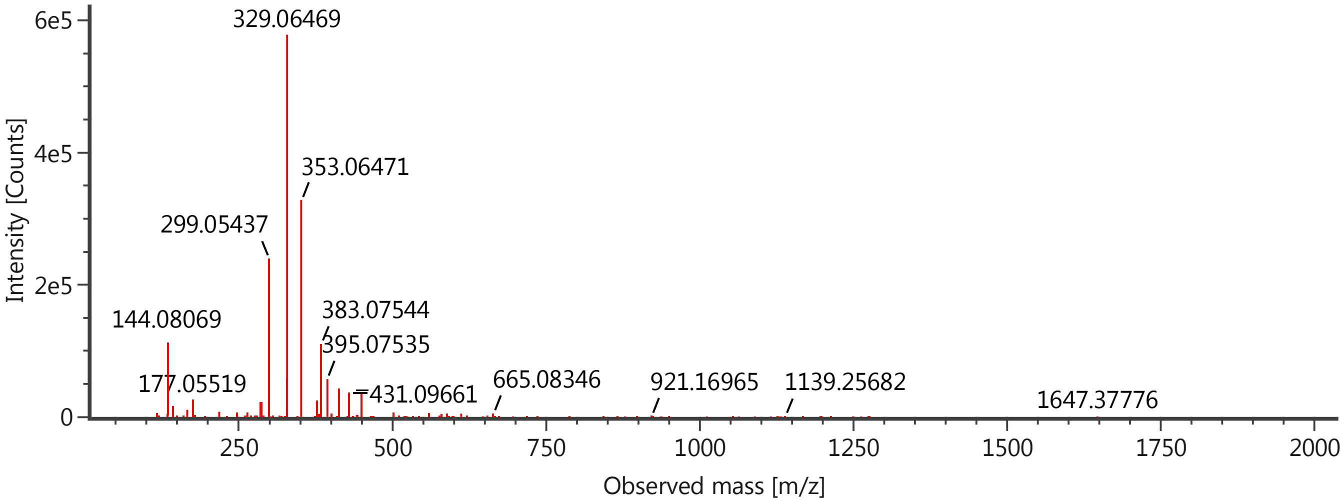

Component name: vanillin

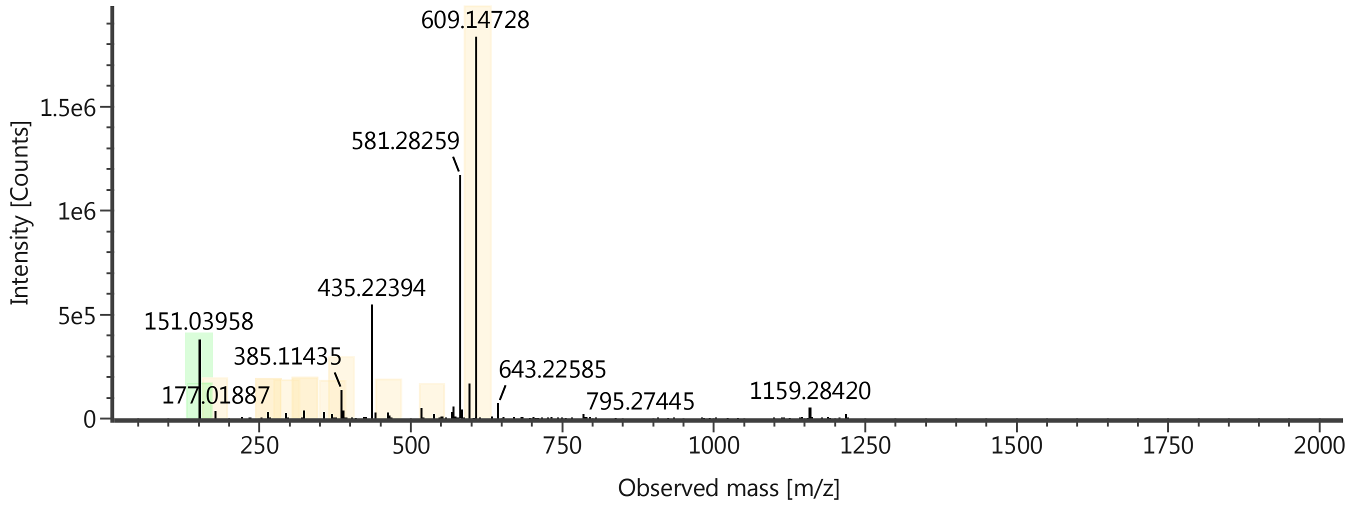

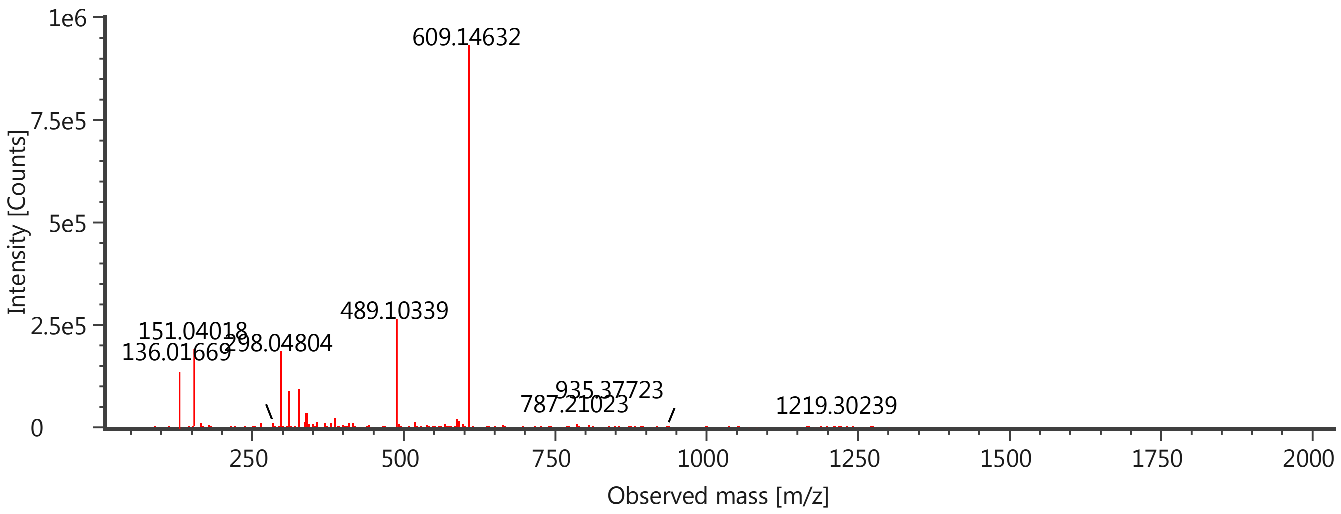

Component name: luteolin

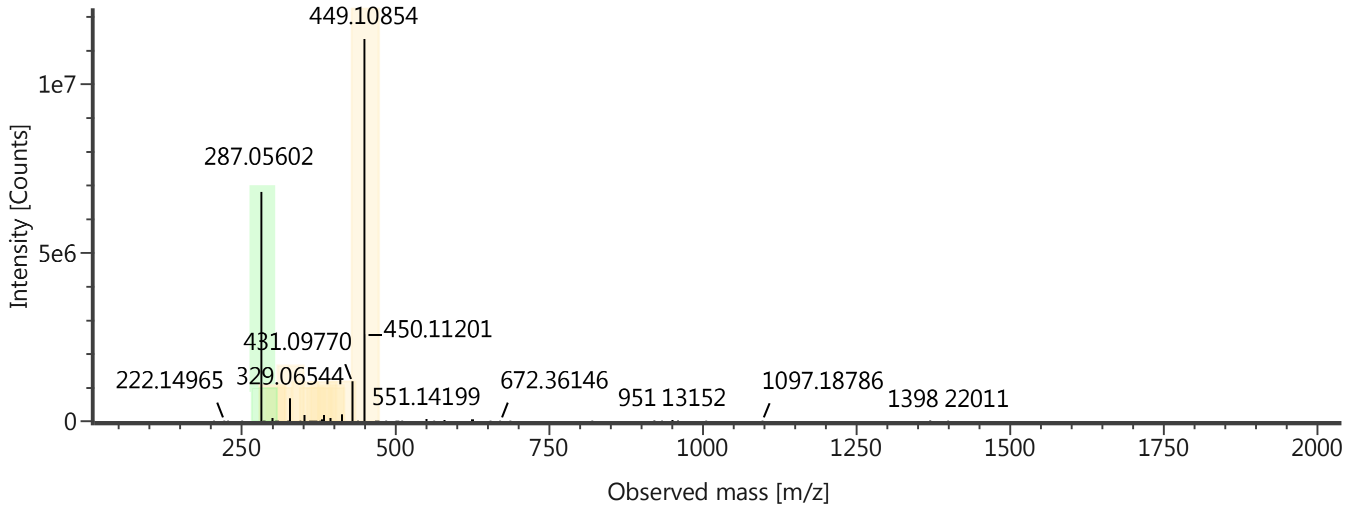

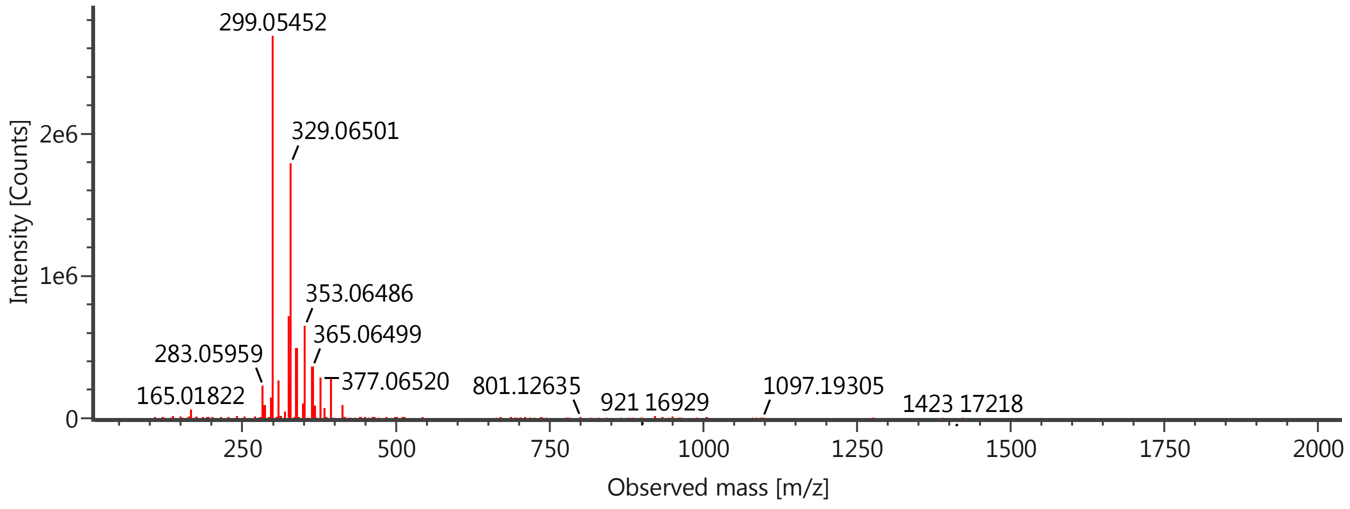

Component name: Orientin

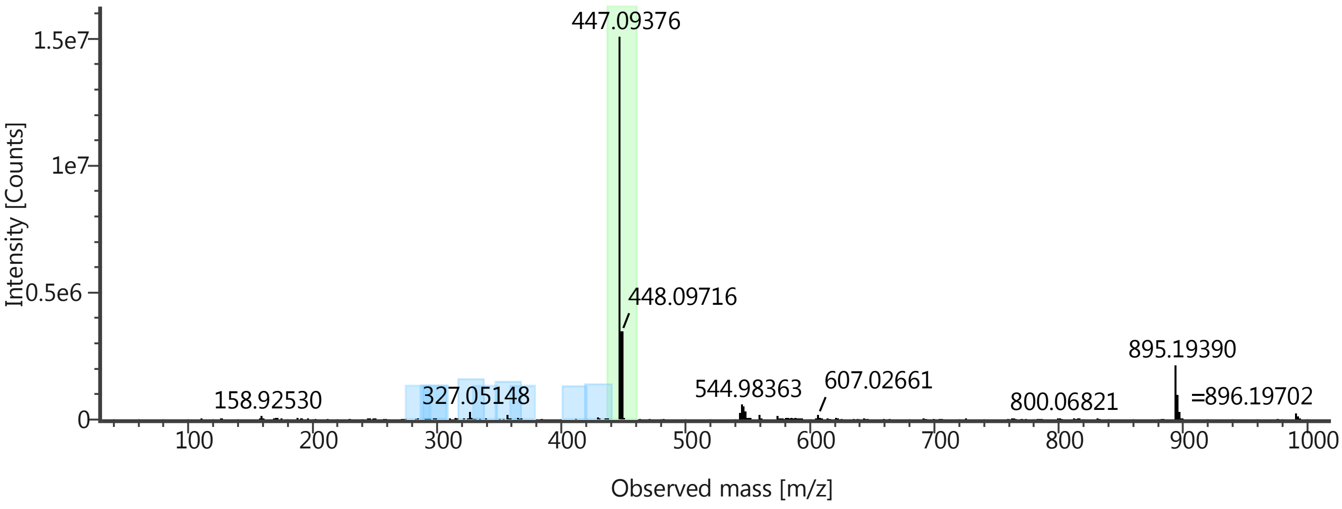

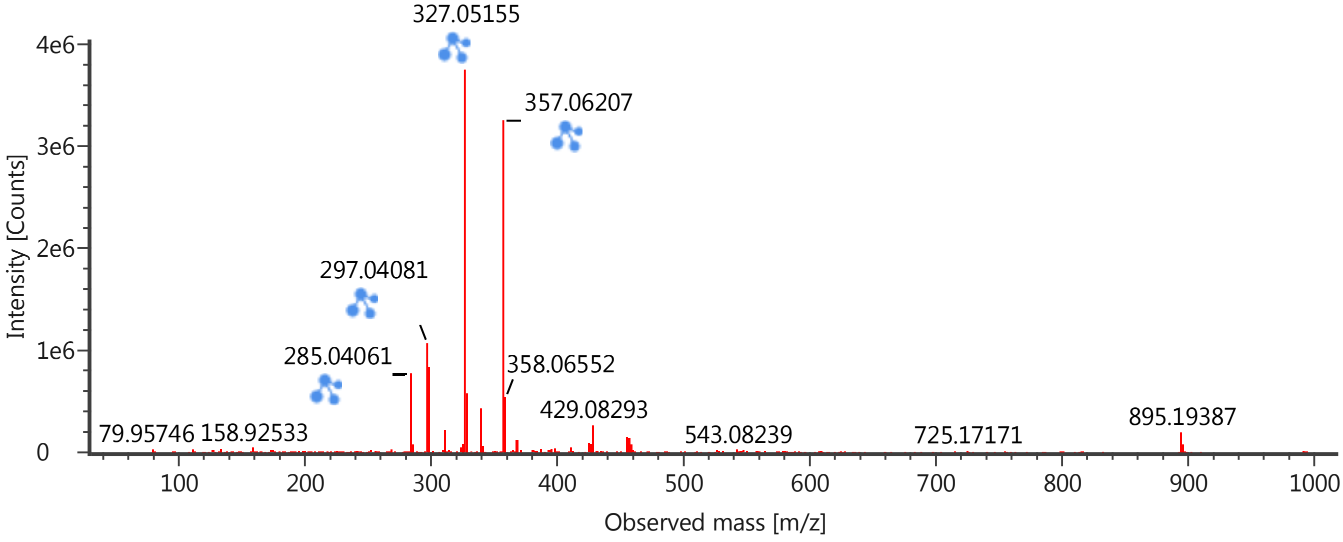

Component name: apigenin 5-O-neohesper

idoside

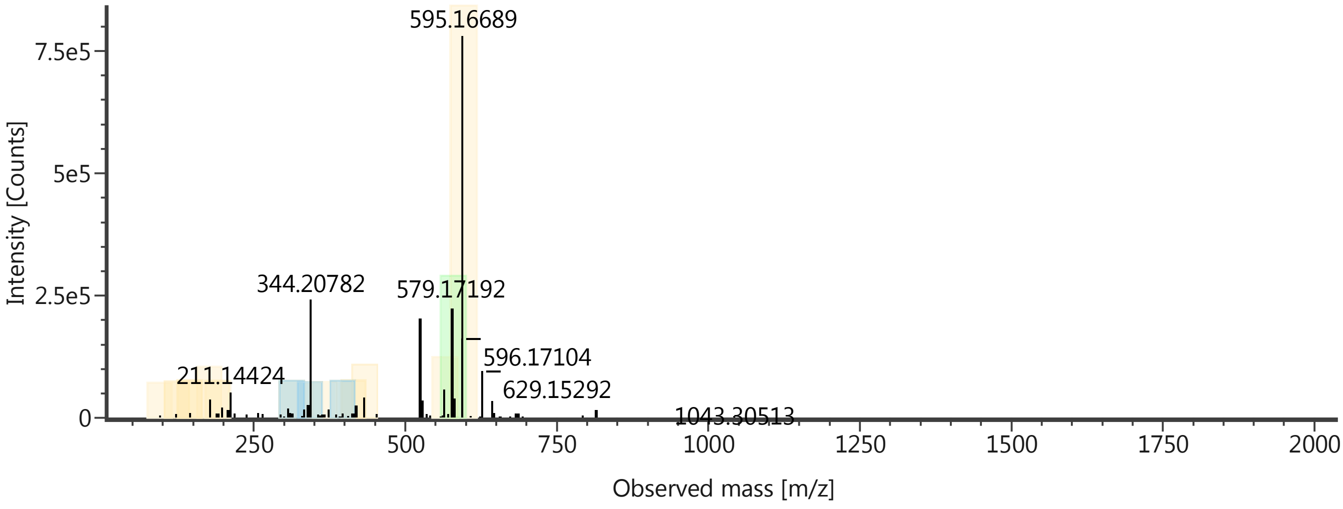

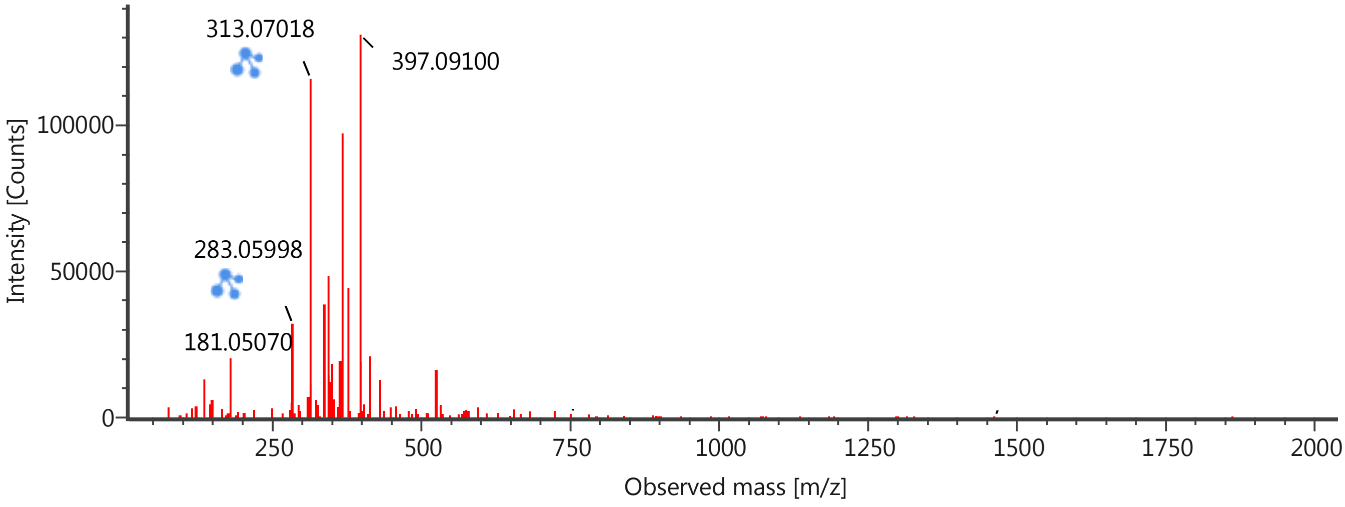

Component name: vitexin-2-O-rhamnoside

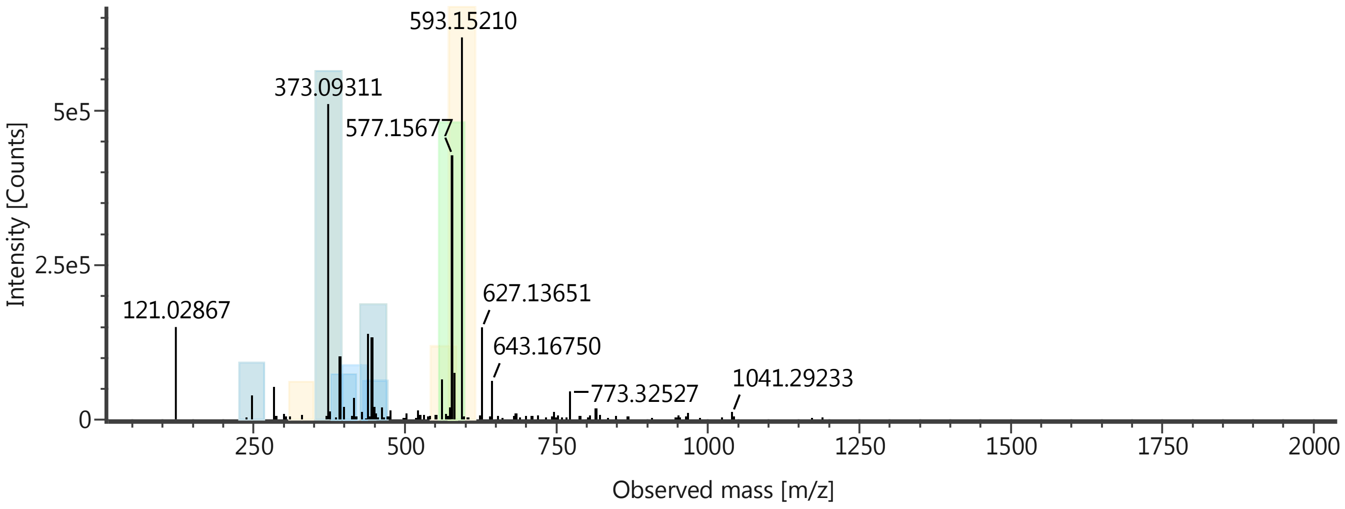

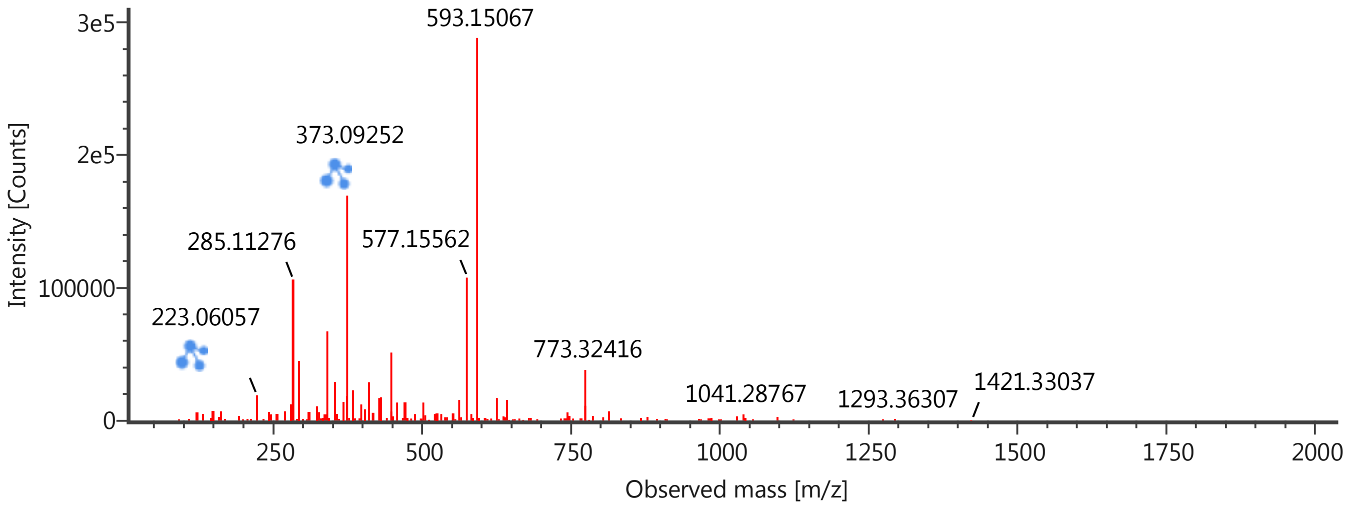

Component name: homoeriodictyol 7-O-gluc

oside

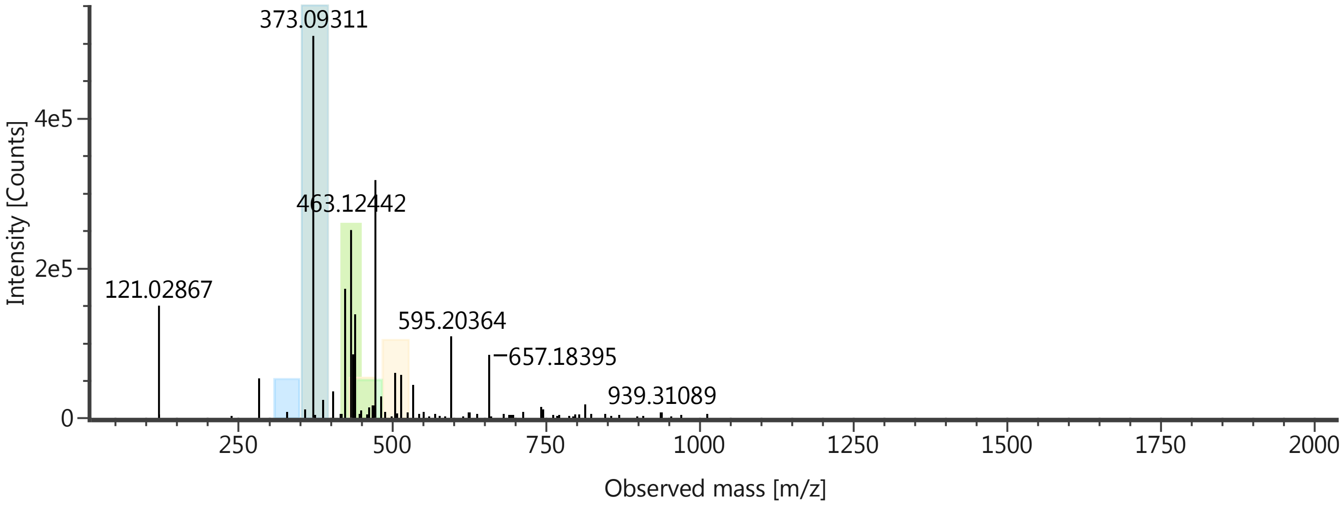

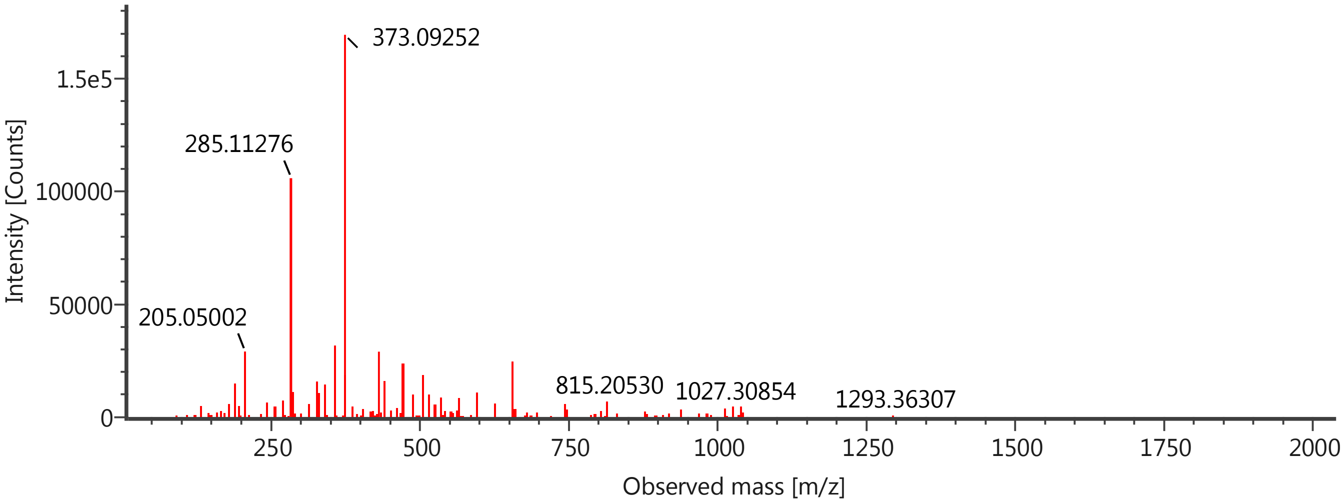

Component name: diosmin

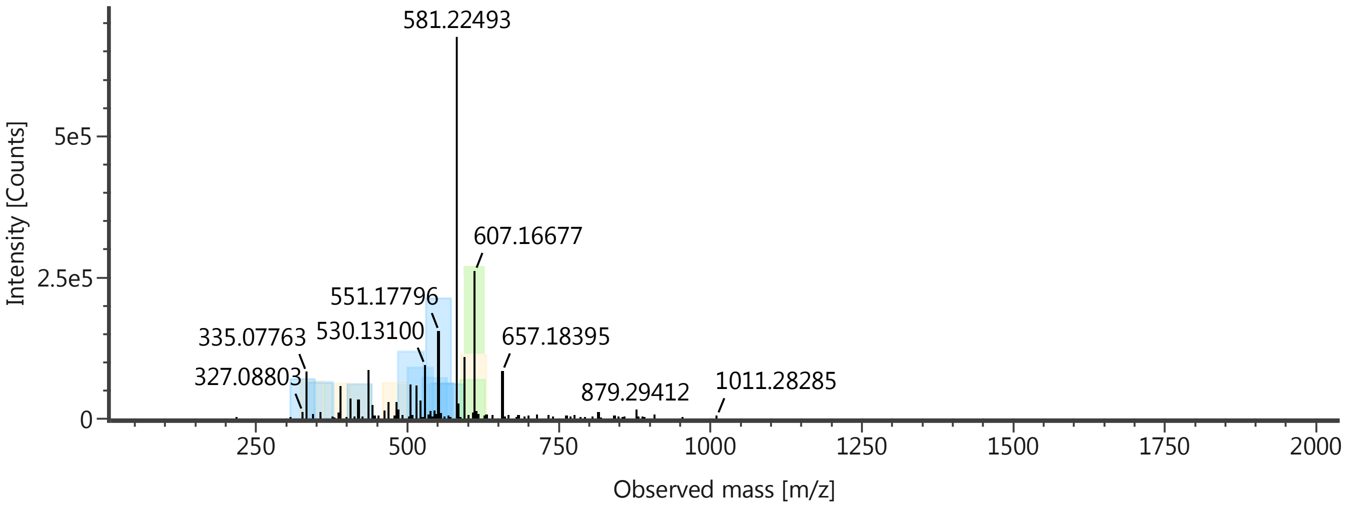

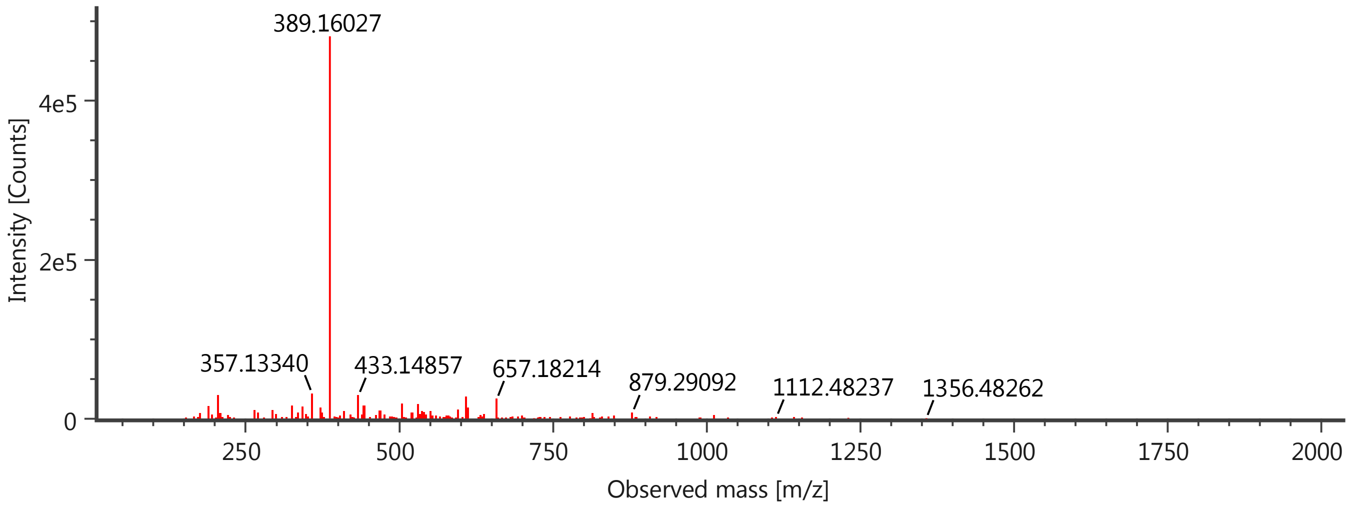

Component name: usnic acid

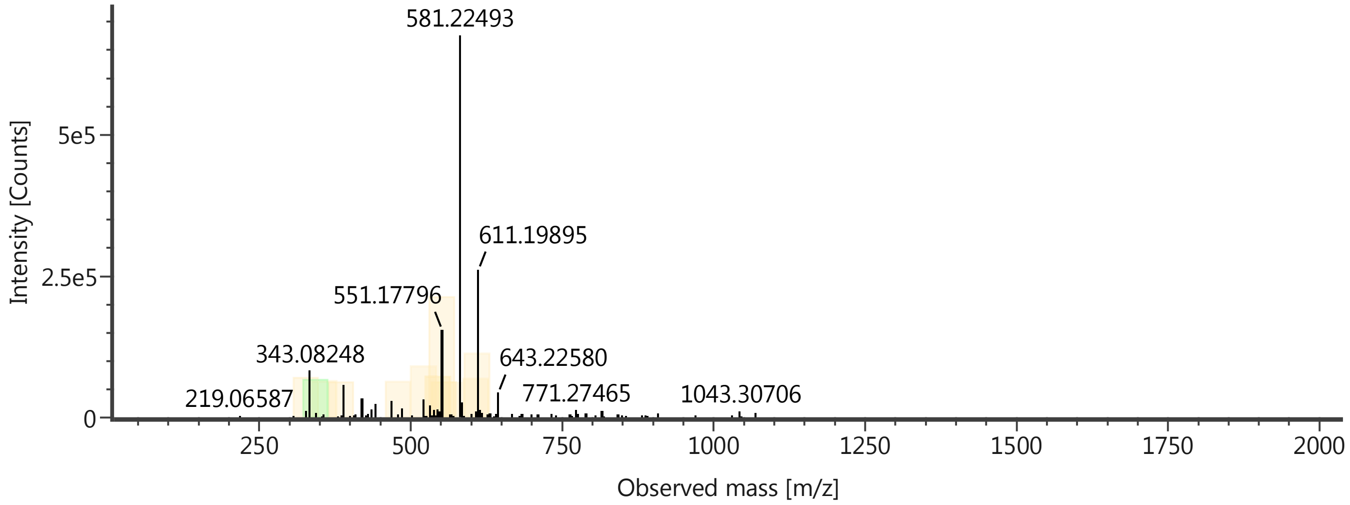

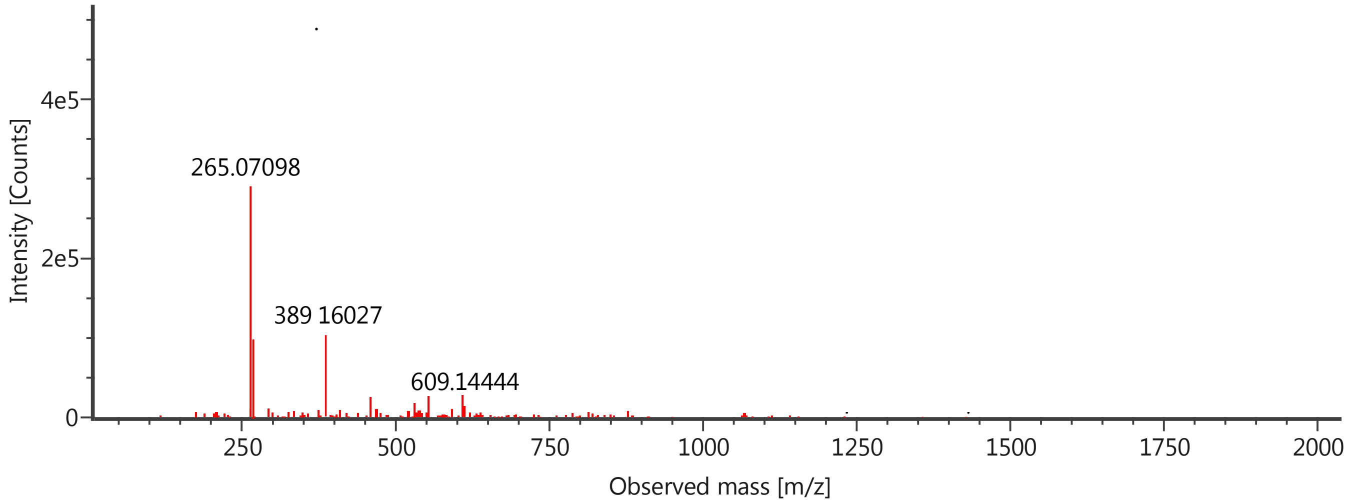

Component name: vitexin

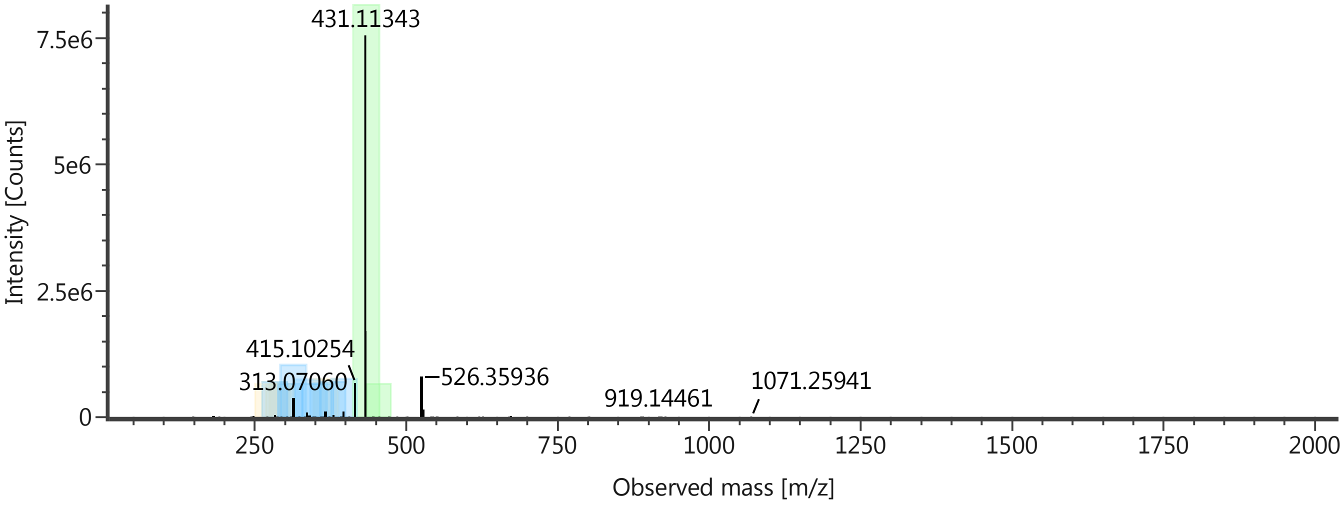

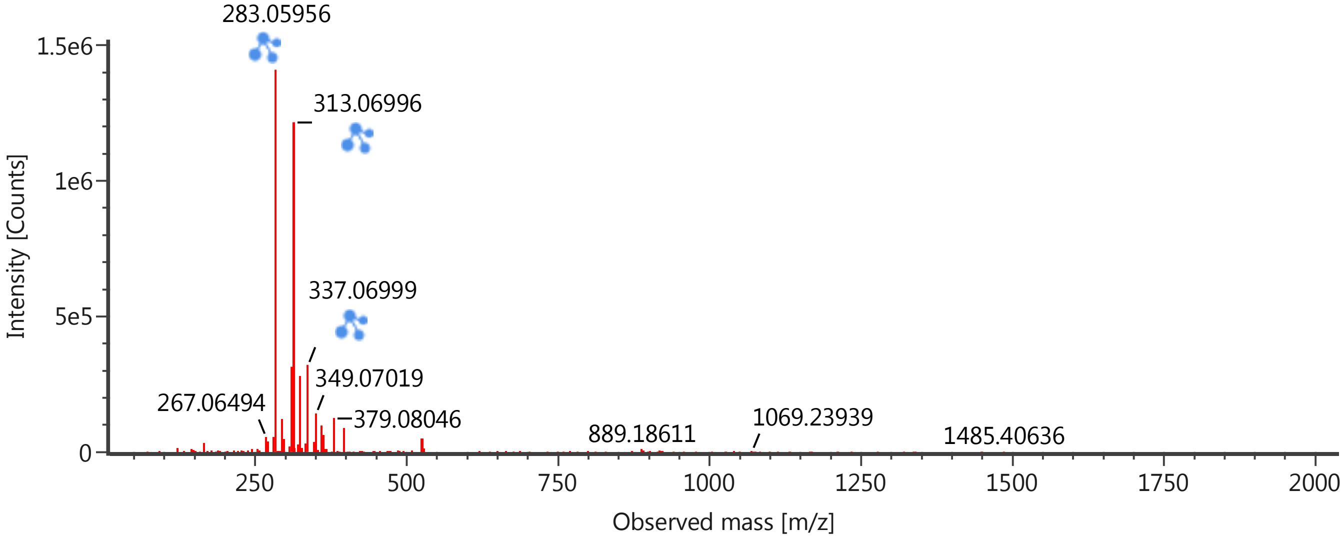

Component name: saponarin

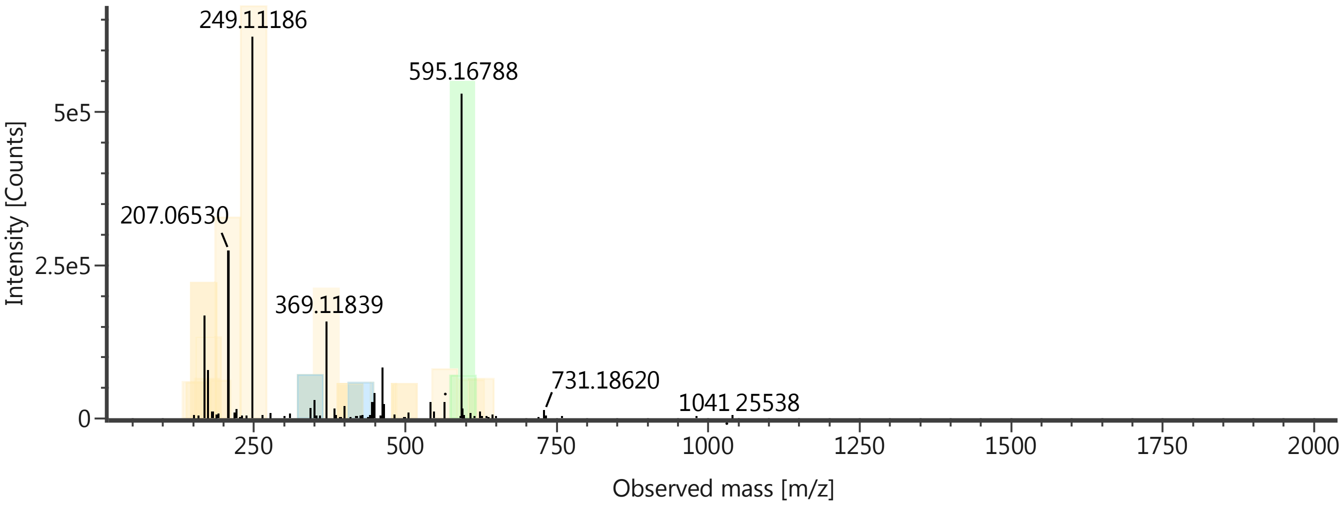

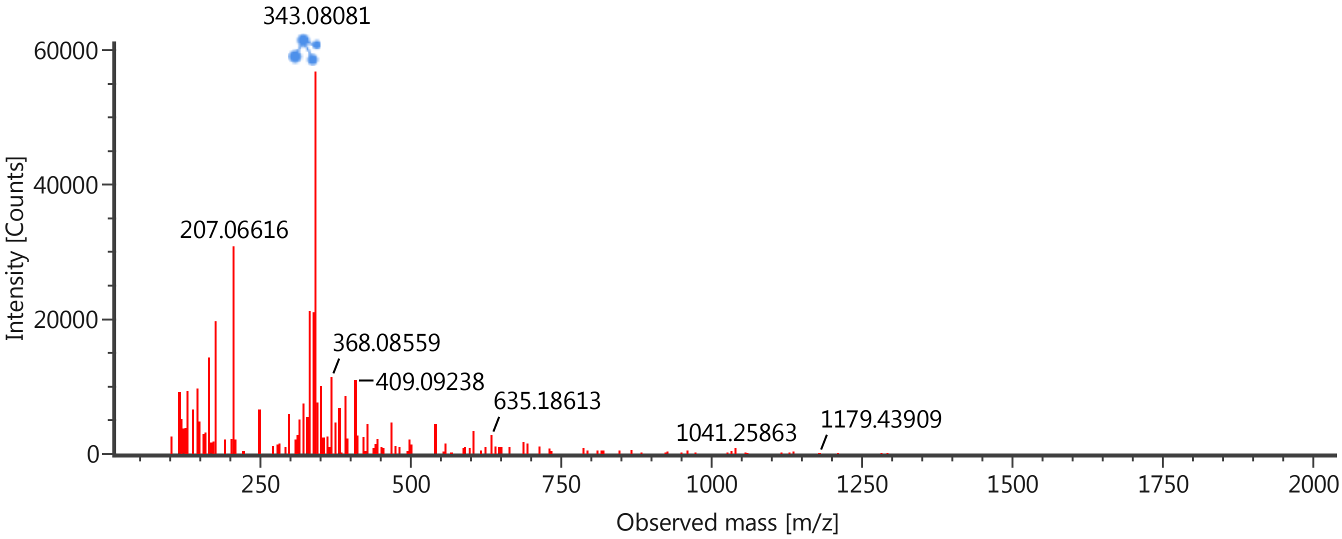

Component name: coumarin

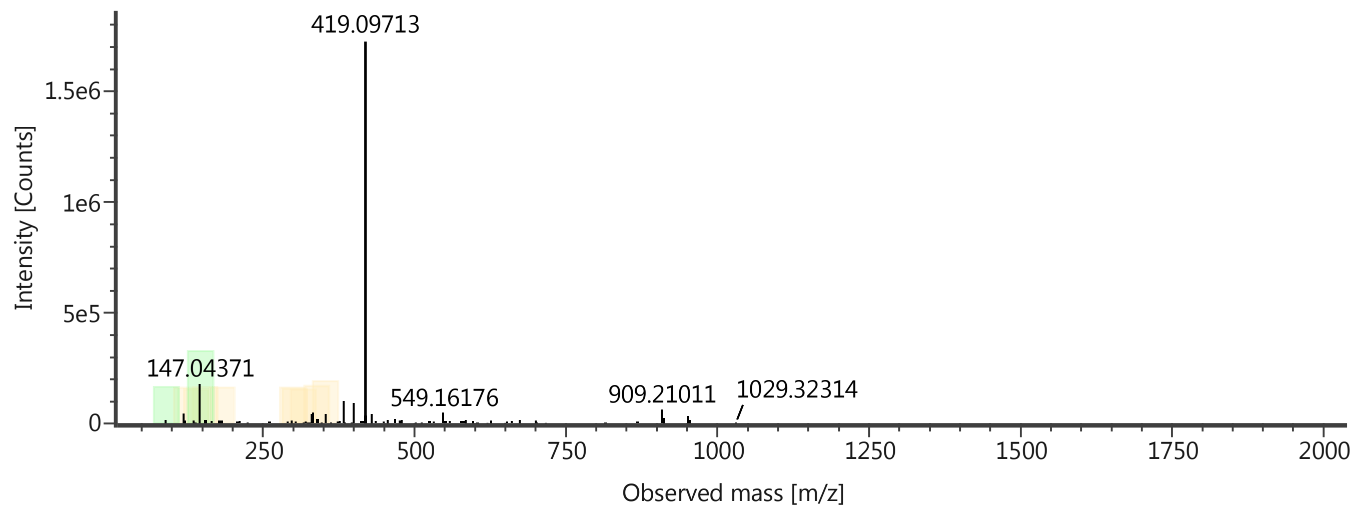

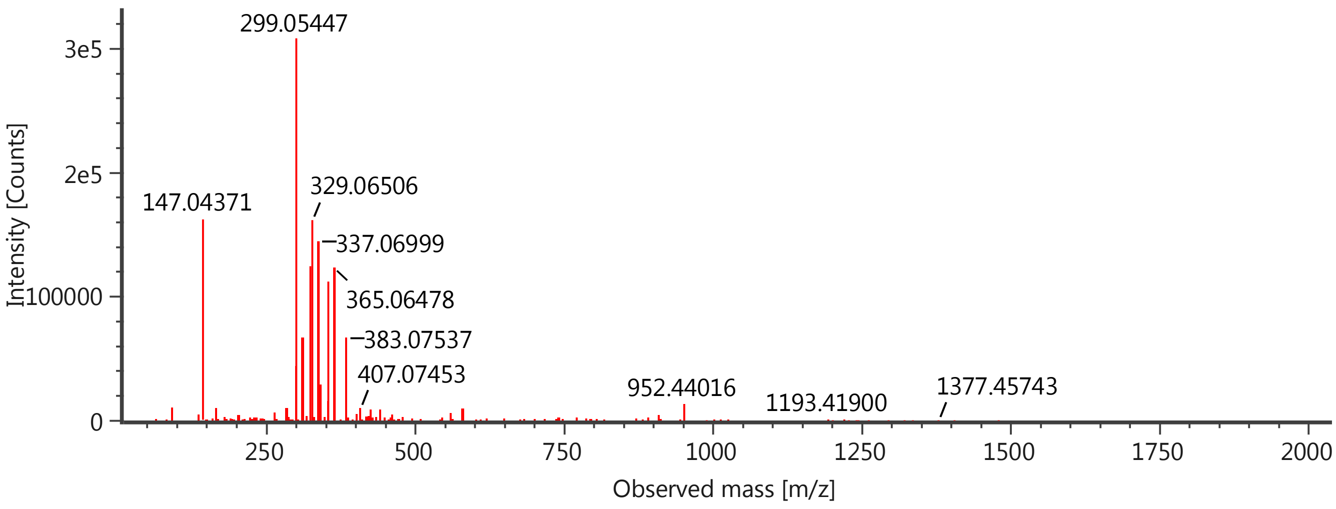

Component name: vanillyl alcohol

Item name: ZYHT
Item description:

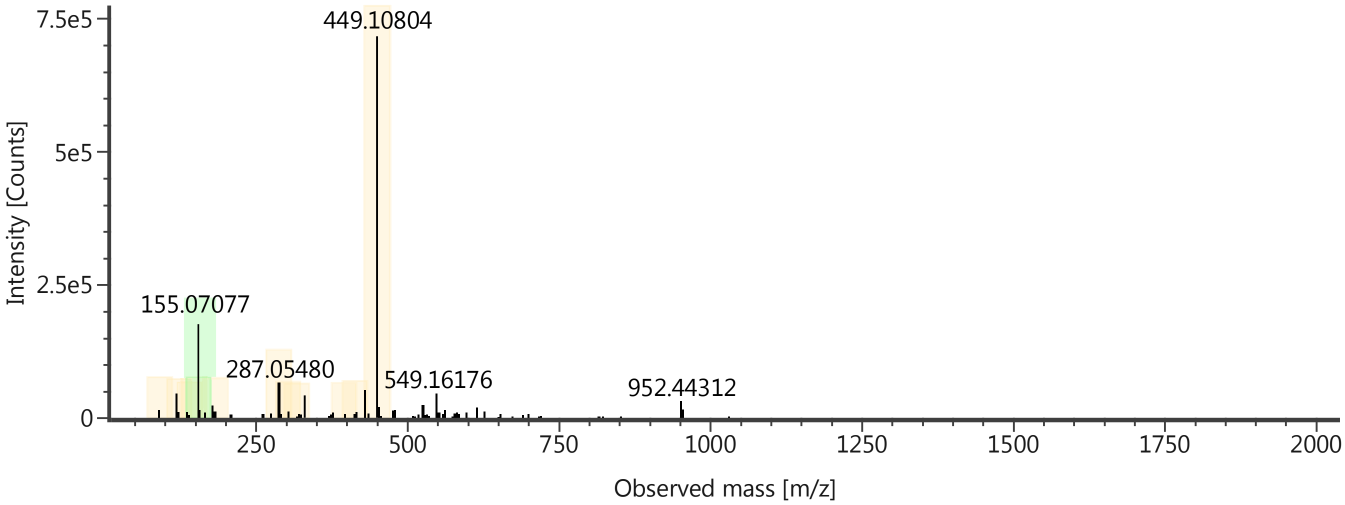

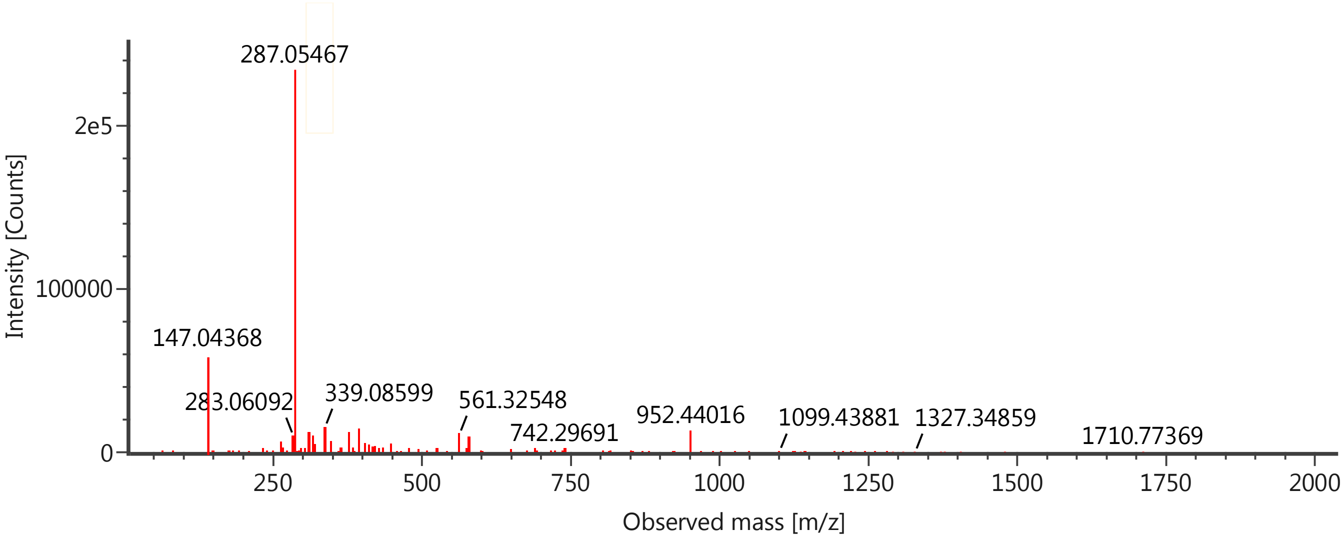

Component name: eriodictyol

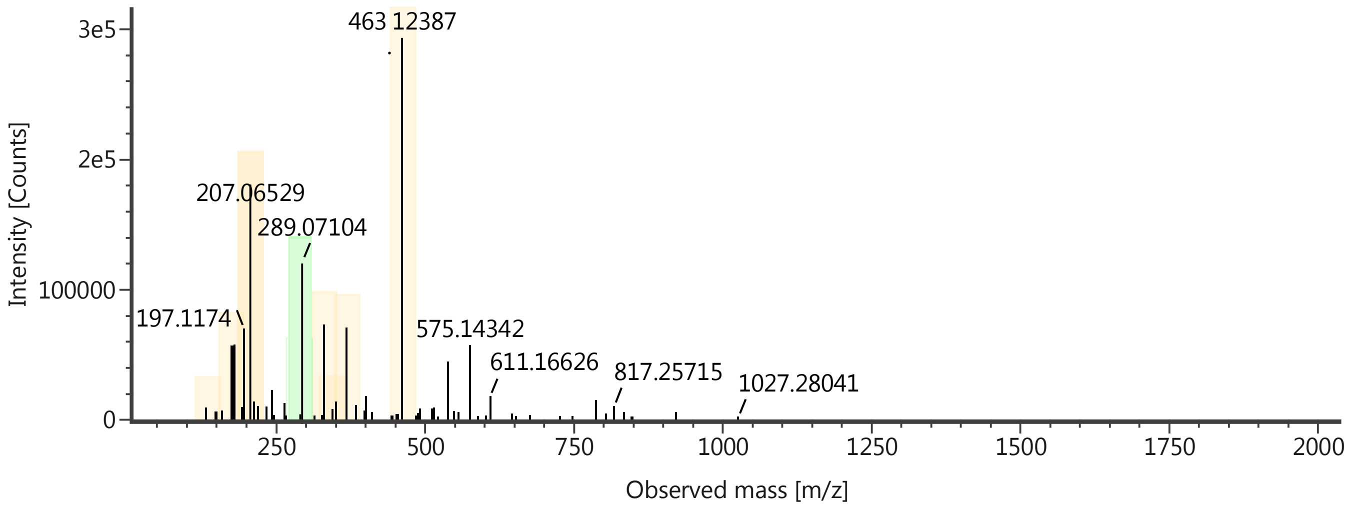

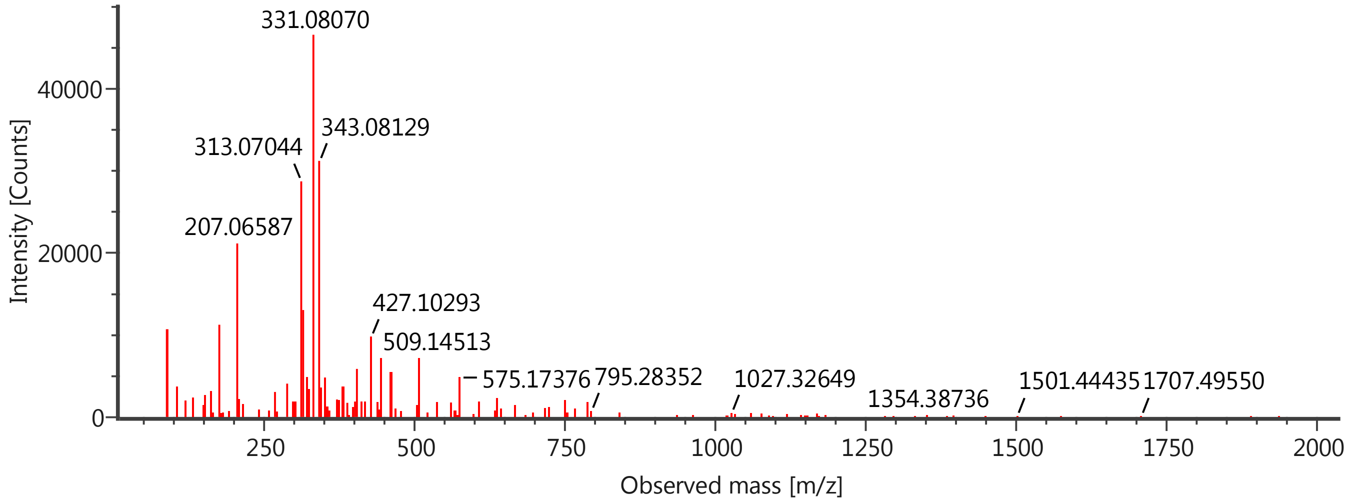

Component name:abietic acid

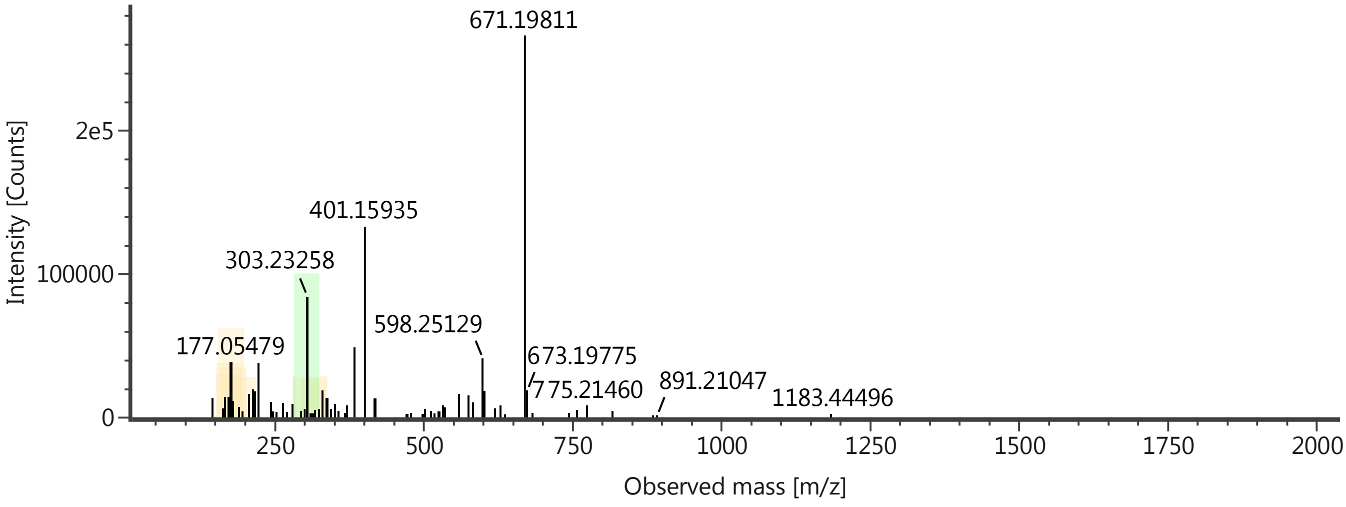

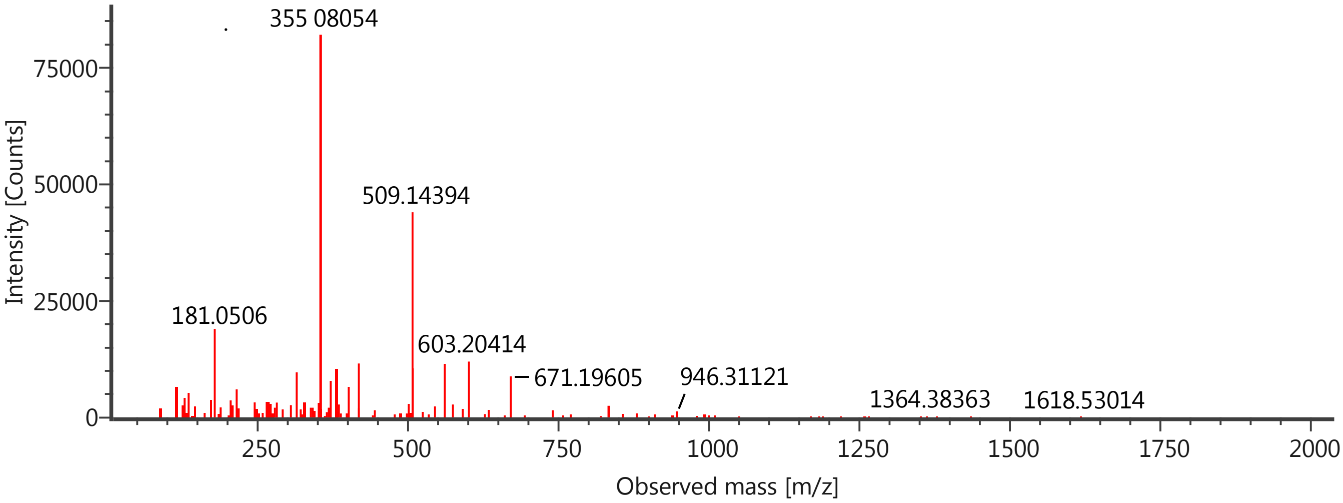

5

Component name:3-O-feruloylquinic acid

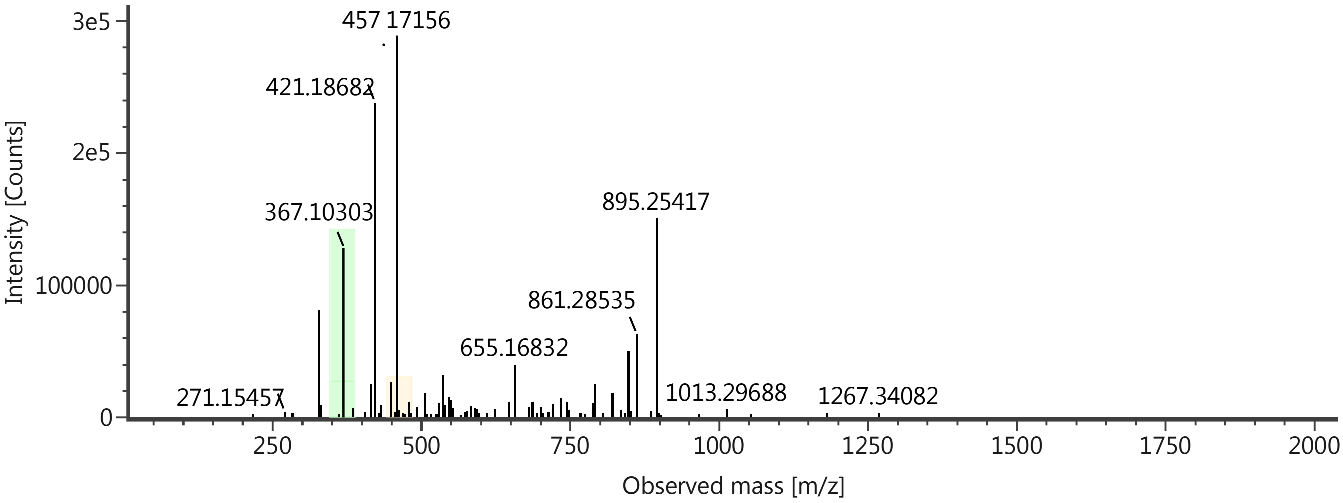

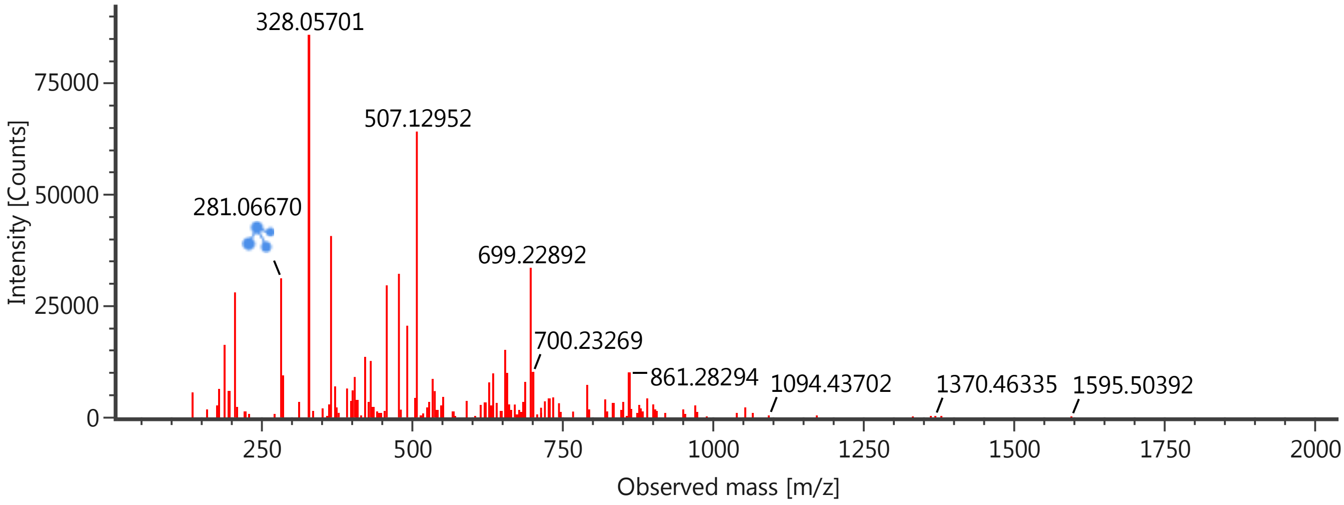

Component name: luteoloside

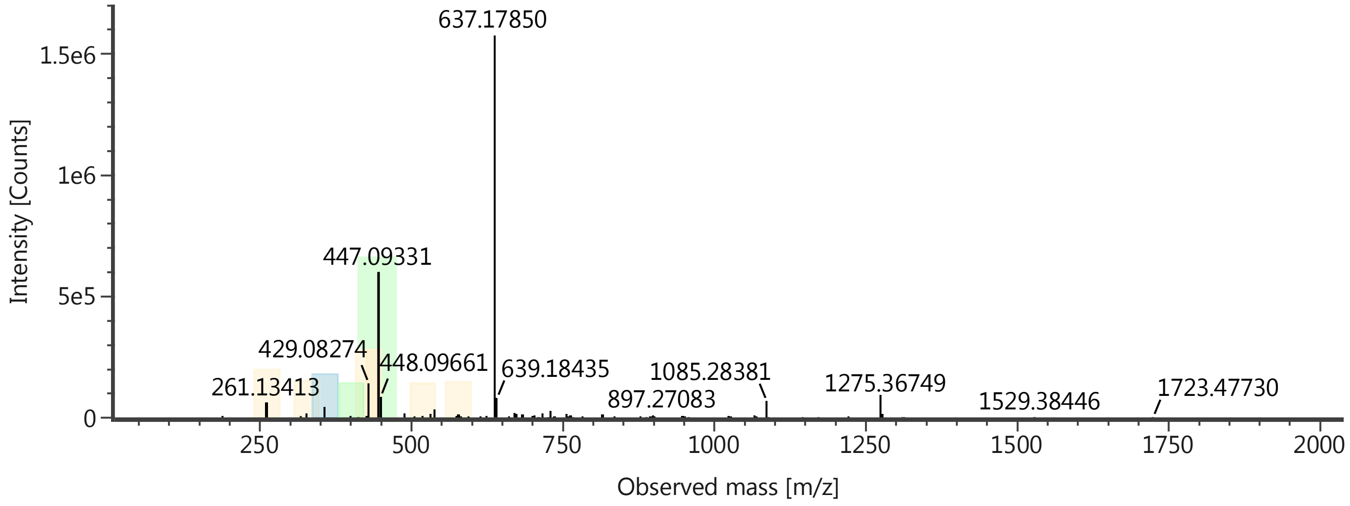

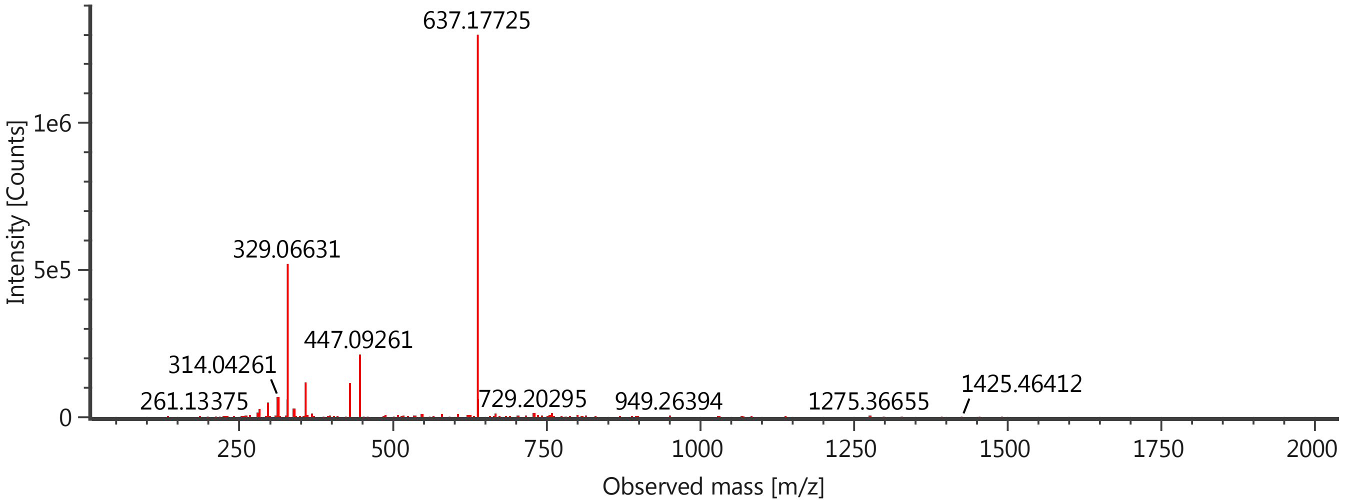

Component name:5,7,4'-trihydroxy-6-meth

oxy isoflavone

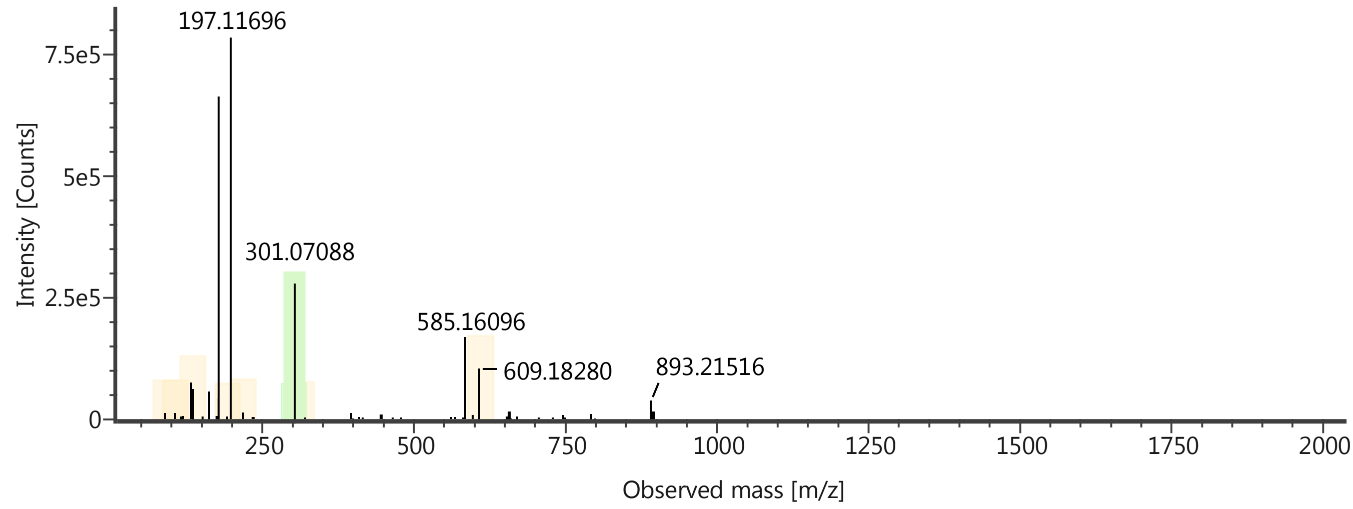

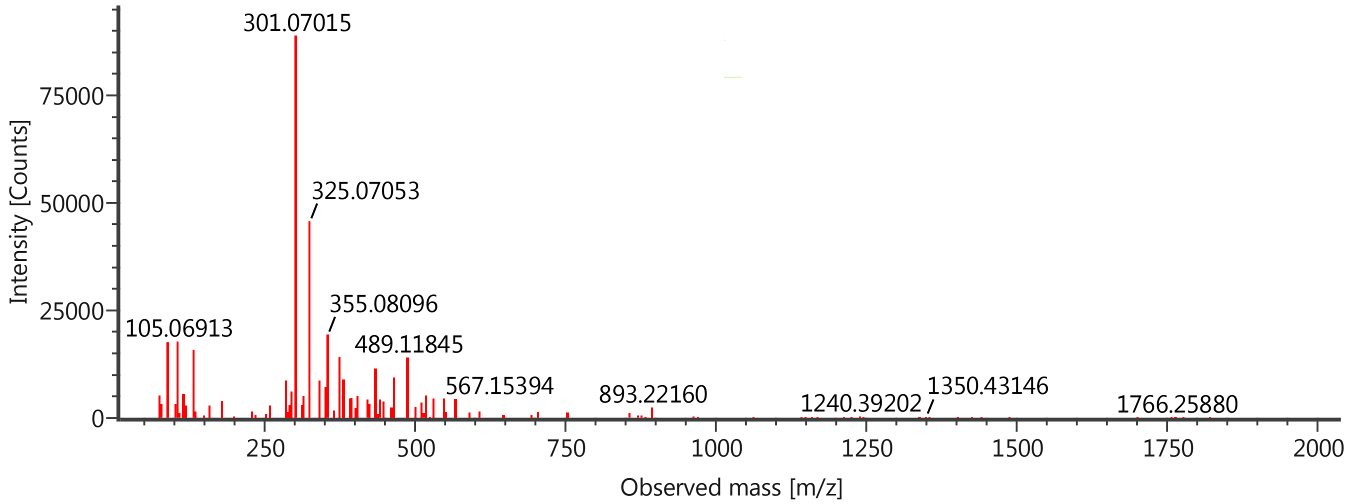

Component name: Isovitexin

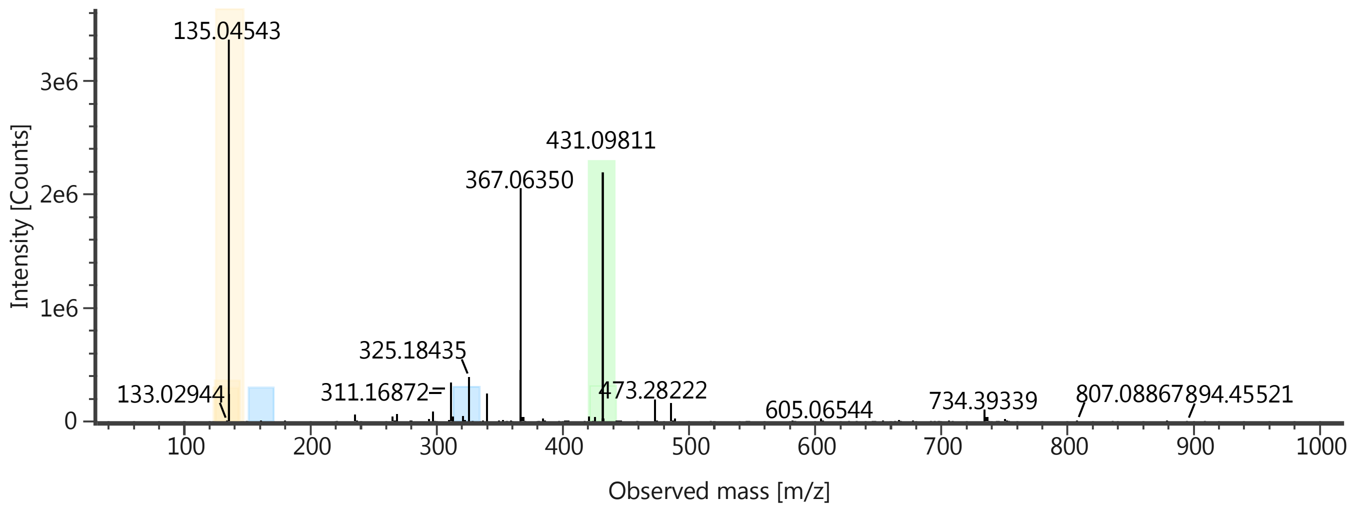

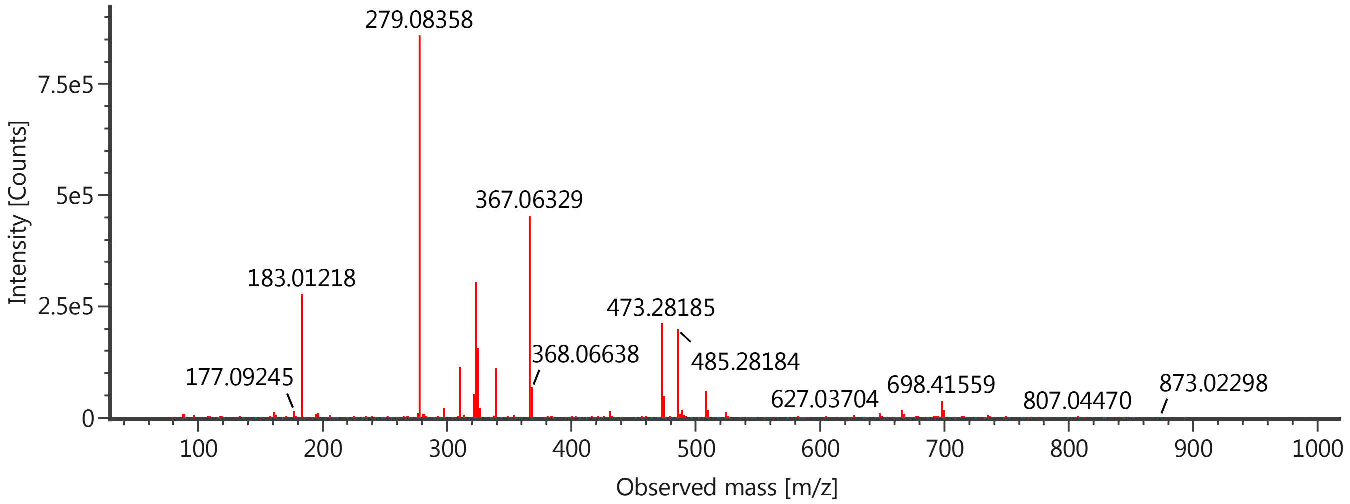

Component name:Tricin

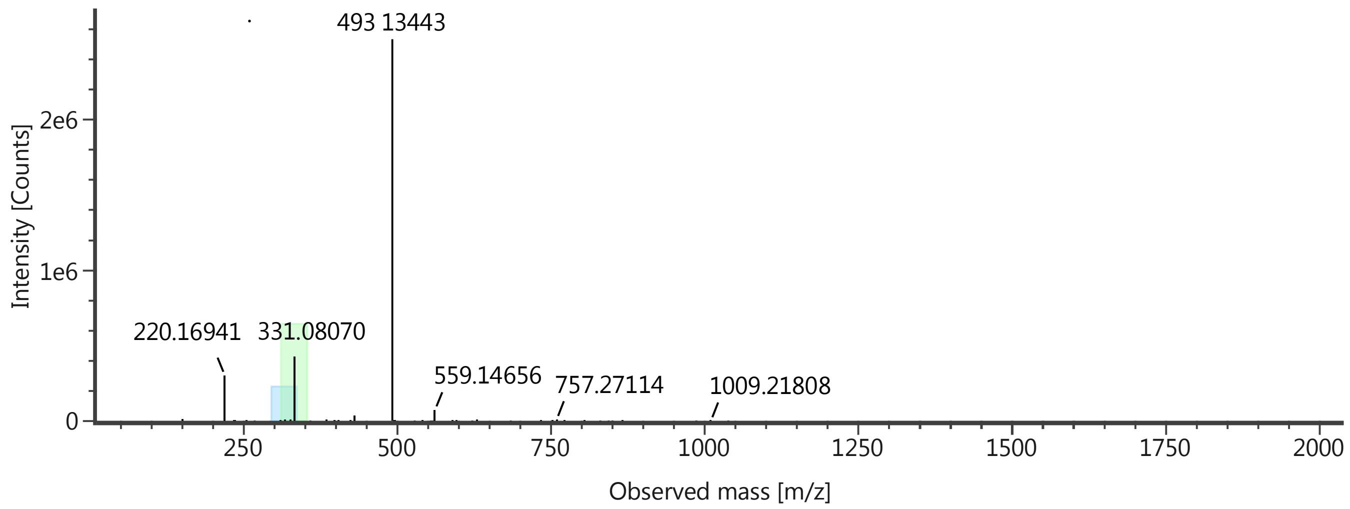

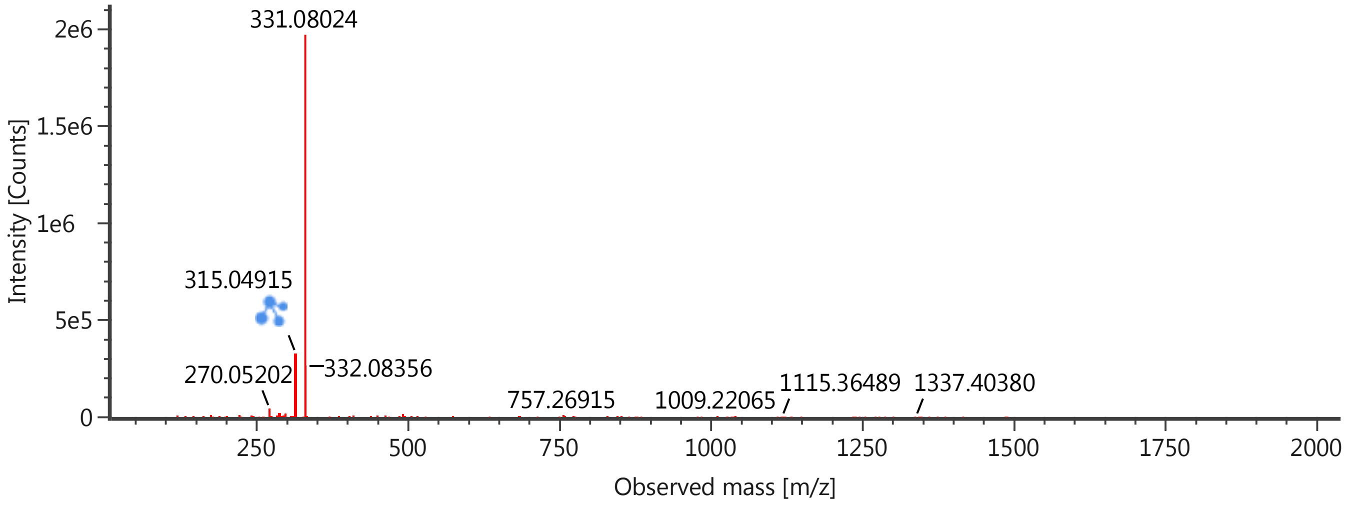

Component name:luteolin-4'-o-glucoside

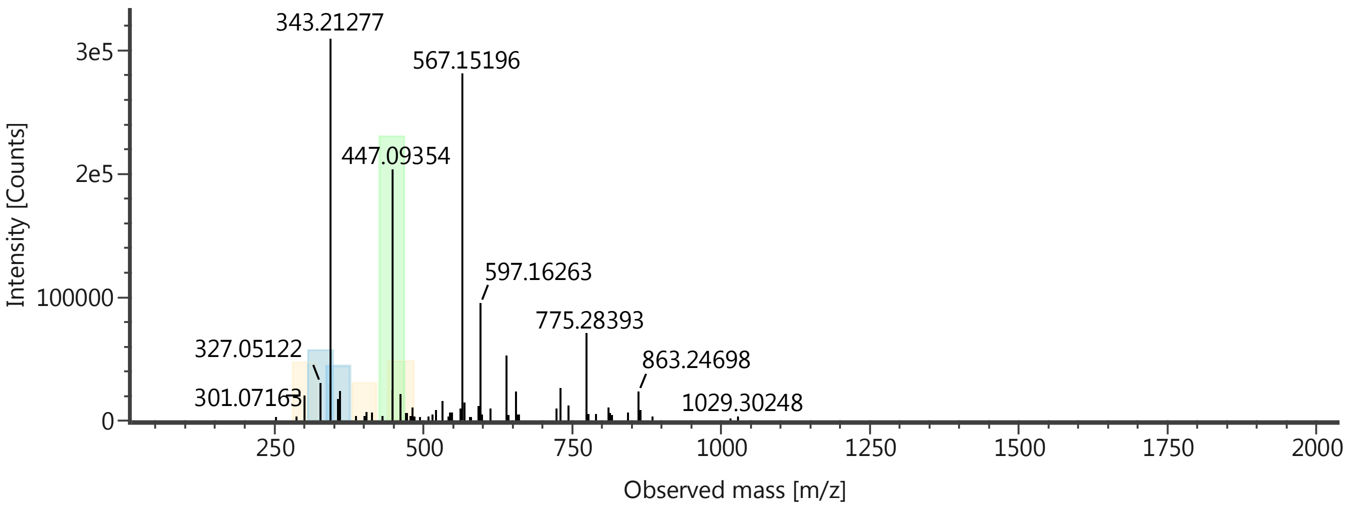

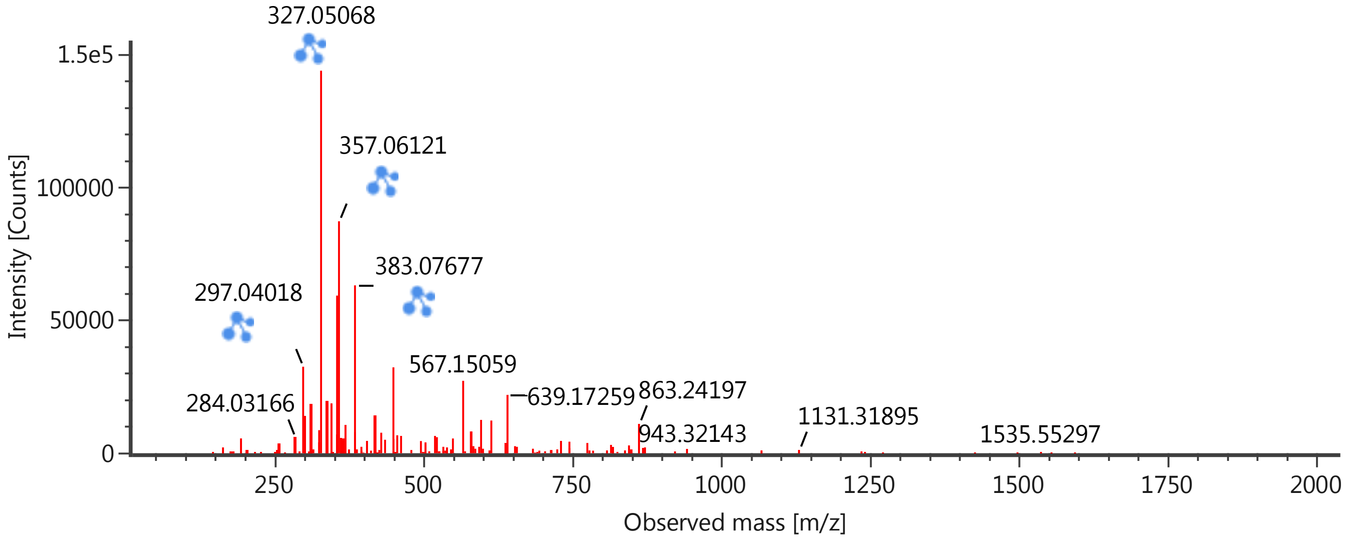

Component name:swertiajaponin

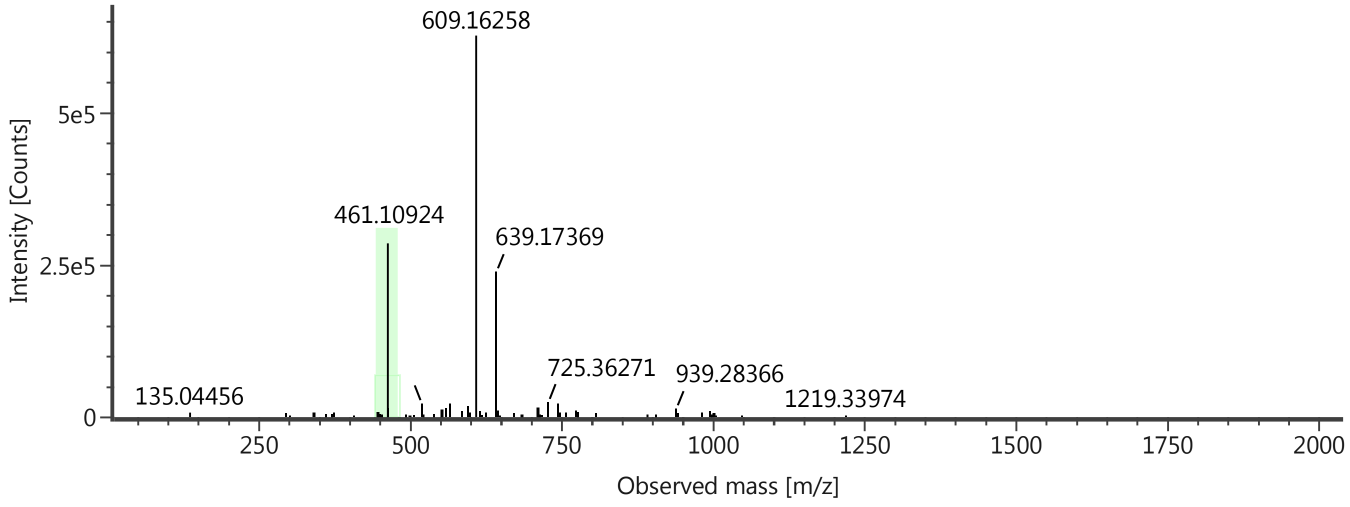

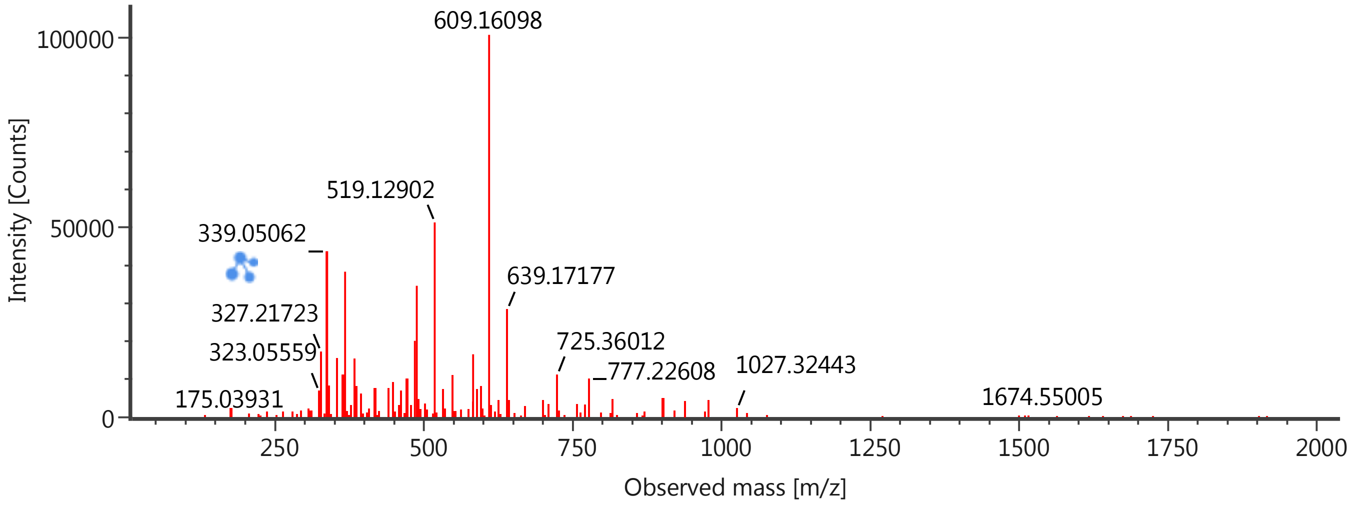

Component name:kaempferol

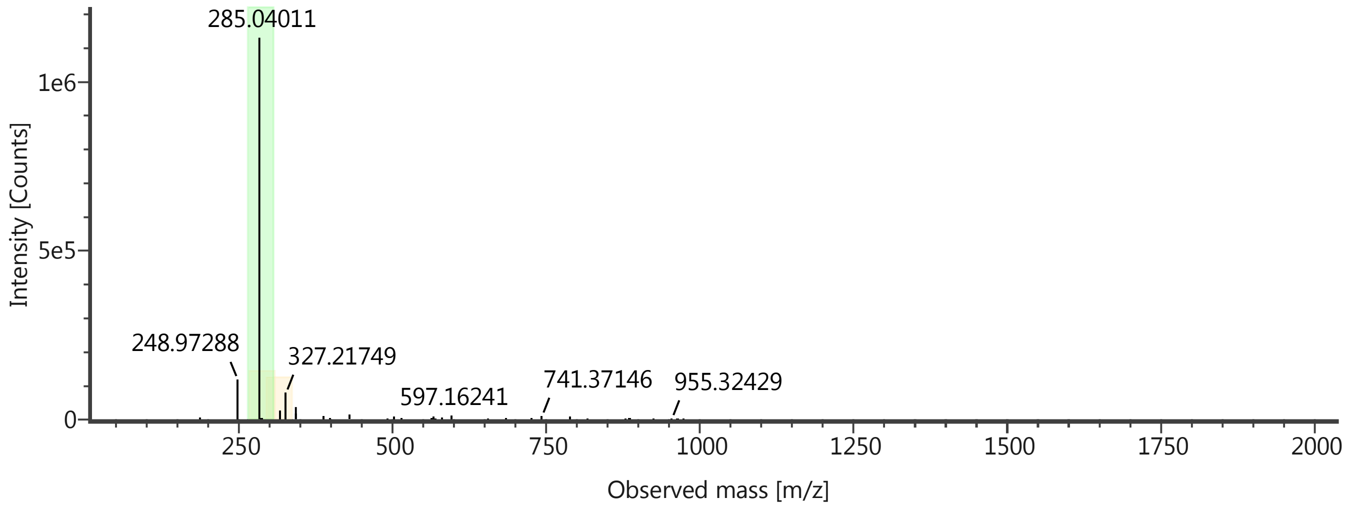

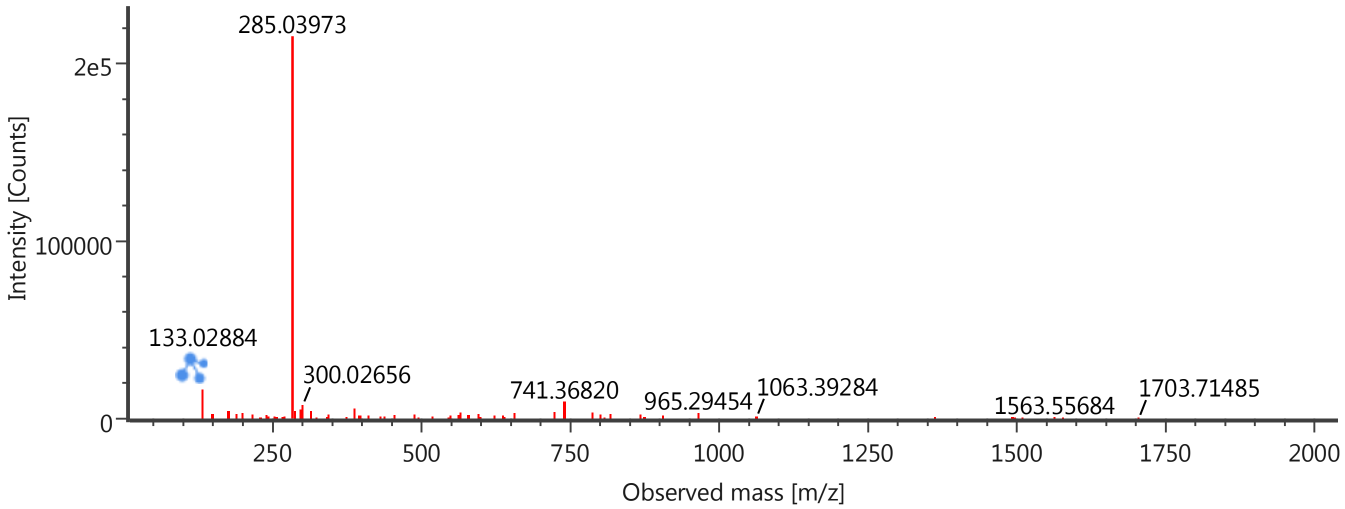

Component name:nobiletin

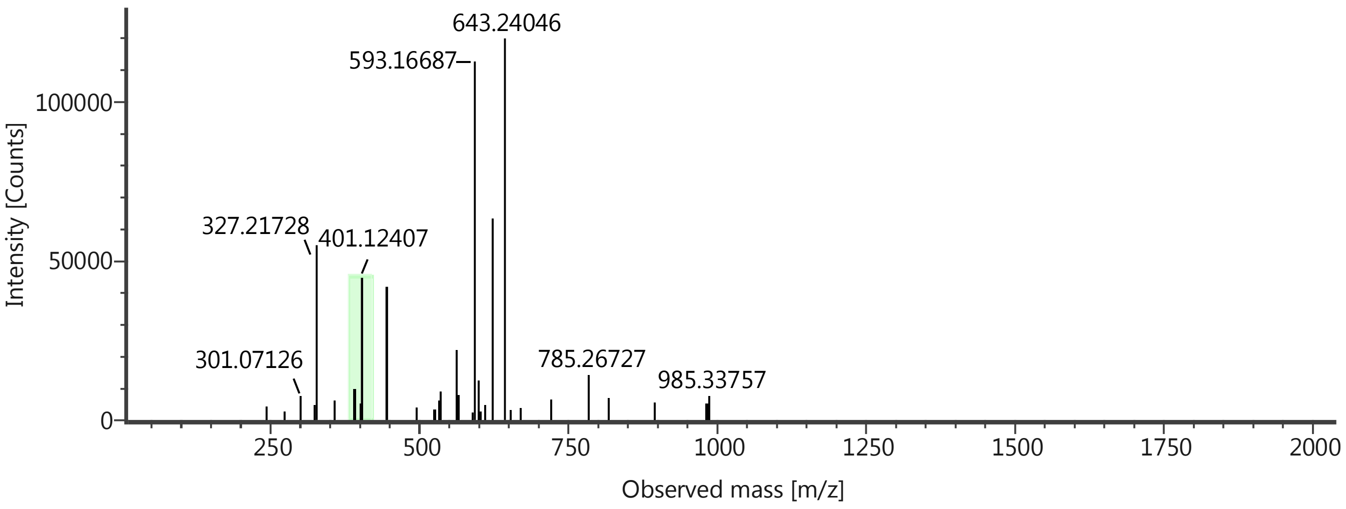

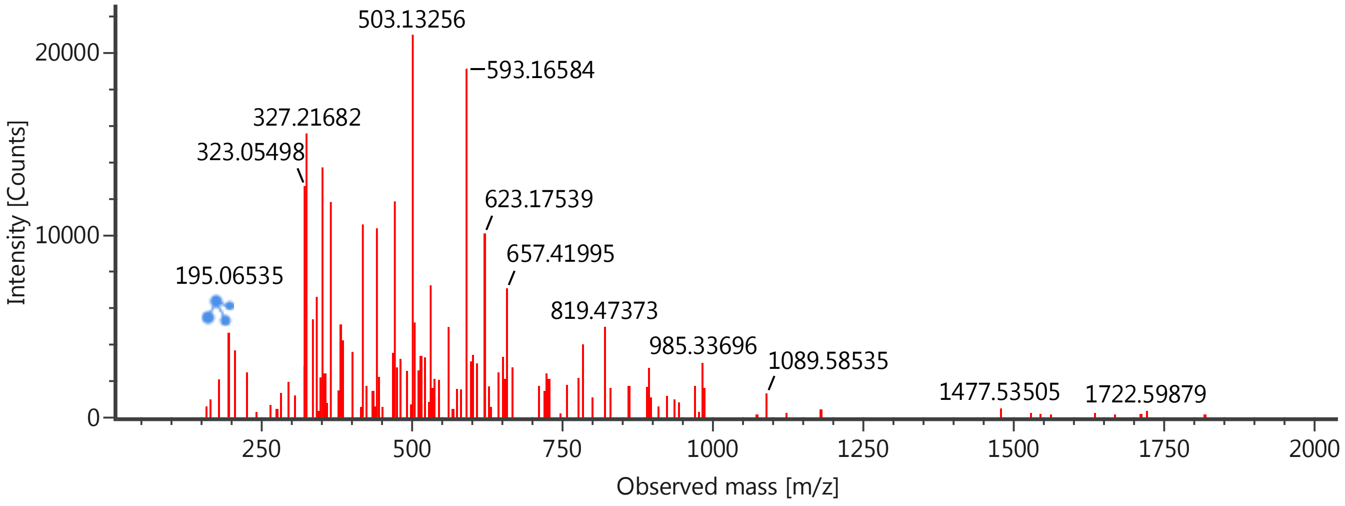

Component name:quercetin

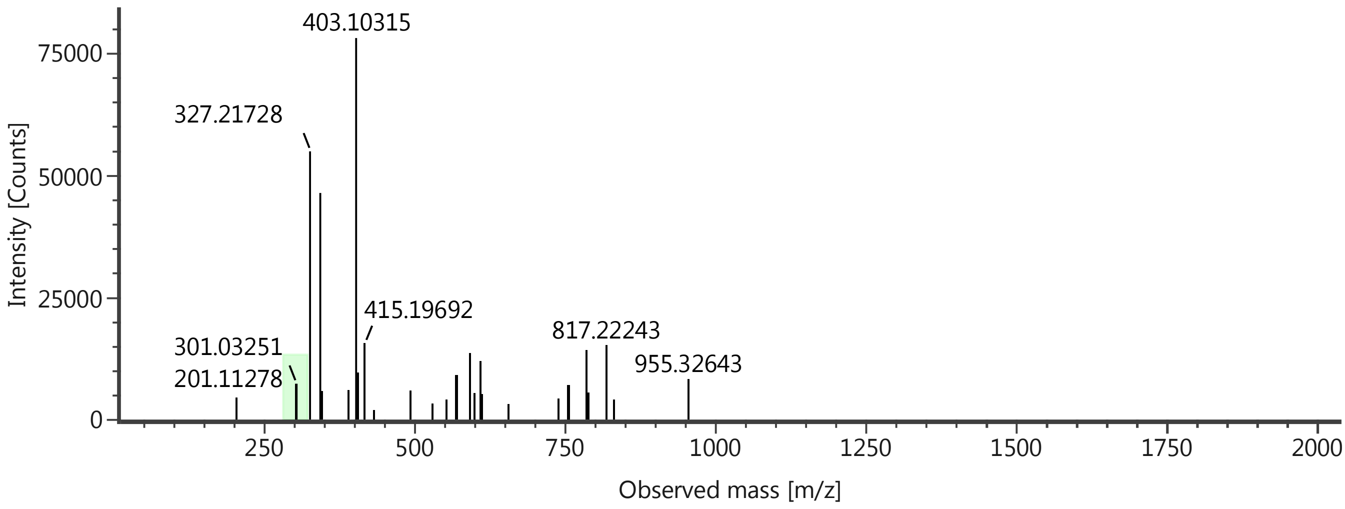

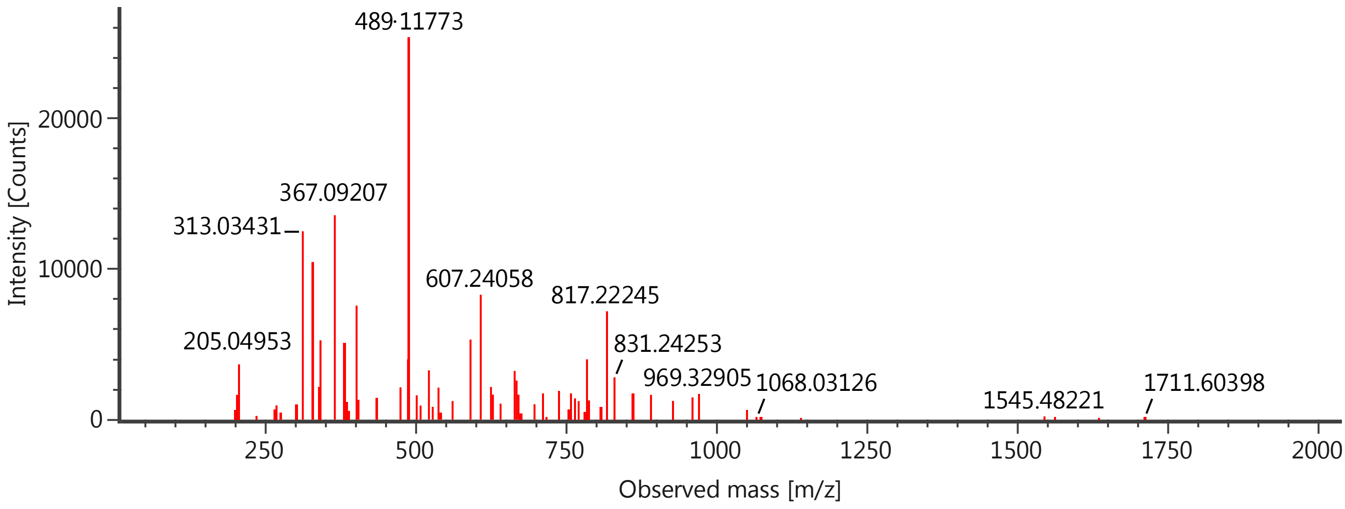

Component name: 7-hydroxy-4'-methoxy isof

lavone

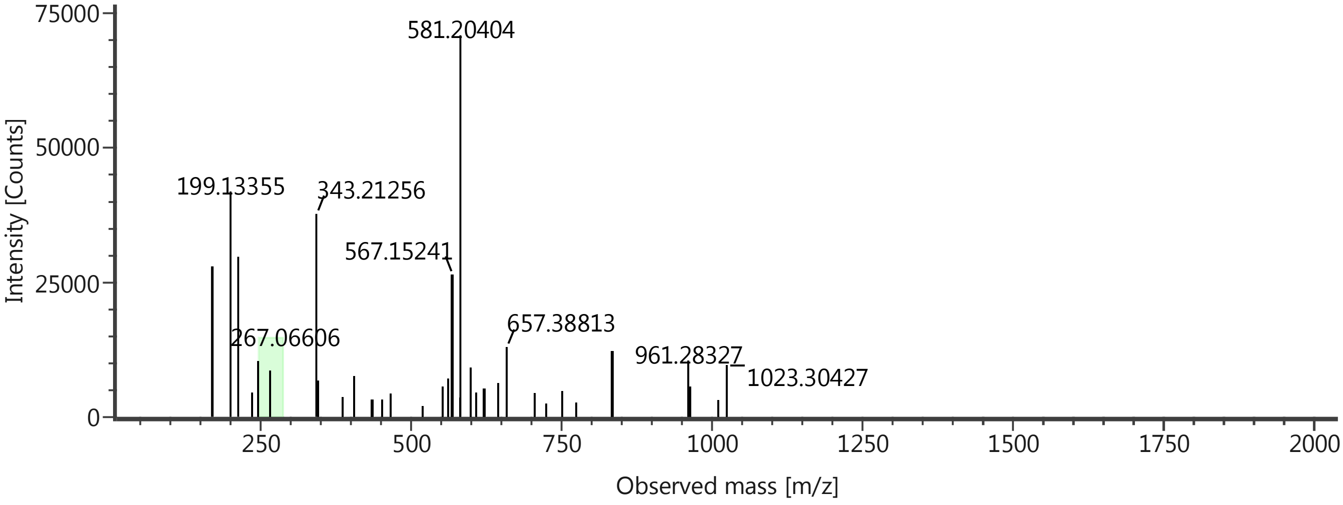

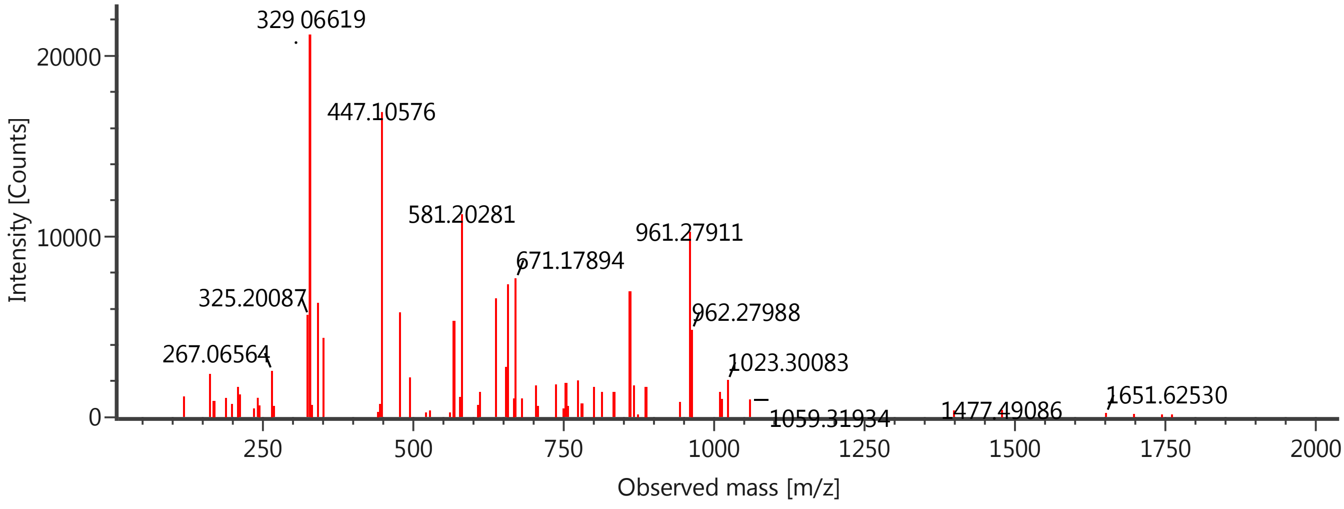

Component name:apigenin

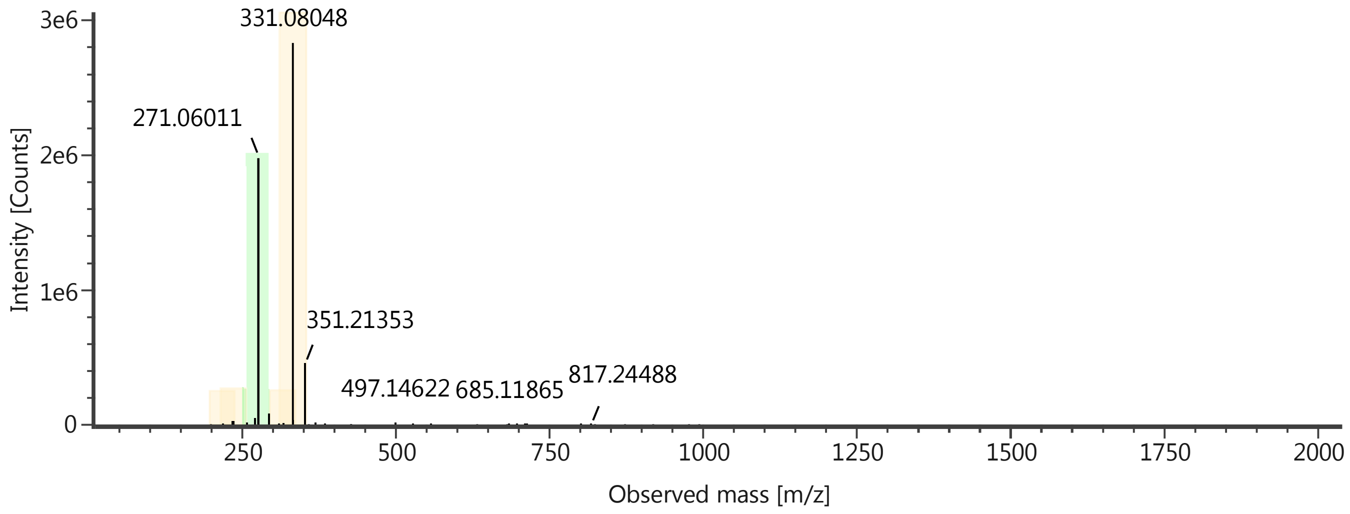

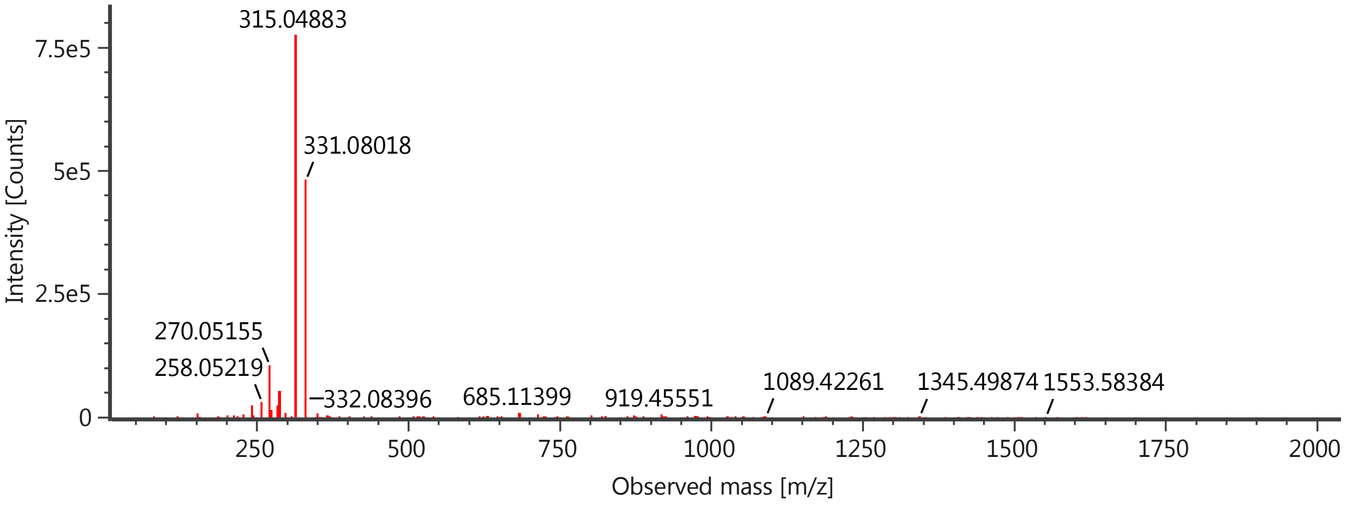

Component name:naringenin

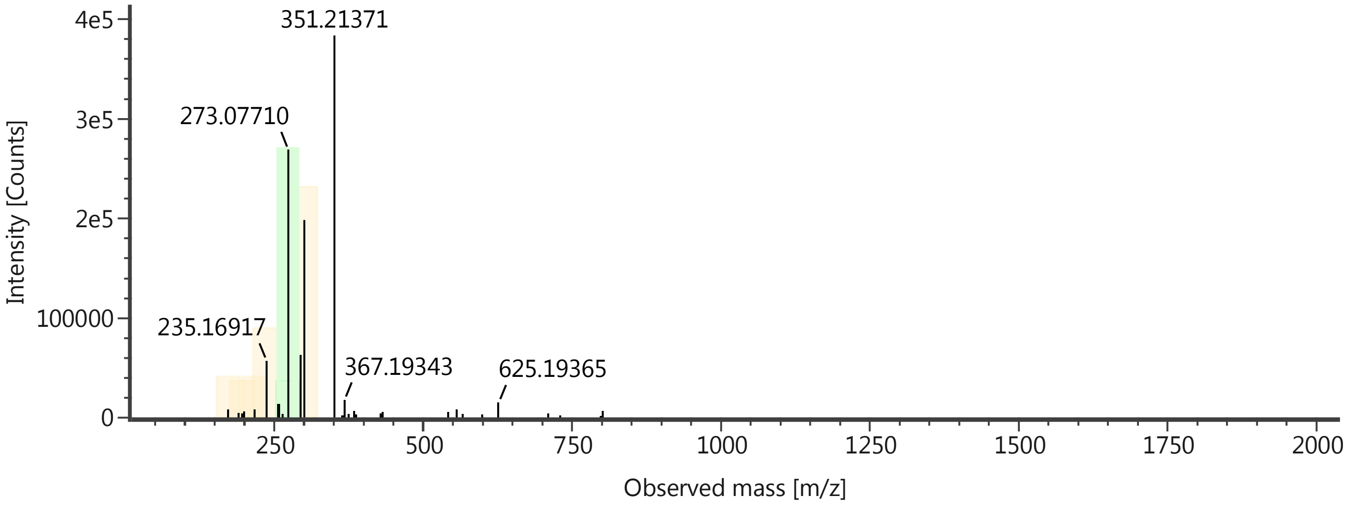

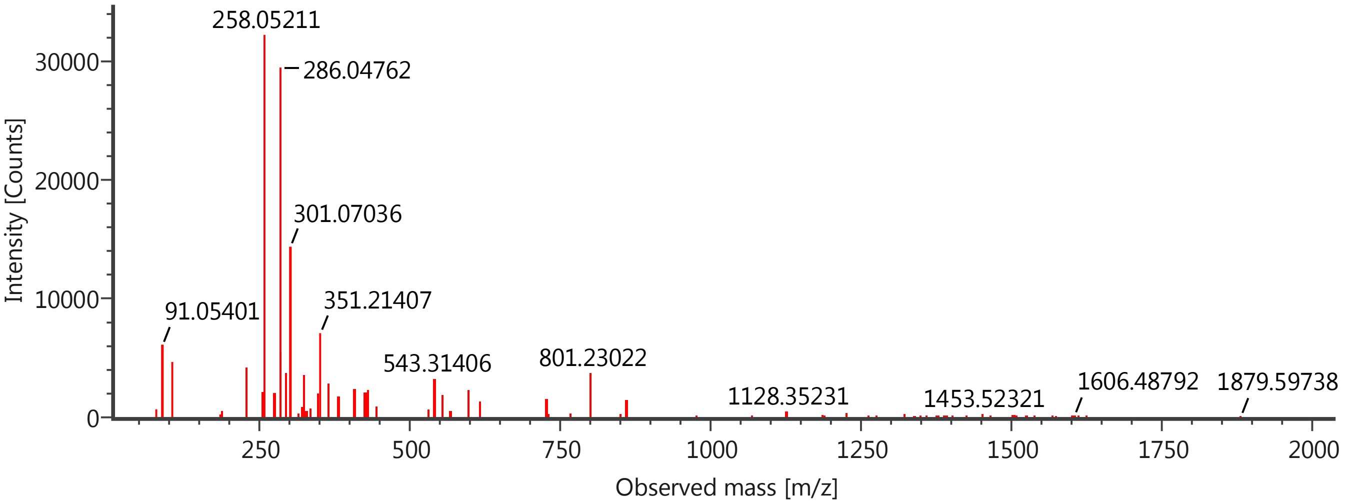

Component name:diosmetin

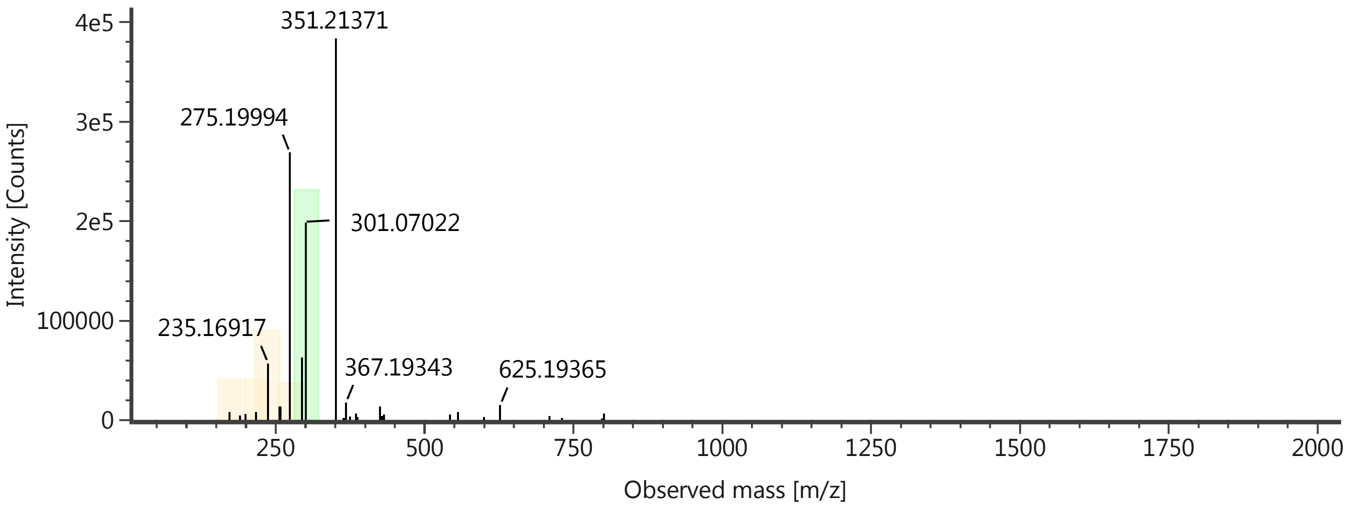

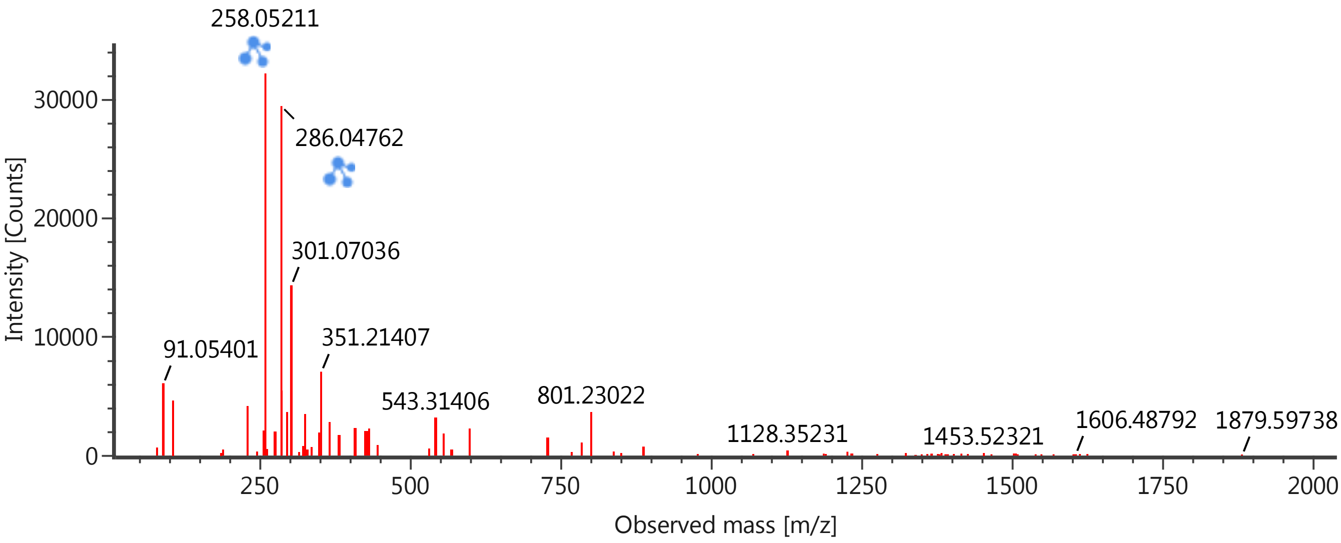

Component name:3,5-di-tert-butyl-4-hydrox

ybenzaldehyde

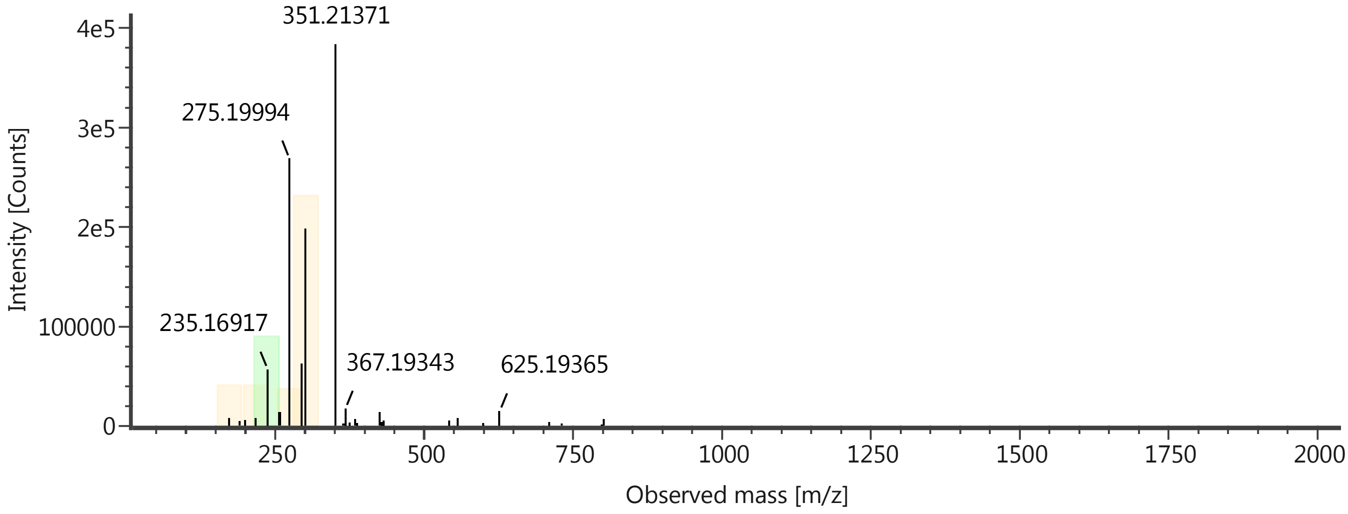

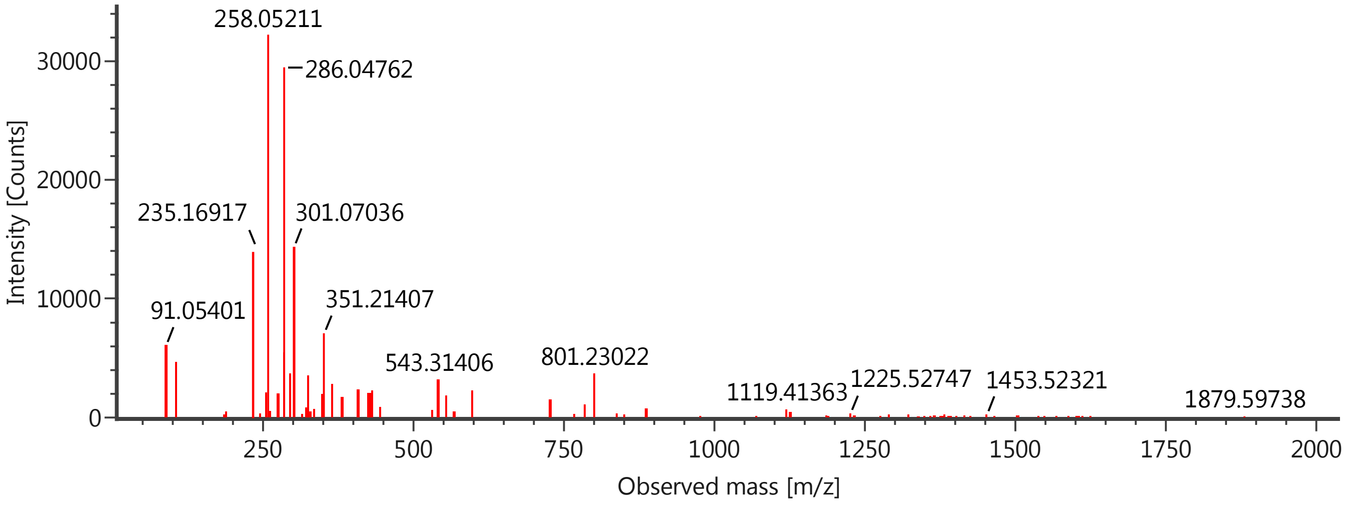

Component name:13-oxo-9E,11E-octadeca

dienoic acid

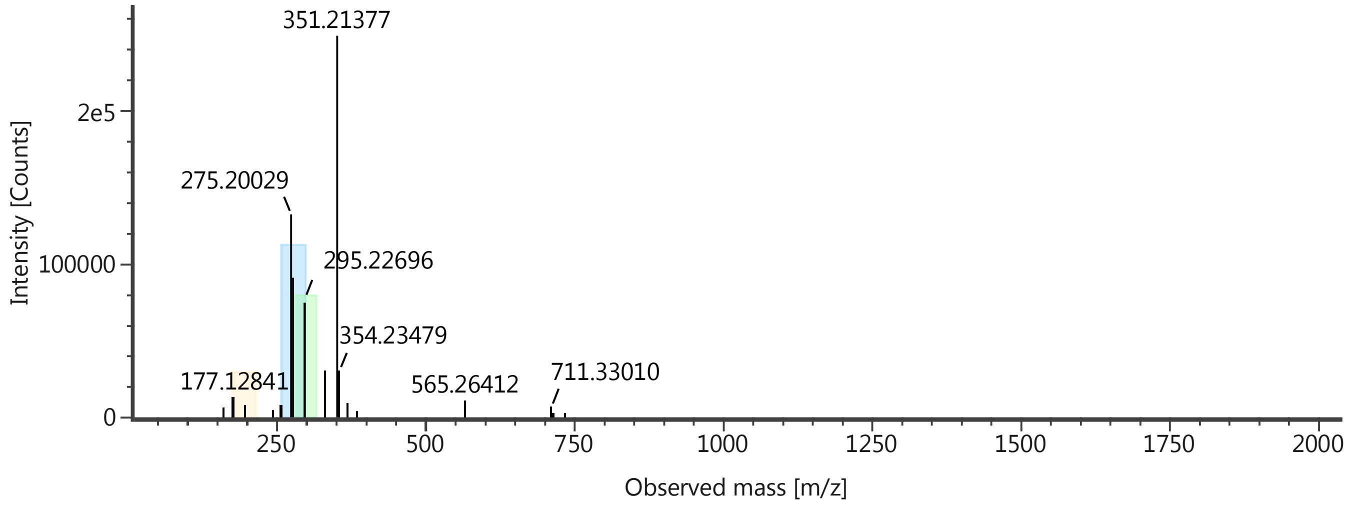

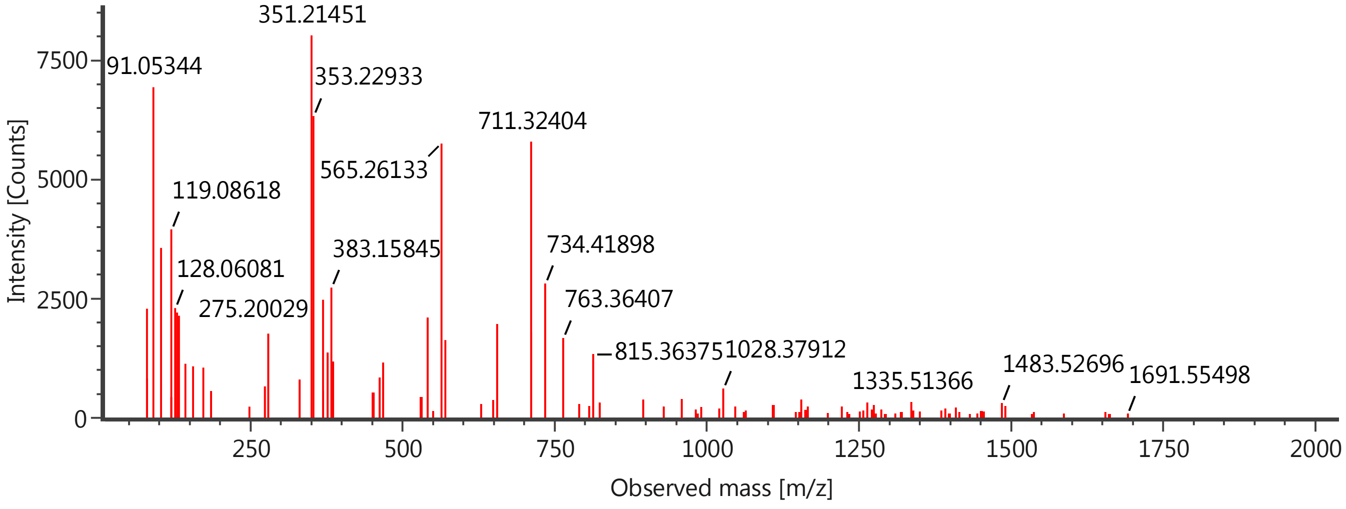

Supplement: Supplementary file 1 [file cimb-47-00779-s001.zip › Figure S1. The full MSMS spectra of LGB.pdf]
