# Supplementary material for: Bioactive Component Screening and Mechanistic Study of the Anti-Diabetic Activity of Lophatherum gracile Brongn Extract
Source: Curr Issues Mol Biol. 2025 Sep 19;47(9):779. doi: 10.3390/cimb47090779 (PMC12468491; doi:10.3390/cimb47090779)
Supplement: Supplementary file 1 [file cimb-47-00779-s001.zip › Table S1-Composition of the high-fat diet (HFD) used for establishing the T2DM model.pdf]

Table S1. Composition of the high-fat diet (HFD) used for establishing the T2DM model

No.	Compound	Content（%）
1	basic diet	57.8
2	sucrose	15.0
3	lard	10.0
4	cholesterol	2.0
5	sodium cholate	0.2
6	egg yolk	15.0
