# Supplementary material for: Bioactive Component Screening and Mechanistic Study of the Anti-Diabetic Activity of Lophatherum gracile Brongn Extract
Source: Curr Issues Mol Biol. 2025 Sep 19;47(9):779. doi: 10.3390/cimb47090779 (PMC12468491; doi:10.3390/cimb47090779)
Supplement: Supplementary file 1 [file cimb-47-00779-s001.zip › Table S2 Predicted chemical composition of bamboo leaf based on UPLC-Q-TOF-MSE.pdf]

Table S2. Predicted chemical composition of *Lophatherum gracile* Brongn based on UPLC-Q-TOF-MS^{E}

No.	RT (min)	Selected ion	MS (m/z)	Fragmentation (m/z)	Mass error (ppm)	Formula	Component name
1	4.34	$[M-H]^-$	179.0353	135.04532, 179.03515	1.1	$C_9H_8O_4$	caffeic acid
2	4.77	$[M-H]^-$	609.1467	447.09253, 327.05083, 144.04510	1	$C_{27}H_{30}O_{16}$	rutin
3	4.98	$[M-H]^-$	353.0879	191.05531	0.1	$C_{16}H_{18}O_9$	neochlorogenic acid
4	5.01	$[M-H]^-$	447.0938	357.06195, 327.05151, 299.05601, 133.02976	-3.1	$C_{21}H_{20}O_{11}$	isoorientin
5	5.35	$[M-H]^-$	593.1519	399.07177, 369.06137	1.3	$C_{27}H_{30}O_{15}$	kaempferol-3-O-β-D-rutinoside
6	5.47	$[M-H]^-$	463.0883	341.08742	0.2	$C_{21}H_{20}O_{12}$	quercetin-3-O-β-D-glucoside
7	5.46	$[M+H]^+$	165.0548	165.05413, 91.05346	1.3	$C_9H_8O_3$	p-Coumaric acid
8	5.5	$[M-H]^-$	337.0929	205.05007	0.1	$C_{16}H_{18}O_8$	(1R,3S,4R,5S)-1,3,4-trihydroxy-5-[(E)-3-(4-hydroxyphenyl)prop-2-enoyl]oxy-cyclohexane-1-carboxylicacid
9	5.59	$[M+H]^+$	144.0807	144.08069	-0.5	$C_{10}H_9N$	2-naphthylamine
10	5.59	$[M-H]^-$	151.0396	151.04018, 136.01669,	-2.9	$C_8H_8O_3$	vanillin
11	5.79	$[M+H]^+$	287.056	283.05959, 165.01822	3.5	$C_{15}H_{10}O_6$	luteolin
12	6.06	$[M-H]^-$	447.0938	327.05155, 357.06207, 297.04081, 285.04061	1.76	$C_{21}H_{20}O_{11}$	Orientin
13	6.22	$[M+H]^+$	579.1719	313.07018, 283.05998	1.8	$C_{27}H_{30}O_{14}$	apigenin 5-O-neohesperidoside
14	6.22	$[M-H]^-$	577.1568	373.09252, 223.06057	0.9	$C_{27}H_{30}O_{14}$	vitexin-2-O-rhamnoside
15	6.29	$[M-H]^-$	463.1244	373.09252, 285.11276, 205.05002	-0.3	$C_{22}H_{24}O_{11}$	homoeriodictyol 7-O-glucoside
16	6.38	$[M-H]^-$	607.1668	389.16027, 433.14857, 357.13340	-0.1	$C_{28}H_{32}O_{15}$	diosmin
17	6.41	$[M-H]^-$	343.0825	265.07098	0.4	$C_{18}H_{16}O_7$	usnic acid
18	6.48	$[M+H]^+$	431.1134	337.06999, 313.06996, 283.05956	1	$C_{21}H_{20}O_{10}$	vitexin
19	6.65	$[M+H]^+$	595.1679	343.08081, 207.06616	3.7	$C_{27}H_{30}O_{15}$	saponarin
20	6.78	$[M+H]^+$	147.0437	147.04371	-2.3	$C_9H_6O_2$	coumarin
21	6.83	$[M+H]^+$	155.0708	147.04368	3.6	$C_8H_{10}O_3$	vanillyl alcohol
22	6.97	$[M+H]^+$	289.0710	207.06587	1.3	$C_{15}H_{12}O_6$	eriodictyol

No.	RT (min)	Selected ion	MS (m/z)	Fragmentation (m/z)	Mass error (ppm)	Formula	Component name
23	7.19	$[M+H]^+$	303.2326	181.05065	2.4	$C_{20}H_{30}O_2$	abietic acid
24	7.36	$[M-H]^-$	367.1030	328.05701, 281.06670	-1.3	$C_{17}H_{20}O_9$	3-O-feruloylquinic acid
25	7.57	$[M-H]^-$	447.0933	447.09261, 329.06631, 314.04261	0.1	$C_{21}H_{20}O_{11}$	luteoloside
26	7.67	$[M+H]^+$	301.0709	301.07015, 105.06913	0.7	$C_{16}H_{12}O_6$	5,7,4'-trihydroxy-6-methoxy isoflavone
27	7.9	$[M-H]^-$	431.0981	367.06329, 279.08358, 183.01218	1.61	$C_{21}H_{20}O_{10}$	Isovitexin
28	7.90	$[M+H]^+$	331.0807	315.04915, 270.05202	-1.6	$C_{17}H_{14}O_7$	Tricin
29	8.38	$[M-H]^-$	447.0935	383.07677, 357.06121, 327.05068, 297.04018	0.6	$C_{21}H_{20}O_{11}$	luteolin-4'-o-glucoside
30	9.45	$[M-H]^-$	461.1092	339.05062, 327.21723	0.5	$C_{22}H_{22}O_{11}$	swertiajaponin
31	10.92	$[M-H]^-$	285.0401	285.03973, 133.02884	-1.2	$C_{15}H_{10}O_6$	kaempferol
32	11.05	$[M-H]^-$	401.1241	327.21682, 195.06535	-0.3	$C_{21}H_{22}O_8$	nobiletin
33	11.12	$[M-H]^-$	301.035	205.04953	-1.1	$C_{15}H_{10}O_7$	quercetin
34	12.19	$[M-H]^-$	267.0661	267.06564	-0.8	$C_{16}H_{12}O_4$	7-hydroxy-4'-methoxy isoflavone
35	12.93	$[M+H]^+$	271.0601	270.05155	0.1	$C_{15}H_{10}O_5$	apigenin
36	13.16	$[M+H]^+$	273.0771	258.05211, 91.05401	5	$C_{15}H_{12}O_5$	naringenin
37	13.18	$[M+H]^+$	301.0702	286.04762, 258.05211	-1.5	$C_{16}H_{12}O_6$	diosmetin
38	13.18	$[M+H]^+$	235.1692	235.16917	-0.4	$C_{15}H_{22}O_2$	3,5-di-tert-butyl-4-hydroxybenzaldehyde
39	14.01	$[M+H]^+$	295.2270	275.20029, 119.08618	0.7	$C_{18}H_{30}O_3$	13-oxo-9E,11E-octadecadienoic acid
