# Supplementary figures and images for: Bioactive Component Screening and Mechanistic Study of the Anti-Diabetic Activity of Lophatherum gracile Brongn Extract
Source: Curr Issues Mol Biol. 2025 Sep 19;47(9):779. doi: 10.3390/cimb47090779 (PMC12468491; doi:10.3390/cimb47090779)

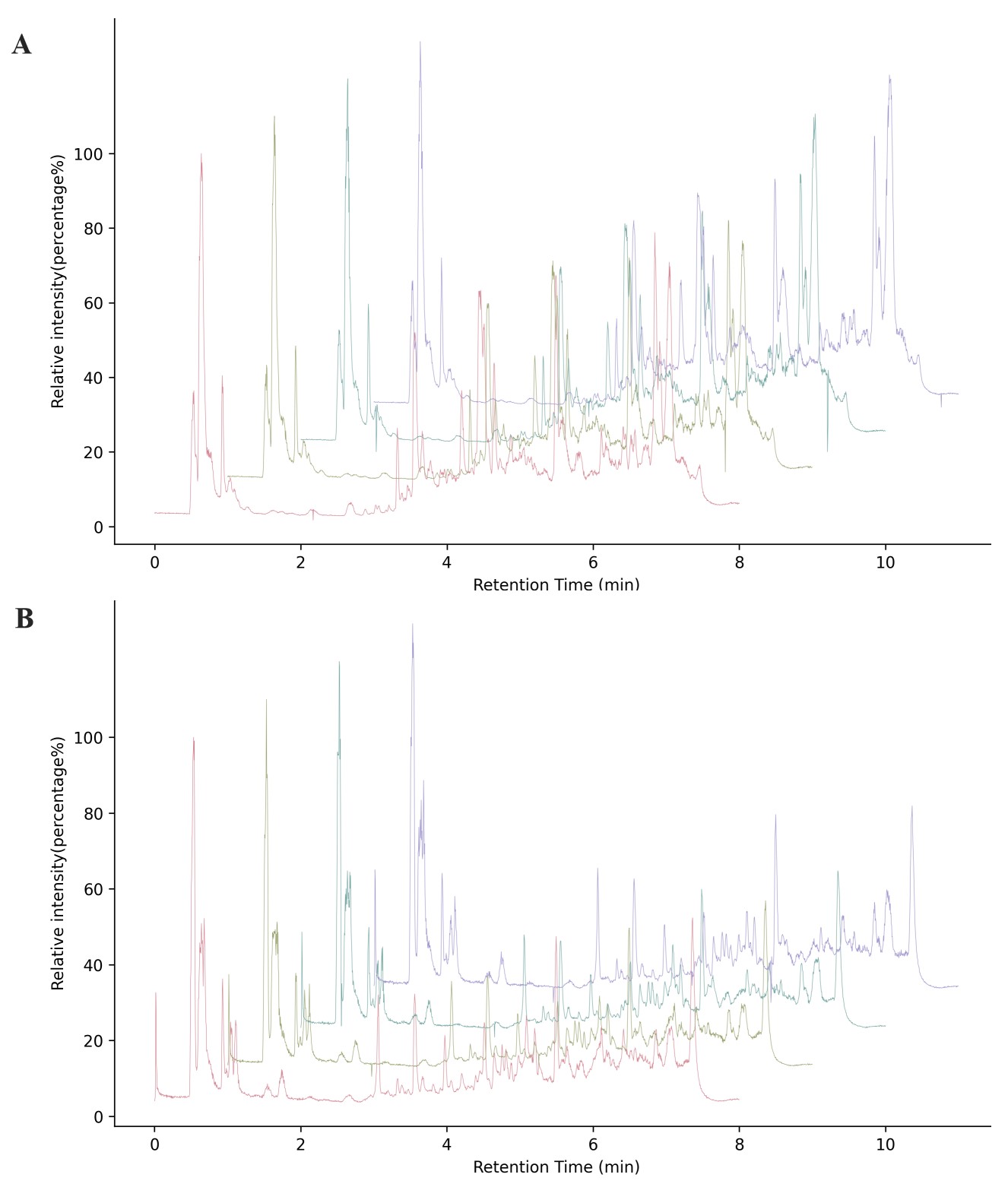

Supplement: Supplementary file 1 [file cimb-47-00779-s001.zip › Figure S2. Total ion chromatograms of quality control samples in non-targeted metabolomics analysis..jpg]

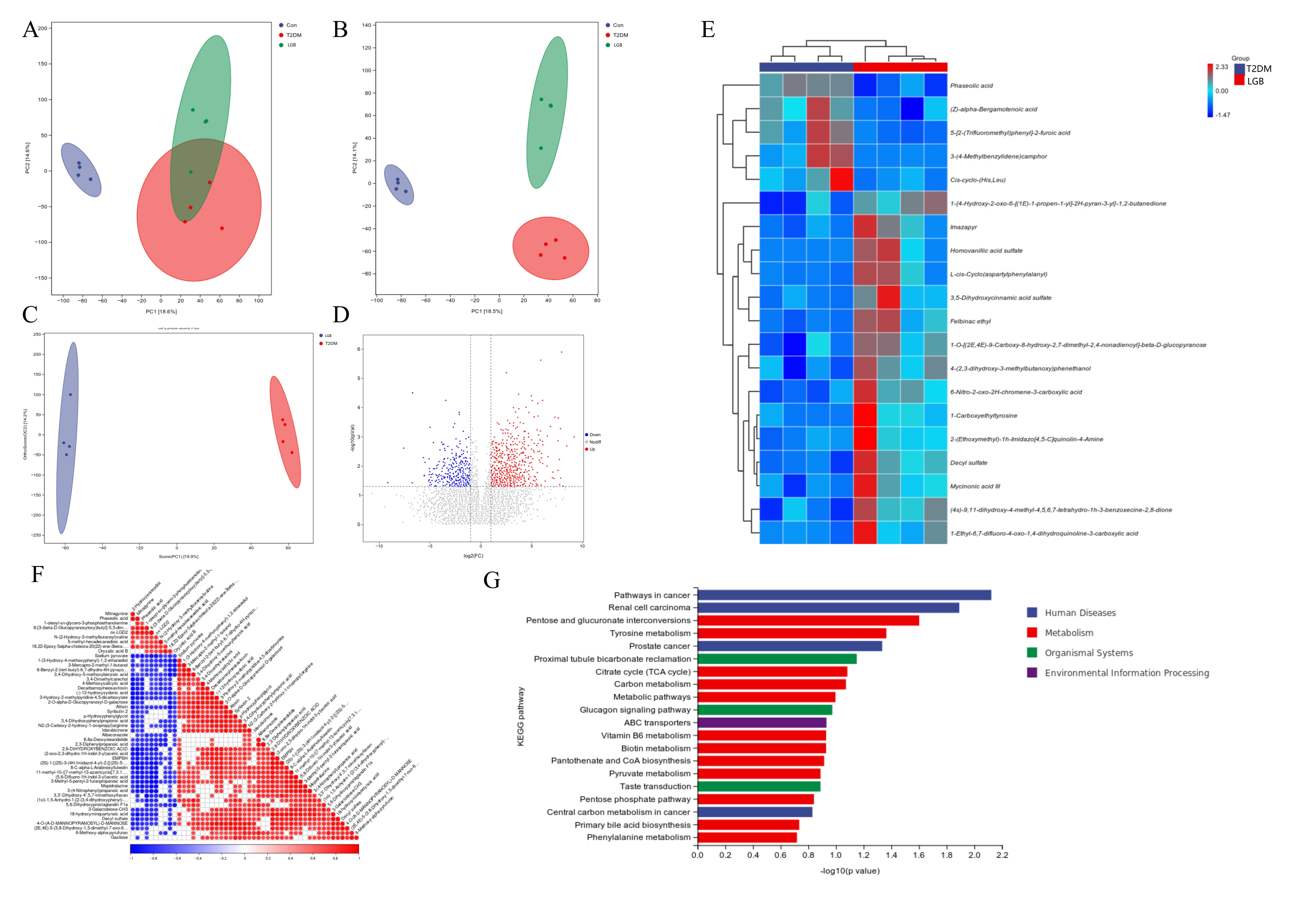

Supplement: Supplementary file 1 [file cimb-47-00779-s001.zip › Figure S3. Non-targeted metabolomics and associated pathway analysis in negative ion patterns..jpg]
